# Ancestral Regulatory Circuits Governing Ectoderm Patterning Downstream of Nodal and BMP2/4 Revealed by Gene Regulatory Network Analysis in an Echinoderm

**DOI:** 10.1371/journal.pgen.1001259

**Published:** 2010-12-23

**Authors:** Alexandra Saudemont, Emmanuel Haillot, Flavien Mekpoh, Nathalie Bessodes, Magali Quirin, François Lapraz, Véronique Duboc, Eric Röttinger, Ryan Range, Arnaud Oisel, Lydia Besnardeau, Patrick Wincker, Thierry Lepage

**Affiliations:** 1 UMR 7009 CNRS, Université de Pierre et Marie Curie (Paris 6), Observatoire Oceanologique, Villefranche-sur-Mer, France; 2 Génoscope (CEA), UMR8030, CNRS and Université d'Evry, Evry, France; University of Hawaii, United States of America

## Abstract

Echinoderms, which are phylogenetically related to vertebrates and produce large numbers of transparent embryos that can be experimentally manipulated, offer many advantages for the analysis of the gene regulatory networks (GRN) regulating germ layer formation. During development of the sea urchin embryo, the ectoderm is the source of signals that pattern all three germ layers along the dorsal-ventral axis. How this signaling center controls patterning and morphogenesis of the embryo is not understood. Here, we report a large-scale analysis of the GRN deployed in response to the activity of this signaling center in the embryos of the Mediterranean sea urchin *Paracentrotus lividus*, in which studies with high spatial resolution are possible. By using a combination of in situ hybridization screening, overexpression of mRNA, recombinant ligand treatments, and morpholino-based loss-of-function studies, we identified a cohort of transcription factors and signaling molecules expressed in the ventral ectoderm, dorsal ectoderm, and interposed neurogenic (“ciliary band”) region in response to the known key signaling molecules Nodal and BMP2/4 and defined the epistatic relationships between the most important genes. The resultant GRN showed a number of striking features. First, Nodal was found to be essential for the expression of all ventral and dorsal marker genes, and BMP2/4 for all dorsal genes. Second, *goosecoid* was identified as a central player in a regulatory sub-circuit controlling mouth formation, while *tbx2/3* emerged as a critical factor for differentiation of the dorsal ectoderm. Finally, and unexpectedly, a neurogenic ectoderm regulatory circuit characterized by expression of “ciliary band” genes was triggered in the absence of TGF beta signaling. We propose a novel model for ectoderm regionalization, in which neural ectoderm is the default fate in the absence of TGF beta signaling, and suggest that the stomodeal and neural subcircuits that we uncovered may represent ancient regulatory pathways controlling embryonic patterning.

## Author Summary

Echinoderms (sea urchins, starfish, etc.) are marine invertebrates that share a close ancestry with vertebrates. Their embryos offer many advantages for the analysis of transcriptional circuits that control developmental programs. During early development of the common sea urchin *Paracentrotus lividus*, a signaling center located within the ventral ectoderm sends two key signals, Nodal and BMP2/4, that control patterning of the embryo along the whole dorsal-ventral axis. How this signaling center works is not understood. We have conducted a large-scale functional analysis of the genes responsible for patterning of the ectoderm along the dorsal-ventral axis. We identified direct targets of Nodal and BMP2/4 and identified several key regulators that mediate the effects of these factors and drive essential and probably ancient regulatory circuits that together constitute a transcriptional program controlling morphogenesis of the embryo. In addition, we uncovered a striking parallel between the mouse embryo and the sea urchin embryo by showing that in both models a neurogenic ectoderm is the default state of ectoderm differentiation in the absence of Nodal and BMP signaling. Our results support the idea that inhibition of Nodal and BMP signaling was probably an ancient mechanism to specify neural cells in the ancestor of vertebrates.

## Introduction

It is becoming increasingly apparent that most developmental processes are controlled by dozens or hundreds of regulatory genes assembled into complex gene regulatory networks (GRNs), rather than by a small number of master genes. By describing the functional relationships between these genes, GRNs allow integration of various levels of information on the activity of transcription factors and signaling pathways that regulate developmental processes. Over the last few years, a number of GRNs have been elucidated, including regulatory networks that drive specification of germ layers or organs in various organisms [1]–[7].

Sea urchin embryos offer many advantages for GRN analysis [8]. Unlike vertebrates, sea urchin embryos have a relatively small number of cells (about 800 cells in a gastrula) are fully transparent, and their embryos, available in huge number, develop rapidly as free-swimming larvae. A panoply of techniques is available for the functional analysis of developmental genes including treatments with pharmacological inhibitors and exogenous ligands, microinjection of antisense morpholino oligonucleotides for gene loss of function, and overexpression of mRNA for gain of function. Analysis of the first full sea urchin genome sequence from *Strongylocentrotus purpuratus* has revealed that echinoderms have a vast genetic repertoire but a low level of genetic redundancy, with almost all developmental regulatory genes being present as single copy [9]. Furthermore the sea urchin embryo has a rich history of experimental embryology and a wealth of biological knowledge is available on various aspects of its development. Finally, echinoderms occupy a basal position within the deuterostome lineage and are more related to chordates than most other invertebrate phyla. These various properties mean that echinoderms are a key phylum to study the evolution of developmental mechanisms and to understand the evolutionary origin of certain features of the chordate body plan. Axis specification has been extensively studied in the sea urchin [10]. Pioneer studies on endomesoderm patterning have shown that it is possible to dissect a complex GRN without the use of classical genetics by combining cis-regulatory and functional analysis, embryological, cell biological and genomic/computational approaches [11]. However, while considerable knowledge is available regarding the functional relationships between genes controlling specification of the territories along the animal vegetal axis, much less was known until recently on the genes that regulate ectoderm patterning and morphogenesis of the embryo along the dorsal-ventral axis. This gap started to be filled recently by the identification in *Paracentrotus lividus* of the TGFβ Nodal, Univin, and BMP2/4 as key regulators of ectoderm patterning [12]–[15]. Nodal is expressed zygotically, starting at the 32-cell stage. Its expression is initially very broad then it is rapidly restricted to a discrete sector of the ectoderm that corresponds to the presumptive ventral ectoderm. The restricted expression of *nodal* is so far the earliest known regional difference in zygotic gene expression detectable along the dorsal ventral axis. However, experiments performed at the beginning of the century have shown that as early as the 8-cell stage, respiratory gradients, visualized by mitochondrial cytochrome oxidase activity, prefigure the dorsal-ventral axis of the early embryo [16]. In addition, orientation of the dorsal-ventral axis can be biased by using respiratory inhibitors or by culturing embryos in hypoxic conditions [17]–[19]. Recent studies reported that mitochondria are asymmetrically distributed in some batches of eggs of *Strongylocentrotus purpuratus* with the ventral side displaying the highest concentration, and that microinjection of purified mitochondria can bias orientation of the dorsal-ventral axis [20], [21]. A possible link between the transcriptional activation of *nodal* and these redox gradients is suggested by the finding that the stress activated kinase p38 is required for *nodal* expression [22]. An attractive model therefore emerges in which an asymmetry in the distribution of mitochondria may generate a redox gradient, which would activate p38 anisotropically leading to the spatially restricted expression of *nodal*. However, strong experimental evidence supporting this model are presently lacking and experimental manipulations that perturb the redox gradient have very modest effects on the spatial expression of *nodal* [21] (Thierry Lepage unpublished results). If the role of redox gradients in the establishment of *nodal* expression is still unclear, in contrast, the role of a reaction diffusion mechanism, which involves a short range Nodal positive autoregulation and a long range inhibition mechanism by the Nodal antagonist Lefty, is probably essential to convert a subtle initial anisotropy into a sharply defined pattern [12].

Overexpression of *nodal* strongly ventralizes the embryos and largely mimics the effects of treatments with nickel chloride [23], knockdown of Nodal function using morpholinos or by overexpressing *lefty*, completely eliminates dorsal-ventral polarity and results in embryos with disorganized skeletal elements, no mouth and a straight archenteron. The same, strongly-radialized, phenotypes are obtained by blocking translation of the *univin* transcript which encodes a Vg1/GDF1 ortholog expressed maternally [14], suggesting that Univin may either act upstream of *nodal* expression or that it may heterodimerize with Nodal as suggested in vertebrates [24], [25]. Intriguingly, in the absence of Nodal, not only is the expression of ventral marker genes such as *brachyury*, *goosecoid* or *lefty* abolished, but the expression of dorsal marker genes such as *tbx2/3* and of the novel transmembrane protein *29D* is suppressed as well [13]. As a consequence, most of the ectoderm (except the ectoderm surrounding the animal and vegetal poles) of Nodal morphants differentiates into a thick ectoderm consisting of cuboidal ciliated cells that morphologically resembles the neurogenic ectoderm of the ciliary band. Injection of synthetic mRNA encoding either Nodal or an activated Nodal receptor into one blastomere of Nodal morphant embryos at the 8-cell stage is sufficient to rescue both the ventral and the dorsal side of these embryos, indicating that a distinct relay molecule specifies dorsal fates. This relay molecule was recently identified as BMP2/4, which is transcribed in the ventral ectoderm downstream of Nodal signaling, has a strong dorsalizing activity when overexpressed, and mediates the “rescue” of dorsal structures when Nodal signaling pathway is ectopically activated in a cell-autonomous manner in a Nodal loss of function background [26]. Furthermore, despite its ventral transcription, BMP2/4 has been shown to trigger receptor mediated signaling exclusively on the dorsal side of the embryo. Based on this series of findings, a basic model for sea urchin embryo dorso-ventral patterning emerges in which the dorsal ectoderm is induced by BMP2/4 signals emanating from the opposite side of the embryo. The ventral side produces inducing factors such as Nodal and BMP2/4 but it is also a source of inhibitors such as Lefty, which restricts Nodal signaling to the ventral side, and Chordin, which prevents BMP2/4 signaling in the ventral ectoderm. In the absence of *lefty* function, Nodal signaling is unrestricted and propagates throughout a large belt of cells surrounding the embryo while in the absence of *chordin*, ectopic BMP2/4 signaling occurs on the ventral side and causes abnormal patterning of the embryo [12], [26]. Therefore, in the sea urchin as in vertebrates patterning of the embryo critically relies on sequential inductive events mediated by Nodal and BMP2/4 and on the interplay between ligands and their antagonists. However, in the sea urchin embryo, both the ligands (Nodal and BMP2/4) and their antagonists (Chordin and Lefty) are co-expressed in the ventral ectoderm, which may represent a D/V organizer, and D/V patterning requires translocation of BMP2/4 from the ventral side where it is produced to the dorsal side where it activates its receptor.

Another pathway that plays a crucial role in ectoderm patterning is the Wnt pathway. Wnt signaling from the vegetal pole region is required to restrict formation of the animal pole domain. The animal pole domain is a small ectodermal territory made of thick ciliated ectoderm that forms in the apical region of the embryo. This *six3* expressing neurogenic territory appears to be specified at mesenchyme blastula stage and is thought to be resistant to Wnt and TGF beta signaling [10], [13], [27], [28]. When the Wnt pathway is blocked by overexpression of cadherin or of a dominant negative form of TCF, the animal plate expands towards the vegetal pole and most of the ectoderm differentiates into neuroectodem, which contains scattered serotonergic neurons normally restricted to the animal plate region [27]. In contrast, inhibition of Nodal/Vg1/Activin signaling with a pharmacological inhibitor of the Nodal receptor causes formation of a thickened ciliated ectoderm, but this ciliated ectoderm does not appear to be specified as animal plate ectoderm since serotonergic neurons remain localized to the animal pole in these embryos. Instead, this ectoderm may have a ciliary band like identity as first proposed by Duboc et al. [13]. This idea is supported by the finding that the ectoderm of Nodal morphants abundantly expresses the ciliary band marker *tubulinß3* [13] and by the presence of ectopic neurons as revealed by staining for the pan-neural marker synaptotagmin [27]. However, more in depth analysis of the specification state of this ectoderm in the absence of Nodal signaling is required to further test this idea.

Deciphering the gene regulatory network that controls patterning of the ectoderm is of special importance for several reasons. The first reason is that patterning of all three germ layers relies on the activity of a signaling center located in the ventral ectoderm and analyzing how this signaling center works is essential to understand how dorsal ventral polarity of the embryo is established. Another reason is that, despite a wealth of information available on establishment of D/V polarity during normal and regulative development, the GRN that controls specification of the main ectodermal territories (ventral ectoderm, dorsal ectoderm and ciliary band) remains incompletely described and the molecular mechanisms involved in regionalization of the embryo along the D/V axis in normal and perturbed embryos have just started to be investigated [29]. A third reason to study the D/V GRN comes from the basal evolutionary position of echinoderms within the deuterostome superclade, and of the notion that studying D/V axis formation in echinoderms will contribute to better understand the evolution of the patterning mechanisms that shaped the deuterostome body plan. Indeed, recent studies have shown that this GRN relies extensively on cell interactions mediated by TGF beta family members such as Nodal, Univin/Vg1 and BMP2/4, molecules that play crucial roles during vertebrate development [13], [14], [26]. Finally, since major morphogenetic processes such as mouth formation, skeleton formation and elongation of the arms and apex of the larva occur along the D/V axis, dissecting the D/V GRN offers the promise to study how morphogenetic processes are encoded in the genomic program of development. This will help to fill the gap that presently exists between our understanding of cell fate specification and our knowledge of how genes work together to regulate morphogenesis.

We previously described the core of the GRN that acts downstream of Nodal and is responsible for patterning of the ectoderm along the dorsal-ventral axis [13]. We showed that on the ventral side, Nodal acts at the top of this GRN by regulating the expression of *lefty*, *bmp2/4*, *goosecoid* and *brachyury* while on the dorsal side BMP2/4 activates the expression of *tbx2/3*. Although the functional relationships between these key genes was elucidated in this initial study, recent molecular screens conducted by us (Thierry Lepage unpublished) and others [30] revealed that many more downstream genes are likely involved in patterning of the ectoderm along the dorsal ventral axis. A large scale effort to dissect the ectoderm GRN in *S. purpuratus* was recently published by Su and colleagues who used the nanostring technology to monitor the effects of gene perturbations [29]. However, this technique, which measures RNA concentrations in whole embryos, lacks the spatial resolution that is required to analyze the changes in the complex spatial expression patterns of many developmental genes.

To understand better how the ectoderm of the sea urchin embryo is patterned by Nodal and BMP2/4 signals and to expand our provisional GRN, we conducted a large-scale study. Using a combination of gain of function and loss of function studies, and taking advantage of the amenability of *Paracentrotus lividus* embryos to detailed phenotypic analyses and in situ hybridization studies, we analyzed at high spatial resolution the expression and regulation by Nodal and BMP2/4 of 18 transcription factors and 8 signaling molecules that displayed a restricted expression along the D/V axis. Using an assay with recombinant proteins, we identified direct targets of Nodal and BMP2/4. Finally, by conducting a large-scale analysis of the epistatic relationships between these genes, we were able to start ordering them into a hierarchy and to identify key regulators acting downstream of Nodal and BMP2/4. Not only our results uncover novel and probably ancient regulatory circuits that drive morphogenetic processes such as mouth formation and neural induction, but they elicit a model for patterning of the ectoderm in which two successive inductive events regionalize the ectoderm into three territories: the ventral ectoderm that is specified by Nodal, the dorsal ectoderm that is specified by BMP2/4 and the neurogenic ectoderm of the ciliary band, which forms between the ventral and the dorsal ectoderm in a region protected from Nodal and BMP signaling. In addition, these findings highlight a striking parallel between the mouse embryo and the sea urchin embryo by showing that in both models a neurogenic ectoderm is the default state of ectoderm differentiation in the absence of Nodal and BMP signaling. Our analysis provides a picture of this GRN significantly different from that proposed by Su et al. in *S.purpuratus* and stresses the importance of the spatial resolution level in the analysis of gene regulatory networks in early embryos.

## Results

### Novel markers of regional differences in gene expression within the ectoderm

**Figure 1:**
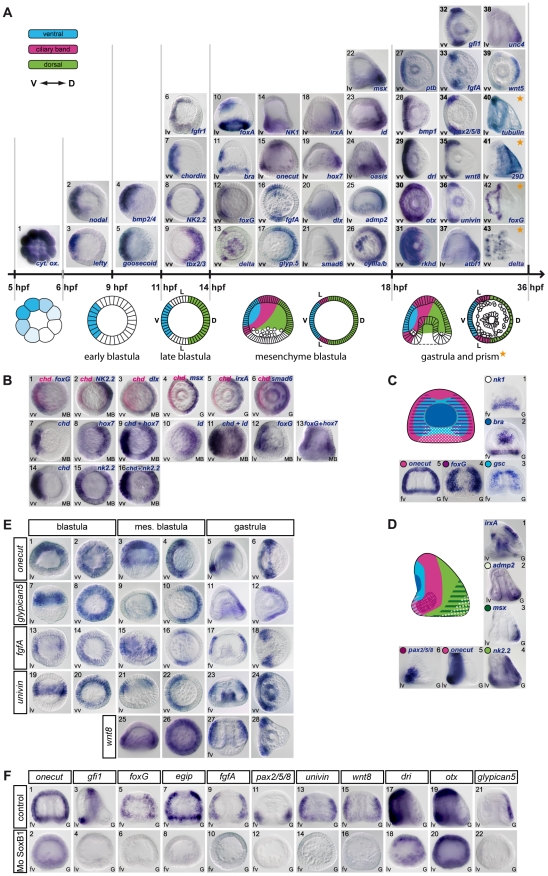
Gene expression profiles of transcription factors and signaling molecules analyzed in this study. (A) Spatial and temporal expression profiles. The expression of 21 genes encoding transcription factors (*goosecoid, nk2.2, tbx2.3, nk1, foxA, brachyury, foxG, onecut/hnf6, irxA, hox7, dlx, smad6, msx, id, oasis, deadringer, otx, gfi, pax2/5/8, atbf1, unc4*), 13 signaling molecules (*nodal, lefty, bmp2/4, chordin, fgfA, fgfr1, glypican5, admp2, bmp1, wnt8, univin, wnt5, Delta*), 2 RNA binding proteins (*rkhd*, *ptb*), 3 differentiation genes (*cyIIIa*, *29D*, *tubulinß3*) and a mitochondrial gene (*cytochrome oxidase*) is depicted above a scheme of early development of the sea urchin embryo. The genes are classified into 5 groups according to the timing of their expression. (1) Maternal cytochrome oxidase transcripts show a graded distribution in cleaving embryos. (2,3) Starting at the early blastula stage, *nodal* and *lefty* are the first zygotic genes to be expressed in a restricted pattern along the D/V axis, followed by *bmp2/4* and *goosecoid* before hatching (4,5). (6–9) After hatching, expression of *fgfr1* and *chordin* is initiated ventrally while *nk2.2* and *tbx2/3* start to be expressed dorsally. (10–26) At mesenchyme blastula stage *foxA*, *brachyury*, *foxG*, *Delta*, *nk1*, *onecut*, *fgfA*, *glypican5*, *irxA*, *hox7*, *dlx*, *smad6*, *msx*, *id*, *oasis*, *admp2*, and *CyIII* start to be expressed in a restricted pattern. (27–36) A restricted expression is established slightly later at the early gastrula stage for *ptb*, *bmp1*, *dri*, *otx*, *rkhd*, *gfi1*, *pax2/5/8*, *wnt8*, *univin*, and for *atbf1*, *unc4* and *wnt5* (37–39). (40–43) *tubulinß3* and *29D* are expressed in the presumptive ciliary band or dorsal ectoderm starting at prism/early pluteus stages while *foxG* is expressed in a ventral subdomain of the ciliary band and *Delta* in individual cells within the ciliary band and facial ectoderm. (B) (1) Two color double in situ hybridization showing that *foxG* is expressed ventrally. (2,15,16) *nk2.2* is expressed in two discrete regions along the D/V axis. At the late blastula stage, the territory with the strongest expression is located on the ventral side while at mesenchyme blastula stage, the highest level of transcripts is detected on the dorsal side. (3–6) Two color double in situ hybridization with *chordin* shows that *dlx*, *msx*, *irxA* and *smad6* are expressed on the dorsal side. (7–13) One color double in situs hybridizations confirm that *id* and *hox7* are expressed on the dorsal side. (C) At late gastrula stage, differences in expression of marker genes along the A/V and D/V axes identify differently specified territories within the ventral ectoderm. (1)The *NK1* homeobox gene is expressed in a trapezoidal domain that abuts the stomodeal domain and the lateral ectodermal regions that express *fgfA* and *pax2/5/8*. (2) *brachyury* is expressed in a group of 30 cells located in the center of the ventral ectoderm and that likely constitute the stomodeal precursors. (3–5) This group of cells is surrounded by a large belt of *goosecoid* and *foxG* expressing cells that are themselves surrounded by a thinner belt of *onecut/hnf6* expressing ciliary band precursors. (D) Nested expression domains are also apparent within the dorsal ectoderm and ciliary band. (1) *irxA* is expressed in a medial sub domain of the dorsal ectoderm that abuts the ciliary band. Note that *irxA* is also expressed in the stomodeal region at this stage. (2,3) Genes like *admp2* and *msx* are expressed in nested patterns in the dorsal most region that corresponds to the presumptive apex of the larva. (4) *nk2.2*, like *tbx2/3*, is expressed in most cells of the dorsal ectoderm. (5,6) *Onecut* is expressed in the whole ciliary band, while *pax2/5/8* is expressed in the vegetal portion of the presumptive ciliary band. (E) A set of ectodermal genes including *onecut/hnf6*, *glypican5*, *fgfA*, *univin* and *wnt8* are expressed broadly in the ectoderm at blastula stages and subsequently restricted to either the dorsal ectoderm (*glypican5*) or the ciliary band (*onecut/hnf6*, *fgfA*, *univin*, *wnt8*). (F) Expression of several ciliary band genes including *onecut*, *gfi1*, *foxG*, *egip*, *fgfA*, *pax2/5/8*, *univin*, *wnt8*, *dri*, *otx* and of the dorsal marker *glypican5* critically relies on the activity of the transcription factor SoxB1. V, ventral, D, dorsal, L, lateral. lv, lateral view, vv, vegetal pole view, fv, frontal view.

To elucidate the gene regulatory network that controls specification and patterning of the ectoderm in *Paracentrotus*, we first performed large scale in situ hybridization screens. In addition to a random screen initiated several years ago, which allowed us to characterize the expression of 4000 randomly selected cDNAs (Thierry Lepage unpublished), we screened a *P. lividus* EST database against *S. purpuratus* sequences encoding transcription factors and signaling molecules and analyzed the expression of all those that were expressed during development of the sea urchin embryo [30]–[34] (Table 1). This allowed us to assemble a list of 36 genes displaying a robust expression in either the ventral ectoderm, the dorsal ectoderm or in the ciliary band territory (Table 1) (Figure 1A, 1B). Genes expressed in the animal pole domain were largely excluded from this analysis since most of them do not display a restricted expression along the D/V axis. The expression patterns of a number of the genes presented in this study had previously been described at various degrees in *S. purpuratus* [30]–[34] but they had never been described in *Paracentrotus*. In addition, the expression of several genes analyzed here, including *smad6*, *gfi1*, *id*, *admp2*, *BMP1*, and *oasis* has not been described previously in either species.

**Table 1:** Genes examined in this study.

gene	maternal vs zygotic	onset of restricted expression in the ectoderm	spatial expression within the ectoderm	accession N°	Reference
***nodal***	zygotic only	64-cell stage (5h)	broad early then, presumptive ventral ectoderm until late gastrula, right side of ciliary band at prism and pluteus stages	**AY442295**	[13]
***bmp2/4***	zygotic only	128-cell stage (6h)	presumptive ventral ectoderm until late gastrula then shifts to the dorsal most ectoderm	**DQ536194**	[15], [36]
***lefty***	zygotic only	128-cell stage (6h)	broad early, presumptive ventral ectoderm until late gastrula then right side of ciliary band	**AY442296**	[13]
***univin***	maternal and zygotic	early blastula (7h)	equatorial ectoderm early, then progressively restricted to ciliary band	**DQ536195**	[125]
***gsc***	weak maternal and zygotic	hatching blastula (11h)	center of the presumptive ventral ectoderm until MB then gradually excluded from the presumptive stomodeum, periphery of the stomodeum at pluteus stage	**HM449798**	[35]
***tbx2/3***	zygotic only	swimming blastula (12h)	most of presumptive dorsal ectoderm	**AJ508929**	[40], [41]
***nk2.2***	zygotic only	swimming blastula (12h)	center of the presumptive ventral ectoderm and most of presumptive dorsal ectoderm	**HM449801**	[31]
***fgfr1***	maternal and zygotic	swimming blastula (12h)	presumptive ventral ectoderm	**DQ536196**	[26], [37]
***chordin***	zygotic only	swimming blastula (12h)	center of the presumptive ventral ectoderm until late gastrula then ventral ciliary band	**FJ976182**	[26], [38]
***foxA***	zygotic only	mesenchyme blastula (15h)	center of the presumptive ventral ectoderm then stomodeum	**EU263275**	[42]
***bra***	zygotic only	mesenchyme blastula (15h)	center of the presumptive ventral ectoderm then stomodeum	**J419790**	[43], [44]
***nk1***	zygotic only	mesenchyme blastula (15h)	ventral ectoderm adjacent to the blastopore	**HM449799**	[45]
***foxG***	zygotic only	mesenchyme blastula (15h)	periphery of the ventral ectoderm at mesenchyme blastula then strictly ciliary band	**HM449800**	[34]
***ptb***	maternal and zygotic	mesenchyme blastula (15h)	ventral ectoderm from mesenchyme blastula to prism stage	**DQ343157**	[52]
***smad6***	maternal and zygotic	mesenchyme blastula (15h)	most of presumptive dorsal ectoderm	**HM449802**	[36]
***glypican5***	maternal and zygotic	mesenchyme blastula (15h)	most of dorsal ectoderm, excluded from the apex starting at late gastrula	**HM449803**	[26]
***msx***	zygotic only	mesenchyme blastula (15h)	subdomain of the dorsal ectoderm, presumptive apex	**CAD56482**	[126]
***irxA***	zygotic only	mesenchyme blastula (15h)	subdomain of the dorsal ectoderm, excluded from presumptive apex , stomodeum starting at gastrula stage	**HM449804**	[31]
***dlx***	zygotic only	mesenchyme blastula (15h)	most of presumptive dorsal ectoderm	**HM449805**	[31]
***wnt5***	zygotic only	mesenchyme blastula (15h)	presumptive apex, also in presumptive ciliary band at gastrula	**HM449806**	[127]
***atbf1***	zygotic only	mesenchyme blastula (15h)	weak in ventral ectoderm, stronger in presumptive dorsal ectoderm	**AM200680**	[31]
***hox7***	zygotic only	mesenchyme blastula (15h)	most of presumptive dorsal ectoderm, retracts gradually to the apex	**HM449807**	[128]
***id***	zygotic only	mesenchyme blastula (15h)	most of presumptive dorsal ectoderm	**HM449808**	[30]
***creb3L3/oasis***	maternal and zygotic	mesenchyme blastula (15h)	most of presumptive dorsal ectoderm	**HM535633**	[30]
***CyIIIa/b***	maternal and zygotic	mesenchyme blastula (15h)	broad early then most of presumptive dorsal ectoderm	**AM564750**	[129]
***admp2***	zygotic only	mesenchyme blastula (15h)	most of presumptive dorsal ectoderm early, then vegetal ectoderm at gastrula stages	**HM449811**	[36]
***fgfA***	zygotic only	mesenchyme blastula (15h)	equatorial region at mesenchyme blastula then subdomain of the presumptive ciliary band	**EF157978**	[48]
***pax2/5/8***	zygotic only	mesenchyme blastula (15h)	subdomain of the presumptive ciliary band: lower part of the lateral ectoderm	**AF016884**	[48]
***wnt8***	zygotic only	mesenchyme blastula (15h)	ectoderm of the vegetal hemisphere at mesenchyme blastula stages then restricted to presumptive ciliary band	**HM449816**	[53]
***onecut***	maternal and zygotic	mesenchyme blastula (15h)	most of the ectoderm early, then restricted to ciliary band	**HM449812**	[46], [47]
***gfi1***	zygotic only	mesenchyme blastula (15h)	presumptive ciliary band	**HM449813**	[32]
***rkhd***	maternal and zygotic	early gastrula (20h)	ventral ectoderm +ciliary band starting at early gastrula stage	**DQ355836**	[52]
***dri***	zygotic only	early gastrula (20h)	presumptive ventral ectoderm +presumptive ciliary band	**HM449814**	[49]
***otx***	maternal and zygotic	early gastrula (20h)	presumptive ventral ectoderm +presumptive ciliary band	**HM449815**	[50]
***bmp1***	maternal and zygotic	early gastrula (20h)	ubiquitous at blastula stages then restricted to presumptive ventral ectoderm and ciliary band at gastrula stages	**HM449817**	[51]
***egip***	maternal and zygotic	early gastrula (20h)	presumptive ciliary band and presumptive apex starting at early gastrula	**HM449819**	[130]
***unc4***	zygotic only	gastrula (24h)	apex of dorsal ectoderm	**HM449809**	[31]
***29D***	zygotic only	prism (30h)	most of presumptive dorsal ectoderm, starting at prism	**HM449810**	[13]
***Tubulinß3***	maternal and zygotic	prism (30h)	animal pole region at blastula stages then presumptive ciliary band starting at prism stages	**HM449818**	[54]

The earliest asymmetrically distributed transcript that we identified in the in situ screens is the maternal transcript encoding mitochondrial cytochrome oxidase, with cleavage stage embryos frequently displaying a graded distribution of transcripts in the presumptive ectoderm (Figure 1A1). This asymmetrical distribution of a mitochondrial transcript likely reflects the asymmetrical distribution of mitochondria previously reported by Coffman and colleagues [20], [21]. At the zygotic level, the first signs of tissue regionalization within the ectoderm are seen at 64/128 cell-stage with *nodal* and *lefty* transcripts starting to accumulate in the presumptive ventral territory (Figure 1A2,3) [12], [13]. The second wave of zygotic genes displaying a restricted expression along the D/V axis starts at the prehatching blastula with *bmp2/4* and *goosecoid* starting to be transcribed in the ventral ectoderm rapidly followed by *fgfr1*, *chordin* and *nk2.2* at the swimming blastula stage (Figure 1A4–8) [15], . In *Paracentrotus*, there is no known example of genes displaying a restricted expression in the dorsal ectoderm before the swimming blastula stage. The first genes to be expressed in the dorsal ectoderm are *nk2.2* and *tbx2/3*, whose expression increases abruptly in the presumptive dorsal territory after hatching (Figure 1A8,9) [31], [40], [41]. These genes are therefore good candidates as immediate early targets of Nodal or BMP2/4 signaling and are likely to play an early role in specification of these territories. Soon after ingression of the primary mesenchyme cells, when the embryo acquires its bilateral symmetry, a third wave of zygotic genes starts to be expressed. This includes the largest number of genes such as *foxA*, *brachyury*, *foxG*, *Delta*, *NK1*, in the ventral ectoderm (Figure 1A10–14) [34], [42]–[45], *onecut/hnf6* and *fgfA* (Figure 1A15–16) [46]–[48] in the lateral ectoderm and *glypican5*, *irxA*, *hox7*, *dlx*, *smad6*, *msx*, *id*, *oasis*, *admp2* and *cyIII* in the dorsal ectoderm (Figure 1A17–26) [26], [30], [31], [36], [39]. Based on the timing of their expression, genes in this category are likely secondary targets of Nodal or BMP2/4 signaling.

Starting at the early gastrula stage, additional genes start to be expressed with a restricted pattern along the D/V axis, with *ptb* transcripts accumulating in the ventral ectoderm (Figure 1A27), *bmp1*, *deadringer* (*dri*), *otx*, and *rkhd* being expressed in a broad domain encompassing the ventral ectoderm and ciliary band territory (Figure 1A28–31) [13], [49]–[52], and *gfi1*, *pax2/5/8*, *wnt8*, *univin* transcripts starting to be expressed in the presumptive ciliary band (Figure 1A32–36) [32], [36], [48], [53]. Similarly, *atbf1*, *unc4*, *wnt5* start to be expressed in the dorsal ectoderm at the early gastrula stage. (Figure 1A37–39). Finally, at prism stage, *tubulinß3* transcripts accumulate in the presumptive ciliary band while transcripts encoding the sea urchin specific transmembrane protein *29D* accumulate in the presumptive dorsal ectoderm (Figure 1A 40,41) [13], [54].

At the mesenchyme blastula stage, *foxG* (also known as *Brain factor1* or *Bf1*) is expressed in two broad ventro-lateral stripes that largely overlap with the *goosecoid* expression territory (Figure 1A12), while Delta is first expressed in the ectoderm in a cluster of cells at the animal pole as well as in individual cells, possibly neurons, first on the ventral side then on the dorsal side, within the vegetal part of the *foxG* expression domain. At the prism/early pluteus stage, the pattern of *foxG* resolves into a thin belt of cells on the ventral side of the presumptive ciliary band (Figure 1A42) [34] while Delta expression now occurs in a salt and pepper pattern within the ciliary band and facial ectoderm (Figure 1A43) [55].

For simplification we divide the ectoderm into three main territories along the dorsal-ventral axis, however there are additional regional differences in gene expression that show that more than three regions can be defined (Figure 1C). For example, the homeobox gene *nk1* is expressed in the ventral-vegetal ectoderm in a region fated to become the ventral supra-anal ectoderm (Figure 1C1). Similarly, several dorsally expressed genes such as *msx*, *id*, *oasis*, *admp2* or *unc4* are strongly expressed in the dorsal-vegetal region fated to become the dorsal supra-anal ectoderm (Figure 1A22–25; 38; Figure 1D2,3). Thus, the ectoderm near the vegetal pole is divided into at least two sub domains along the D/V axis. Gene expression patterns also revealed that the ventral and dorsal ectodermal regions are progressively regionalized into different domains. This is best illustrated by the dynamics of *goosecoid* expression. *goosecoid* and *brachyury* are initially co-expressed within the ventral ectoderm (Figure 1A5,11), but during gastrulation, the expression domain of *goosecoid* is progressively cleared from the center of the ventral ectoderm (Figure 1C3). While *goosecoid* expression is progressively shifted at the periphery of the ventral ectoderm, forming a belt of cells abutting the ciliary band, *brachyury* and *foxA* remain expressed at the center of the ventral ectoderm, where the stomodeum will form (Figure 1C2). Similarly, analysis of gene expression within the dorsal ectoderm revealed the existence of nested patterns, with genes like *nk2.2*, *tbx2/3* and *dlx* (Figure 1D4, Figure 1A,9,20) being expressed in a broader domain than genes like *msx*, *wnt5* or *smad6* (Figure.1A22,29,39; 1D3,4 see also [26]) and genes like *irxA* being expressed in a sub domain of the dorsal ectoderm that excludes the dorsal apex (Figure 1D1). Finally, sub regions can also be recognized within the ciliary band territory starting at the early gastrula stage, with genes like *fgfA*, *vegf*, *pax2/5/8* and *sprouty* being expressed in the ventral lateral region (Figure 1A33,34; Figure 1D6 and data not shown) [48], [56], genes like *onecut*/*hnf6* or *gfi1* being expressed in the entire presumptive ciliary band territory (Figure 1C5; Figure 1D5), and genes like *foxG*, which in vertebrates is expressed in and required for specification of the ventral telencephalon [57], [58], being expressed in a ventral subdomain of the ciliary band (Figure 1A42).

Interestingly, several genes whose expression is later confined to the ciliary band are initially expressed much more broadly in the ectoderm (Figure 1E). This is particularly apparent for *glypican*5, *fgfA*, *univin*, and *wnt8*, which are expressed in a large belt of ectodermal cells at blastula stage and also for the neural marker *onecut/hnf6* which is first expressed ubiquitously, then in a broad ventro-lateral domain, and only later in the ciliary band (Figure 1E1–6) [26], [46]–[48]. This suggests that the expression of these ciliary band marker genes is initiated by broadly distributed transcription factors and later repressed on the ventral and/or dorsal sides by additional factors. As a first step to dissect the ectoderm gene regulatory network, we analyzed the regulation of these broadly expressed ciliary band genes. Since SoxB1 plays a critical role in ectoderm patterning in the sea urchin [59] and in the specification and maintenance of neural regions in vertebrates [60], we tested if SoxB1 is required for expression of ciliary band marker genes (Figure 1F). Injection of morpholinos against SoxB1 abrogated the expression of most markers of the neurogenic ectoderm of the ciliary band including *onecut*, *gfi1*, *foxG*, *egip*, *fgfA*, *pax2/5/8*, *univin*, *wnt8* and strongly affected the spatial expression of *dri* and *otx* [14] (Figure 1F1–20). This result supports the idea that transcription of at least a subset of ciliary band marker genes is initiated by broadly distributed transcription factors such as SoxB1 and later restricted to the ciliary band by zygotic factors induced by Nodal and/or BMP signaling.

### Nodal and BMP2/4 promote ventral and dorsal ectodermal fates and repress ciliary band gene expression

We next tested how Nodal and/or BMP2/4 regulate the expression of the 36 genes identified in the in situ screen. We focused on Nodal and BMP2/4 since previous studies showed that these two ligands are essential for specification and patterning of the ventral and dorsal territories. We first analyzed the effects of overexpressing *nodal* or *bmp2/4* on the expression of ectodermal markers. Embryos were injected with *nodal* or *bmp2/4* mRNA and the expression of the ventral, dorsal, or ciliary band markers was monitored at different stages. In most cases, results were confirmed by treatments with recombinant mouse Nodal or BMP4.

**Figure 2:**
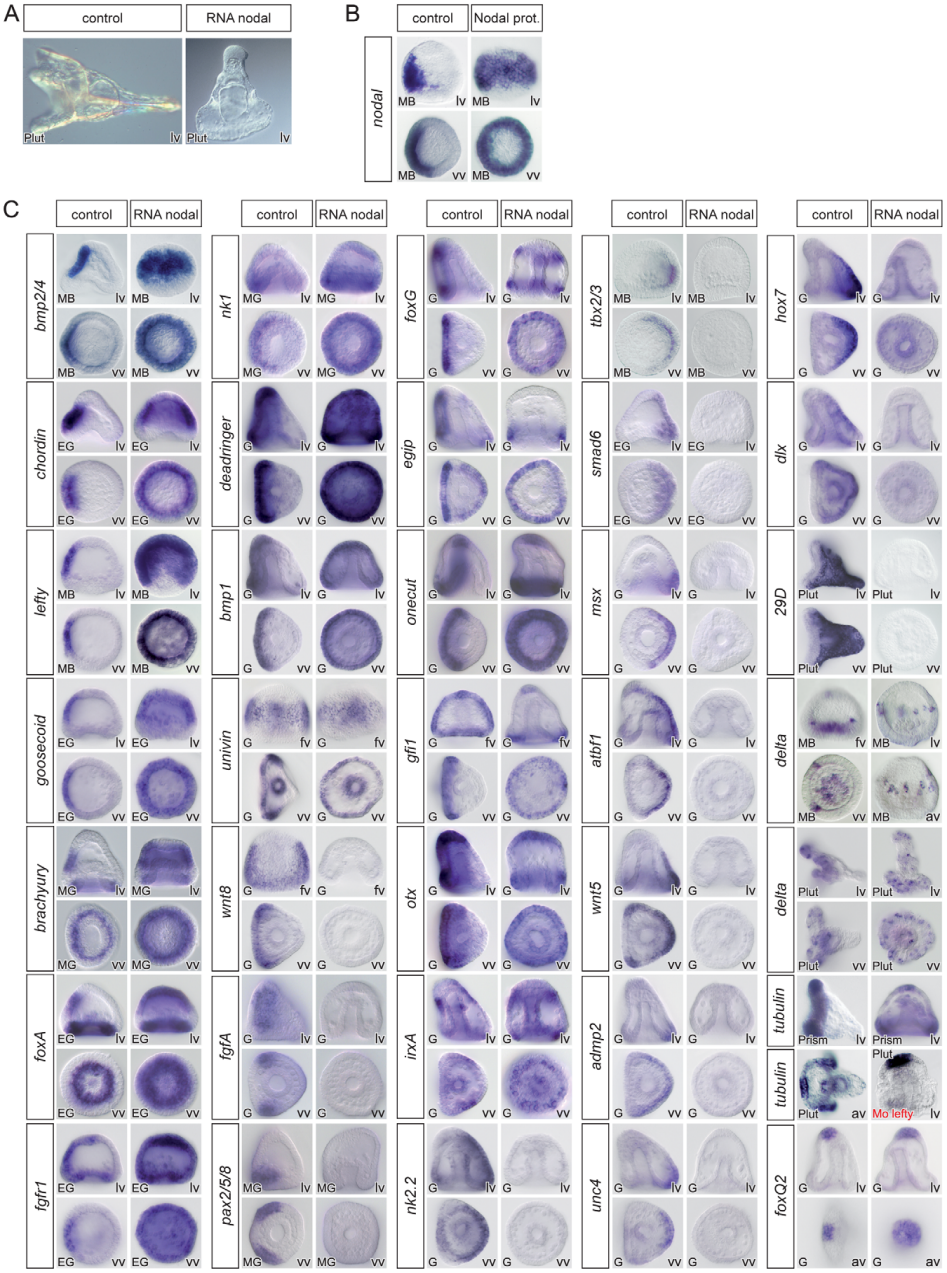
Overexpression of *nodal* represses the expression of ciliary band and dorsal marker genes and expands the expression of ventral markers genes. (A) morphology of *nodal* overexpressing embryos at 72h. (B) treatment with recombinant Nodal protein induces ectopic expression of *nodal* in a large belt of ectodermal cells. (C) Injection of *nodal* mRNA (200–400 µg/ml) caused the expression of most ventral marker genes to become radial. In most cases, however, the animal pole domain appeared to be resistant to ectopic expression of *nodal*. Overexpression of *nodal* also radialized the expression of *dri*, *bmp1* and *univin*, which are expressed in a broad domain encompassing the presumptive ventral ectoderm and ciliary band. In contrast, Nodal strongly antagonized the expression of ciliary band marker genes such as *wnt8*, *fgfA*, *pax2/5/8*, *foxG*, *egip*, *onecut*, *gfi1*, *otx*, *tubulinß3* or *Delta* in the equatorial region. Nodal overexpression efficiently repressed the expression of all the genes expressed in the dorsal ectoderm but did not affect the expression of marker genes of the animal pole (*tubulinß3*, *foxQ2*, *Delta*). Note that, starting at gastrula stage, *irxA* is expressed in a patch of animal-ventral cells that likely corresponds to the upper part of the presumptive stomodeum. Therefore in *nodal* overexpressing embryos, this territory becomes radial and forms a belt of cells near the animal pole. Also note that while a ciliary band does form in the vegetal region of *nodal* overexpressing embryos, as shown by the expression of ciliary band genes around the blastopore, no ciliary band forms in the vegetal region of *lefty* morphants as indicated by the absence of *tubulinß3* expression. In contrast, *nodal* overexpression does not affect the expression of animal pole marker genes such as *foxQ2*. lv, lateral view, vv, vegetal pole view, av, animal pole view, fv, frontal view.

Overexpression of *nodal* mRNA or treatments with recombinant Nodal protein dramatically expanded the expression of *nodal*, *bmp2*/*4*, *chordin*, *lefty*, *goosecoid* and *brachyury* as reported previously (Figure 2) [13], [26]. Overexpression of Nodal also expanded the ectodermal domain of expression of *foxA* and *fgfr1* at mesenchyme blastula stages. Similarly, the expression domain of *nk1*, which is normally restricted to the ventral vegetal ectoderm, became radial in *nodal* overexpressing embryos. Genes expressed in the ciliary band behaved differently depending on the gene. In the case of *deadringer*, *bmp1* and *univin*, which are expressed in the ciliary band and in the ventral ectoderm, overexpression of *nodal* expanded their expression to the whole ectoderm. In the case of *wnt8*, which is expressed in two broad lateral stripes at gastrula stages, as well as in the case of *fgfA* and its downstream target *pax2/5/8*, which are expressed in the ventral sub domain of the ciliary band, all expression was eliminated by exogenous *nodal*. However, in the case of *foxG*, *egip*, *onecut/hnf6*, *gfi1*, *otx*, exogenous *nodal* suppressed expression in most of the ectoderm except in the animal and/or vegetal most domains of the ectoderm. Overexpression of *nodal* increased the number of ventral-vegetal cells that normally express *Delta* at the early gastrula stage and, at 48h, produced ventralized embryos in which most Delta expressing cells were located at the animal pole and in the vegetal most ectoderm. Largely similar phenotypes were obtained following treatments with nickel chloride (Figure S3) although we noted intriguing differences in the behavior of a few genes including *wnt8*, *univin*, *fgfA* and *pax2/5/8*, in response to these perturbations. Overall, these data are consistent with the idea that in *nodal*-overexpressing or nickel treated embryos, radially expressed Nodal promotes specification of ventral ectodermal fates and suppresses specification of the ciliary band in a large equatorial region but not in the animal pole region or in the ectoderm surrounding the blastopore. One likely reason that may explain why the vegetal ectoderm is refractory to Nodal overexpression or to nickel treatment is that in these embryos, Nodal signaling is restricted to the equatorial region [13]. The vegetal ectoderm may therefore be protected from Nodal activity by Lefty which is thought to diffuse farther than Nodal [12], . Consistent with this idea, in Nodal treated embryos and in nickel treated embryos, *nodal* expression expands to a large belt of cells in the equator and a ciliary band differentiates in the vegetal most ectoderm while in *lefty* morphants, which also display unrestricted Nodal signaling, ciliary band marker genes such as *tubulinß3* and *onecut/hnf6* are expressed in the animal pole region but not in the vegetal ectoderm (Figure 2) [12]. Taken together, these results suggest that a Lefty dependent inhibition of Nodal signaling is required for ciliary band formation in the vegetal pole region. Finally, as expected, overexpression of *nodal* eliminated the expression of all the dorsal marker genes we tested including, *nk2.2*, *tbx2/3*, *smad6*, *msx*, *atbf1*, *wnt5*, *admp2*, *unc4*, *hox7*, *dlx*, and *29D* (Figure 2).

**Figure 3:**
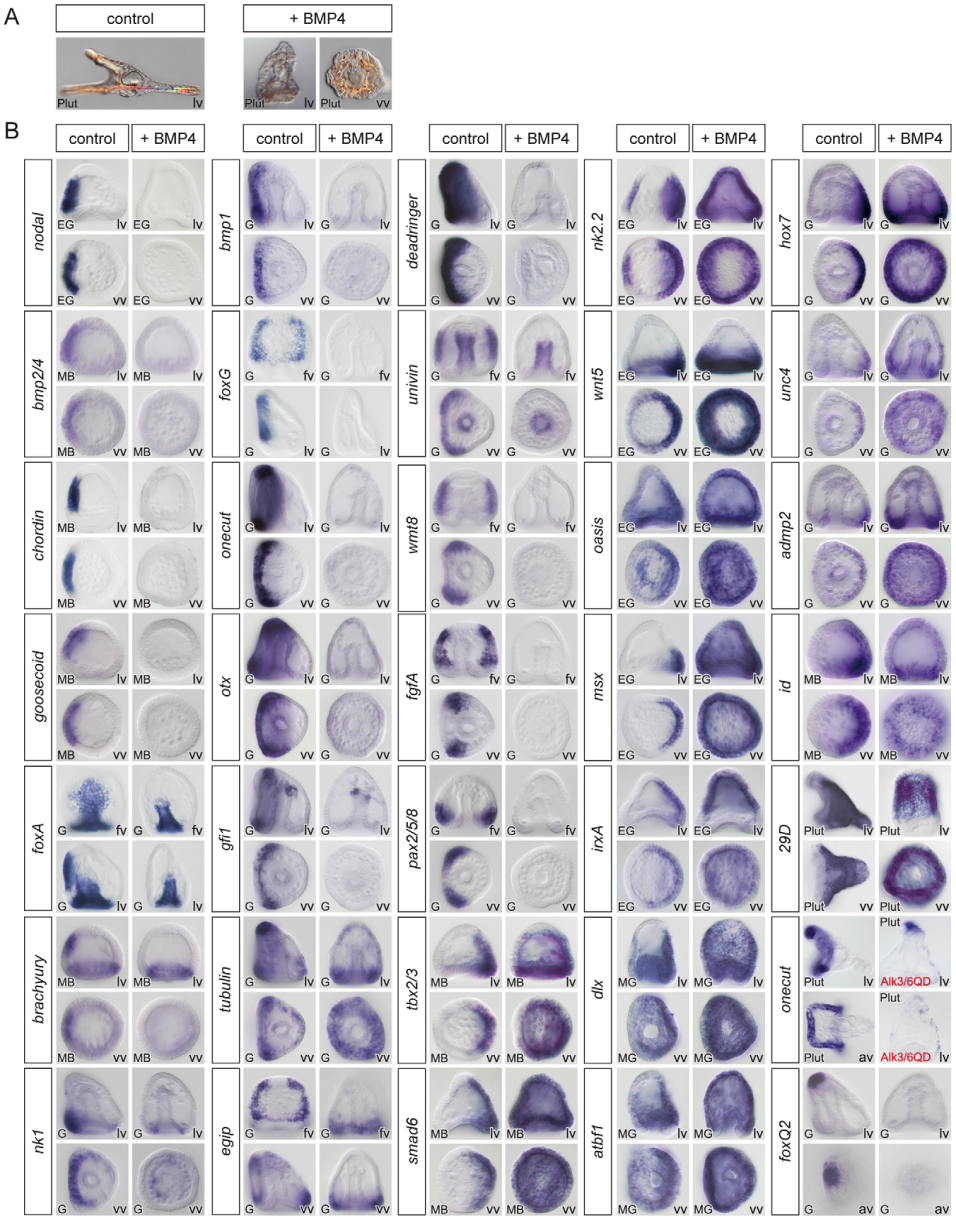
Overactivation of BMP signaling eliminates the expression of ventral and ciliary band marker genes and expands the dorsal territory. (A) Morphology of BMP2/4 overexpressing or BMP4 treated embryos at 72h. Embryos with overactivated BMP signaling are elongated and covered with a thin and squamous ectoderm and possess ectopic spicules. (B) Expression of ventral, dorsal and ciliary band marker genes in embryos misexpressing BMP2/4. The results presented were obtained using treatments with recombinant BMP4 protein except one experiment in which an activated BMP receptor (Alk3/6QD) [26] was used. For all these genes, identical results were obtained by overexpression of BMP2/4 mRNA. Overactivation of BMP2/4 signaling eliminated the expression of ventral markers and of ciliary band genes such as *bmp1*, *foxG*, *onecut/hnf6*, *otx*, *gfi1*, *tubulinß3*, *egip*, *deadringer*, *univin*, *fgfA*, and *pax2/5/8*. Overactivation of BMP signaling dramatically expanded the expression of all the dorsal marker genes and eliminated the expression of markers of the animal pole domain such as *foxQ2*. lv, lateral view, vv, vegetal pole view, av, animal pole view, fv, frontal view.

Reciprocally, overexpression of *bmp2/4* or treatments with recombinant BMP4 protein eliminated expression of all the ventral marker genes we tested including *nodal*, *bmp2/4*, *chordin*, *goosecoid*, *foxA*, *lefty* (not shown), *brachyury*, and *nk1* (Figure 3). As in the case of *nodal* overexpression, misexpression of *bmp2/4* or of the activated Alk3/6 BMP receptor (Alk3/6QD) [26] strongly suppressed the expression of the ciliary band markers such as *bmp1*, *foxG*, *onecut/hnf6*, *otx*, *gfi1*, *tubulinß3*, *egip*, *dri*, *univin*, *wnt8*, *fgfA* and *pax2/5/8*. However, unlike in the case of *nodal* overexpressing or nickel treated embryos, which conserved expression of ciliary band markers in the animal pole and in vegetal ectodermal regions, overexpression of *bmp2/4* or of the activated type I BMP receptor (Alk3/6QD) efficiently eliminated the expression of all the ciliary band markers at the animal pole and in the vegetal most ectoderm as well as the expression of animal pole specific markers such as *foxQ2* highlighting the very strong antagonism existing between high level of BMP2/4 signaling and specification of the animal pole and ciliary band cell fates. Finally, misexpression of BMP2/4 dramatically expanded the expression of all the dorsal marker genes including *tbx2/3*, *smad6*, *nk2.2*, *wnt5*, *oasis*, *msx*, *irxA*, *dlx*, *atbf1*, *hox7*, *unc4*, *admp2*, *id* and *29D*.

### Discrimination between direct versus indirect targets of Nodal and BMP2/4

**Figure 4:**
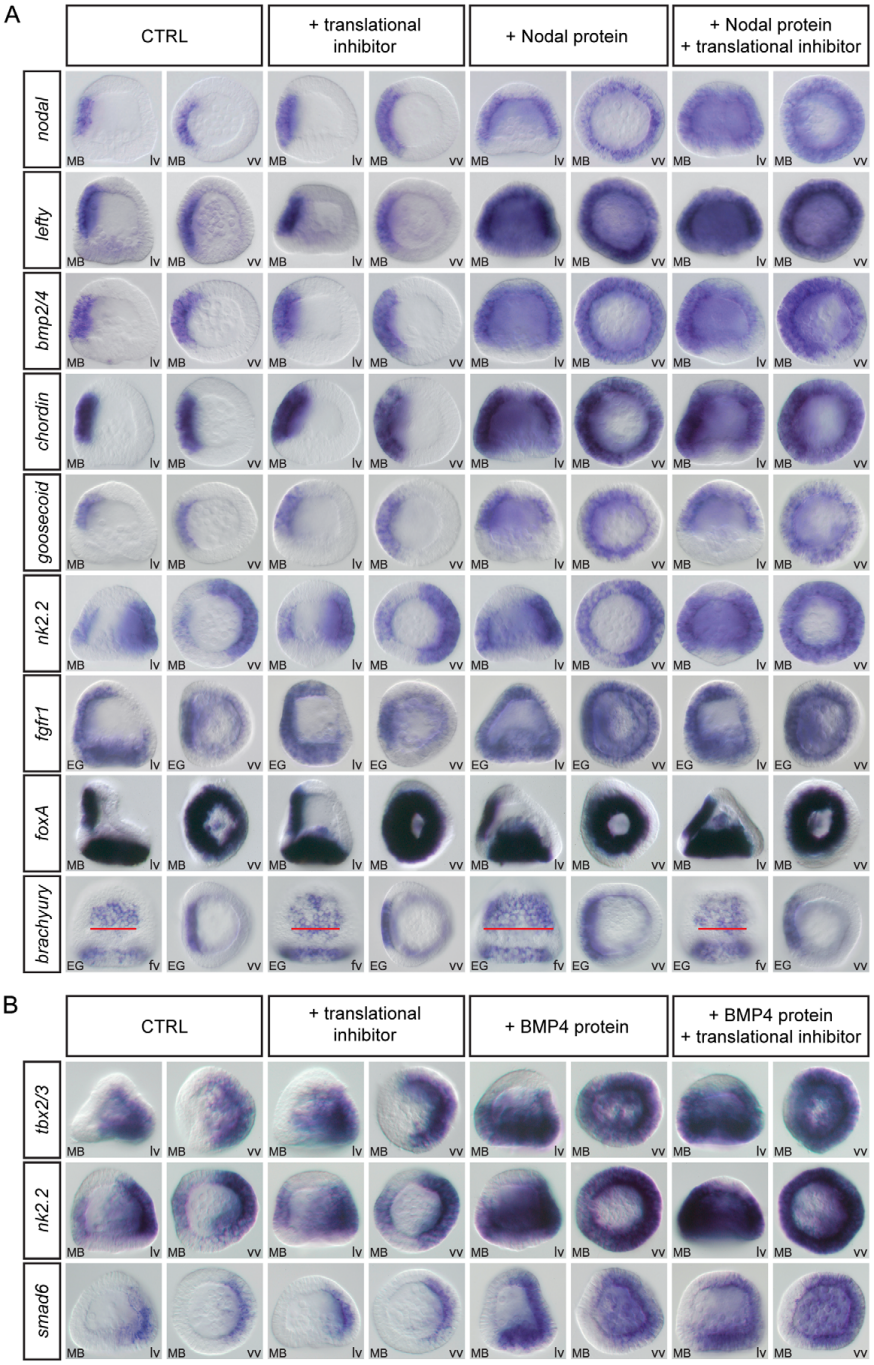
*nodal*, *lefty*, *bmp2/4*, *chordin*, *goosecoid*, *nk2.2*, and *fgfr1* are direct targets of Nodal while *tbx2/3*, *nk2.2*, and *smad6* are direct targets of BMP2/4 signaling. (A,B) Embryos at the late blastula stage were treated for two hours with recombinant Nodal or BMP4 protein in the presence or absence of puromycin. At the end of the treatment the embryos were fixed and the expression of the indicated genes was analyzed by in situ hybridization. In control experiments, DMSO or the translational inhibitor were added alone. (A) A short treatment with recombinant Nodal protein induced strong ectopic expression of early expressed genes such as *nodal*, *lefty*, *bmp2/4*, *chordin*, *goosecoid*, *nk2.2* and *fgfr1*. This ectopic expression was still observed in the presence of protein synthesis inhibitor indicating that these genes are early targets of Nodal signaling. In contrast, this 2h treatment with Nodal did not induce ectopic expression of genes expressed later such as *foxA* and only induced a partial expansion of *brachyury* expression, an effect that disappeared in the presence of the protein synthesis inhibitor. These genes are therefore likely secondary targets of Nodal signaling. Note that in control embryos the width of the *brachyury* expression territory encompasses 8–9 cells (red bar) while in Nodal treated embryos the width of this stomodeal field increases to about 12–15 cells. In embryos treated with Nodal and the translation inhibitor, the width of the stomodeal field is similar to that in control embryos. (B) Similarly, BMP4 treatment induced massive ectopic expression of the early expressed genes *tbx2/3*, *nk2.2* and *smad6* even in the presence of a translational inhibitor but failed to induce ectopic expression of dorsal markers genes expressed later. *tbx2/3*, *nk2.2* and *smad6* are therefore likely direct targets of BMP2/4 signaling while the other dorsally expressed genes are likely indirect targets whose expression requires protein synthesis downstream of activation of the BMP receptors. Identical results were obtained by using emetine as translational inhibitor. lv, lateral view, vv, vegetal pole view fv, frontal view.

We next sought to determine which genes are direct targets of Nodal and BMP2/4 signaling. Based on the timing of expression of the ventral or dorsal markers genes, it was expected that only a subset would be direct targets of Nodal or BMP2/4 signaling. For example, only *lefty*, *bmp2/4*, *chordin*, *goosecoid*, *nk2.2*, *fgfr1* and *tbx2/3* are expressed at swimming blastula stage, the expression of most of the other starting only at mesenchyme blastula stage. We therefore tested whether the ventral marker genes are transcribed in direct response to Nodal and whether the dorsal marker genes are transcribed in direct response to BMP2/4 signaling or if transcription of these genes requires protein synthesis. To achieve this, we treated embryos at the hatching blastula, mesenchyme blastula or gastrula stages with recombinant mouse Nodal or BMP2/4 proteins in the presence or absence of a protein synthesis inhibitor (Figure 4), and analyzed the expression of all the ventral and all the dorsal marker genes.

Short treatments with recombinant Nodal protein at blastula stage strongly induced expression of *nodal*, *lefty*, *bmp2/4*, *chordin*, *goosecoid*, *nk2.2* and *fgfr1* throughout most of the ectoderm (Figure 4A). These effects were observed even in the presence of a translational inhibitor suggesting that these genes are direct targets of Nodal signaling. In contrast, short treatments with Nodal at either mesenchyme blastula or gastrula stages failed to induce any ectopic expression of the other ventral genes such as *foxA* (Figure 4A) *foxG*, *nk1*, or *deadringer* (data not shown), which are expressed in the ectoderm starting at or after mesenchyme blastula. This suggests that these genes are indirect targets of Nodal signaling that cannot be induced during the short interval of the treatment. Interestingly, in the case of *brachyury*, a weak but consistent broadening of the ectodermal domain of expression was observed following treatment with Nodal. However, this effect was abolished by treatment with the protein synthesis inhibitor, consistent with this gene being an indirect target of Nodal signaling. Similarly, among all the dorsal marker genes we tested, 3 genes were strongly induced by treatments with BMP2/4, even in the presence of protein synthesis inhibitors. These were *tbx2/3*, *nk2.2* and *smad6* (Figure 4B). Short treatments with high doses of BMP2/4 failed to induce expression of *irxA*, *dlx*, *msx*, *atbf1*, *hox7*, *id*, *unc4*, *oasis*, *wnt5*, *admp*2 or *glypican 5* (data not shown) suggesting that these genes may be indirect targets of BMP signaling. The very good correlation between the results of this induction assay and the timing of expression of the downstream targets of Nodal and BMP2/4 indicates that this assay predicts with good confidence the direct, and probably also the indirect, target genes of these ligands at swimming blastula stage. It should be kept in mind however, that at later stages, this assay does not allow to rule-out completely the existence of a direct input from Nodal or BMP2/4 to downstream target genes. An alternative explanation for the fact that several genes appear to be refractory to induction by recombinant Nodal or BMP4 proteins is that after swimming blastula stage, the ventral and dorsal ectoderm may no longer be competent to switch their gene regulatory networks to a state that supports expression of dorsal or ventral genes respectively.

### Nodal and BMP2/4 dependence of ectodermal gene expression

**Figure 5:**
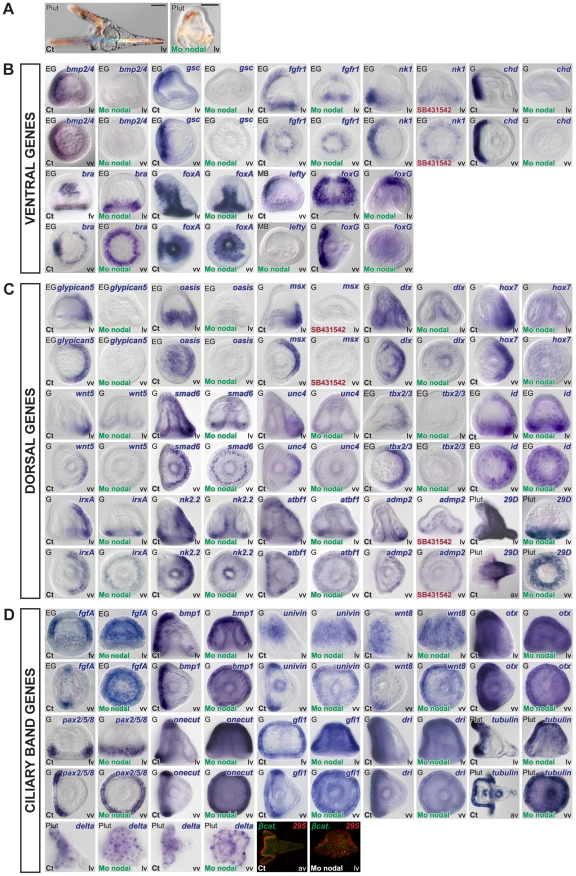
Blocking Nodal function prevents expression of ventral and dorsal marker genes in the presumptive ectoderm and causes massive ectopic expression of ciliary band genes. (A) morphology of *nodal* morphants at 72h. Note that most of the ectoderm of these embryos differentiates into a thick ciliated ectoderm that resembles the ciliary band ectoderm. (B–D) Embryos were injected with a Nodal morpholino or treated with the Nodal receptor inhibitor SB431542 and the expression of ventral, dorsal or ciliary band genes was analyzed at the relevant stages. (B,C) All the ventral and all the dorsal marker genes tested required Nodal to be expressed in the presumptive ventral and presumptive dorsal ectoderm respectively. However, in the *nodal* morphants, a number of genes expressed dorsally continued to be expressed in the ectoderm derived from the vegetal region and/or in the mesendoderm including *tbx2/3*, *id*, *irxA*, *nk2.2*, *atbf1*, *admp2* and 29D (the residual expression of *tbx2/3* is not visible here since it is mostly visible at gastrula stages). This indicates that in the *nodal* morphants there is a residual D/V polarity with the vegetal most ectoderm adopting a dorsal identity. (D) Inhibition of Nodal signaling caused a massive ectopic expression of ciliary band genes throughout the ectoderm. Note that genes expressed throughout the ciliary band territory such as *bmp1*, *otx*, *onecut*, *gfi1*, *dri*, or *tubulinß3* are ectopically expressed throughout the ectoderm of Nodal morphants. Genes that are expressed in sub domains of the ciliary band such as *pax2/5/8*, *fgfA*, *univin* or *wnt8* are also ectopically expressed and display a radial expression but in accordance with their normal animal-vegetal boundaries. The neural marker *Delta* and the ciliary band antigen 295, which in control embryos labels the ciliated cuboidal cells of the ciliary band, are also expressed ectopically throughout the thick ciliated ectoderm typical of *nodal* morphants. lv, lateral view, vv, vegetal pole view, fv, frontal view. Scale bar: 100µm.

We next attempted to determine if the activity of Nodal and BMP2/4 accounts for the restricted expression of all of the ventral and all the dorsal genes. Embryos were injected with a *nodal* morpholino and the expression of ventral, dorsal or ciliary band markers analyzed at successive stages (Figure 5). Expression of all the ventral marker genes that we tested including, *bmp2/4*, *goosecoid*, *fgfr1*, *nk1*, *chordin*, *brachyury*, *foxA* and *lefty* disappeared in the Nodal morphants, consistent with previous results (Figure 5B) [13], [29], [38]. Injection of the *nodal* morpholino also largely prevented expression of *foxG*, confirming that this gene is induced downstream of Nodal signaling [29]. We also found that in Nodal morphants, the expression of all dorsal markers genes was strongly downregulated in most of the ectoderm, with responses falling into two categories: for some genes, e.g. *glypican5*, *oasis*, *msx*, dlx, *hox7*, *wnt5*, *smad6*, or *unc4*, expression completely disappeared in the Nodal morphants (Figure 5C). Others, e.g. *tbx2/3*, *id*, *irxA*, *nk2.2*, *atbf1*, *admp2* and 29D displayed residual expression in the vegetal-most ectoderm and/or in the PMCs indicating Nodal-independent expression of these genes in the presumptive dorsal vegetal ectoderm.

A striking result was obtained when we analyzed the expression of ciliary band markers in the *nodal* morphants (Figure 5D). The expression of most ciliary band markers dramatically expanded to most of the ectoderm following inhibition of Nodal signaling. This was the case for *fgfA*, *bmp1*, *univin*, *wnt8*, *otx*, *pax2/5/8*, *onecut/hnf6*, *gfi1*, *dri*, as well as of the late ciliary band marker *tubulinß3* and the ciliary band antigen *295*. Importantly, expression of Delta, which at pluteus stages identifies individual neurons of the facial ectoderm and ciliary band region [26], [55], was expanded to the whole ectoderm in Nodal morphants, strongly suggesting that most of the ectoderm is converted into neurogenic ectoderm in these embryos. Largely similar results were obtained using a pharmacological inhibitor of the Nodal receptor [62] (Figure S4). Taken together, these results show that Nodal signaling is essential for expression of all the ventral and of all the dorsal marker genes within the ectoderm. In the absence of Nodal, expression of all the ventral and dorsal marker genes is abolished and ciliary band genes are ectopically expressed throughout most of the ectoderm.

**Figure 6 pgen-1001259-g006:**
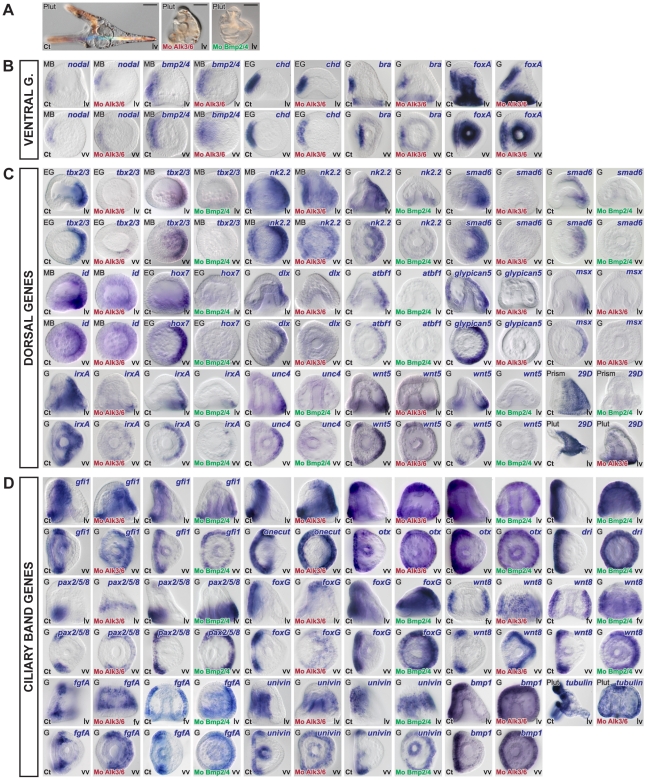
Blocking BMP2/4 or Alk3/6 signaling strongly downregulates the expression of dorsal genes and causes massive ectopic expression of ciliary band marker genes on the dorsal side. (A) Morphology of alk3/6 and BMP2/4 morphants at 72h. Ventral structures such as the stomodeum do form in embryos injected with the alk3/6 or BMP2/4 morpholinos but dorsal structures such as the apex fail to differentiate. (B–D) Embryos were injected with BMP2/4 or Alk3/6 morpholinos and the expression of ventral, dorsal or ciliary band marker genes was analyzed at the relevant stages. (B) Inhibition of BMP2/4 signaling does not interfere with the expression of ventral genes. (C) The expression of all the dorsal marker genes is strongly reduced or abolished following inhibition of BMP2/4 or Alk3/6 function. A residual expression in the vegetal most region is still observed for certain genes such as *irxA*, *wnt5*, *id*. (D) Inhibition of Alk3/6 or BMP2/4 causes a striking ectopic expression of ciliary band genes throughout the dorsal ectoderm. lv, lateral view, vv, vegetal pole view, av, animal pole view, fv, frontal view. Scale bar: 100µm.

We also examined the effect of knocking down BMP signaling on the expression of the ventral, dorsal and ciliary band markers (Figure 6). As expected, we found that expression of all the ventral markers that we tested was independent of BMP2/4 signaling: *nodal*, *bmp2/4*, *chordin*, *brachyury* or *foxA* were expressed at similar levels and in similar domains in the controls and in the *alk3/6* morphants (Figure 6B). Removing BMP2/4 or Alk3/6 function affected the expression of dorsal marker genes in a way very similar to that caused by removing Nodal: expression of most genes including *wnt5*, *atbf1*, *hox7*, *msx*, *dlx*, *smad6*, *tbx2/3*, *unc4* was abolished while for *irxA*, *nk2.2* and *id*, residual expression was still observed in the vegetal most ectoderm on the presumptive dorsal side (Figure 6C). These results confirm that expression of all the dorsal ectodermal genes stringently relies on BMP2/4 signaling and that in the absence of Nodal or BMP2/4 signals, no other signals compensate for the lack of these inducers. Again, a striking result was observed when we analyzed the expression of ciliary band markers in the *bmp2/4* or Alk3/6 morphants. For all of them, including *gfi1*, *onecut/hnf6*, *otx*, *deadringer*, *pax2/5/8*, *foxG*, *wnt8*, *fgfA*, *univin*, *bmp1* and *tubulinß3*, loss of BMP2/4 signaling caused a dramatic ectopic expression in the dorsal ectoderm (Figure 6D). This ectopic expression transformed the normally bilateral expression domains of *fgfA*, *pax2/5/8*, *foxG*, *gfi1*, *univin*, and *wnt8* into a horseshoe shaped domain covering the lateral and dorsal regions and caused the expression domain of *deadringer* and *otx* to become radial. These results reveal that in addition to promoting specification of dorsal cell fates, an essential function of BMP2/4 signaling is to repress ciliary band gene expression within the dorsal ectoderm.

### Construction of a provisional gene regulatory network

To establish the functional hierarchy between key ventral, dorsal and ciliary band genes, we designed morpholinos against 17 transcription factors and 8 signaling molecules expressed within the ectoderm with a restricted pattern along the dorsal-ventral axis. Among these 25 morpholinos, 19 (*alk4/5/7, alk3/6, brachyury, bmp2/4, chordin, foxA, foxG, fgfA, goosecoid, irxA, lefty, tbx2/3, dlx, msx, nodal, onecut/hnf6, soxB1, univin, wnt8*) gave a clearly recognizable morphological phenotype (Figure 5–9). The expression of 15 transcription factors (*goosecoid, brachyury, foxA, nk1, nk2.2, tbx2/3, msx, smad6, hox7, irxA, onecut, gfi1, dri, pax2/5/8, foxG*) and 8 signaling factors (*nodal, bmp2/4, fgfA, chordin, wnt8, univin, wnt5, glypican5*) was analyzed at different stages in the 17 morphant backgrounds while in the case of *nodal* and *bmp2/4* morphants we analyzed the expression of an additional set of 17 marker genes (Tables S1, S2). In addition, we overexpressed a subset of genes encoding transcription factors (*goosecoid*, *foxA*, *foxG*, *deadringer*, *nk2.2*, *tbx2/3*, *msx*, *smad6*) and signaling molecules (*nodal*, *bmp2/4*, *chordin*) and analyzed the expression of ventral, dorsal and ciliary band genes in these embryos. Since many of the genes identified in our screens including *brachyury*, *foxA*, *otx*, *smad6*, *tbx2/3*, *wnt5*, *oasis*, *univin*, *wnt8*, *rkhd*, *ptb*, *fgfA*, *Delta* are expressed not only in the ectoderm but also in the mesendoderm and since many other markers such as *atbf1*, *irxA*, *nk2.2 or egip*, *oasis*, *wnt5*, *glypican5*, *wnt8*, *Delta*, *otx* or *bmp1* are expressed in more than one region and sometimes in both the ventral and dorsal ectoderm, we used in situ hybridization rather than QPCR to monitor the consequences of the perturbations. In situ hybridization is usually not used as the primary technique in large-scale projects such as gene regulatory network analysis since it is time and effort consuming and requires large numbers of injected embryos. However, we believe it is the only technique that provides the necessary spatial resolution to accurately analyze the expression of genes with complex expression patterns in perturbed embryos. Furthermore, when used with appropriate controls, in situ hybridization can provide a good estimate of the level of expression in perturbed embryos compared to controls. To provide a temporal view of the consequences of these perturbations and avoid secondary effects, the expression of the genes analyzed in response to *nodal* or *bmp2/4* overexpression was examined at two different stages, soon after the onset of their restricted expression, and at a later stage, most often early or late gastrula stage depending on the gene analyzed.

**Figure 7 pgen-1001259-g007:**
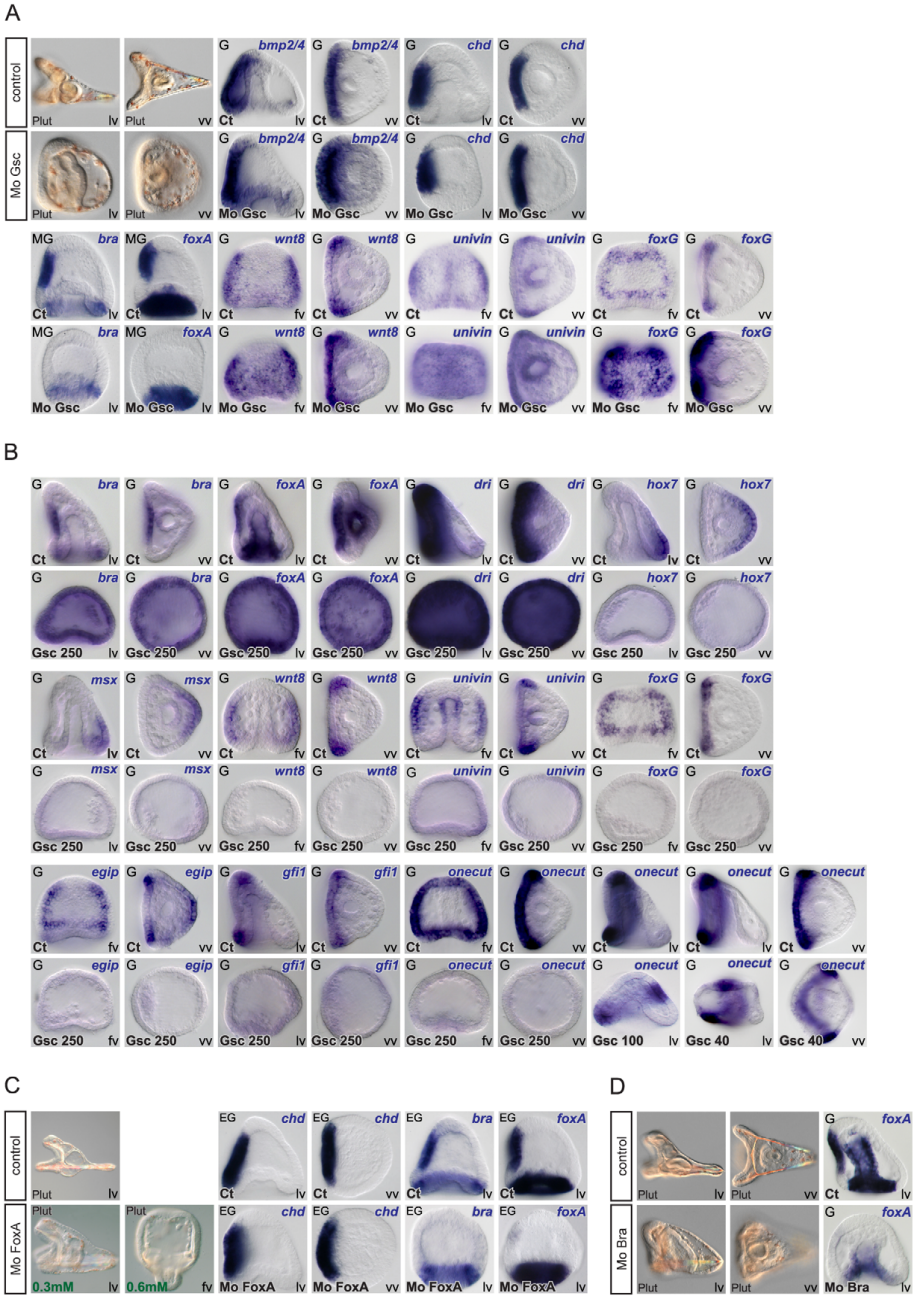
Epistasis analysis of ventral genes: goosecoid as a key regulator of *brachyury* and *foxA* expression and a repressor of ciliary band genes. (A) Morphology of goosecoid morphants at 48h. Interfering with *goosecoid* function strongly delays gastrulation and produces partially radialized embryos as reported previously [35]. *bmp2/4* and *chordin* are expressed normally in these embryos but expression of *brachyury* and *foxA* is lost while *wnt8*, *univin* and *foxG* and are ectopically expressed within the ventral ectoderm. (B) Injection of *goosecoid* mRNA at 250 µg/ml produced radialized embryos as reported previously [35]. Overexpression of *goosecoid* caused ectopic expression of *brachyury*, *foxA* and *deadringer* throughout the ectoderm. Overexpression of *goosecoid* also led to repression of dorsal markers genes such as *hox7* and *msx* and of ciliary band marker gene expression as shown for *wnt8*, *univin*, *foxG*, *egip*, *gfi1* and *onecut/hnf6*. Note the dose dependent repression of *onecut/hnf6*, with low doses (40–100 µg/ml) causing a dorsal shift of the expression domain of *onecut/hnf6* and high doses (250 µg/ml) leading to complete repression. (C) Embryos injected with low doses (0.3 mM) of the *foxA* morpholino lacked a stomodeum as reported previously. At higher doses (0.6 mM), the *foxA* morpholino strongly interfered with gastrulation resulting in embryos with no gut or a small exogastrulated gut. *foxA* morphants had a normal expression of *chordin* but lacked expression of *brachyury* and *foxA*. (D) *brachyury* morphants, like *foxA* morphants, lacked a stomodeum and did not express *foxA* in the presumptive stomodeal region. lv, lateral view, vv, vegetal pole view, fv, frontal view.

**Figure 8 pgen-1001259-g008:**
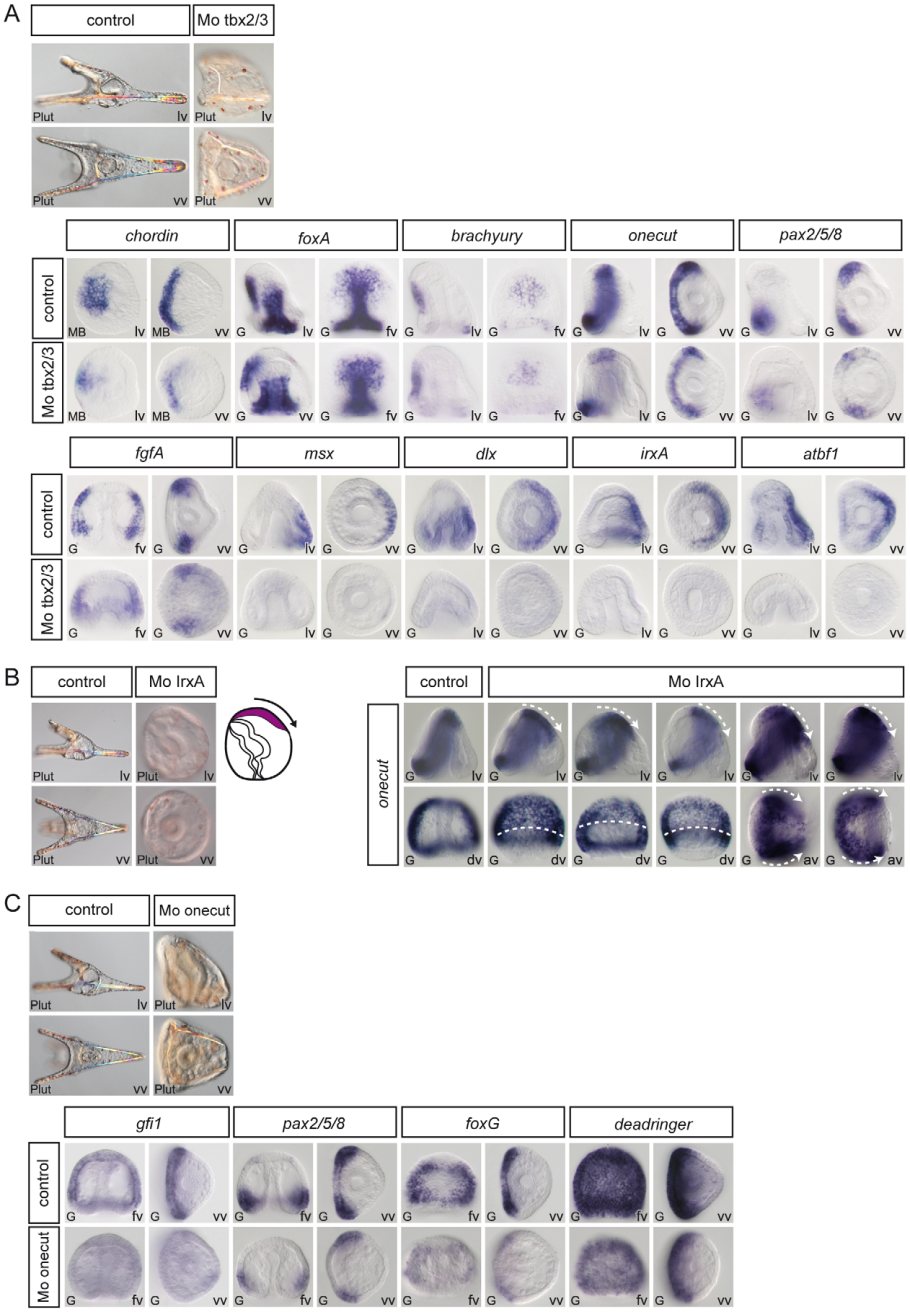
Epistasis analysis of dorsal genes: *irxA* as a repressor of ciliary band gene expression downstream of *tbx2/3*. (A) *tbx2/3* morphants are partially radialized and do not elongate along the D/V axis. The expression of ventral genes such as *chordin*, *foxA*, *brachyury*, *onecut/hnf6* and of ciliary band genes such as *pax2/5/8* and *fgfA* is largely normal in these embryos. In contrast, expression of dorsal marker genes such as *msx*, *dlx*, *irxA* and *atbf1* is lost at gastrula stages. (B) *irxA* morphants are partially radialized and occasionally show an ectopic ciliary band like thickened region within the dorsal region. Expression of *onecut/hnf6* is dramatically expanded towards the dorsal side of i*rxA* morphants. (C) *Onecut/hnf6* morphants are slightly radialized but do form a morphologically recognizable ciliary band. In these embryos, the expression of ciliary band genes *gfi1* is absent while the expression of *pax2/5/8*, *foxG* or *deadringer* is strongly reduced. lv, lateral view, vv, vegetal pole view, av, animal pole view, dv, dorsal view, fv, frontal view.

**Figure 9:**
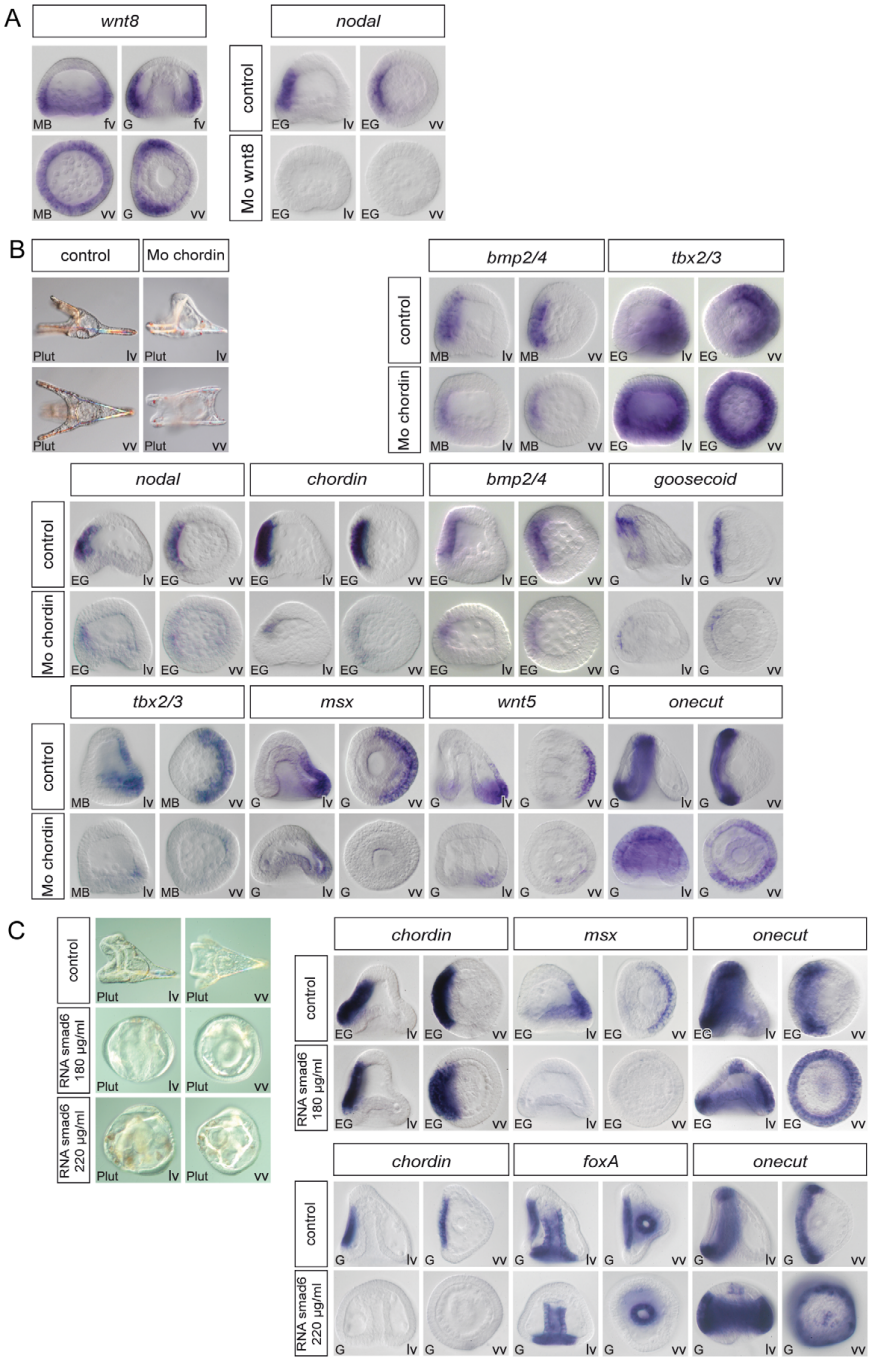
Regulation of D/V patterning by extracellular and intracellular modulators of Nodal and BMP signaling. (A) Wnt8 regulates maintenance of *nodal* expression. At mesenchyme blastula stage, *wnt8* starts to be expressed in a large belt of ectodermal cells within the vegetal hemisphere. At gastrula stage *wnt8* expression is detected in the animal and vegetal hemispheres in two broad lateral stripes that flank the ventral ectoderm. In *wnt8* morphants, expression of *nodal* is lost at mesenchyme blastula stage. (B) Chordin is essential for normal patterning along the D/V axis and plays a key role in restricting the expression of ciliary band genes. In *chordin* morphants, most of the ectoderm derived from the animal hemisphere differentiates into a ciliary band-like ectoderm. Expression of ventral (*nodal*, *chordin*, *bmp2/4*, *goosecoid*) and dorsal (*msx*, *wnt5*) marker genes is strongly downregulated in these embryos. A transient ectopic expression of *tbx2/3* is detected at early mesenchyme blastula stage in these embryos followed by downregulation of the gene. Restriction of the expression of the ciliary band gene *onecut/hnf6* is disrupted in *chordin* morphants and ectopic expression of this gene is detected in the ectoderm. (C) At high concentration (>220 µg/ml) *smad6* mRNA suppressed the ectodermal expression of Nodal targets genes such as *chordin* and *foxA* and caused a dramatic ectopic expression of *onecut/hnf6* throughout the ectoderm. At lower doses (<180 µg/ml), *smad6* mRNA did not interfere with the expression of *chordin* but specifically antagonized with the expression of BMP target genes as *msx* and caused the ectopic expression of *onecut/hnf6* in the dorsal ectoderm. lv, lateral view, vv, vegetal pole view, fv, frontal view.

Information derived from these perturbations analyses was combined with earlier results to build a provisional gene regulatory network. The main features of this gene regulatory network are described below.

### Goosecoid is required to initiate a stomodeal regulatory sub circuit and to repress ciliary band genes

Low levels of *goosecoid* transcripts are present maternally then their abundance increases sharply at swimming blastula stage, shortly after the peak of *Nodal* expression [35] (Figure S2). Expression of *lefty*, *chordin*, *bmp2/4*, *fgfr1* and *goosecoid*, was unchanged in the *goosecoid* morphants consistent with these genes being direct targets of Nodal signaling and with previous studies [38] (Figure 7A and data not shown). Interestingly, at gastrula stages, strong ectopic expression of *wnt8*, *univin* and *foxG* was detected in the ventral ectoderm of *goosecoid* morphants indicating that one function of Goosecoid is to repress expression of these three genes in the ventral ectoderm between blastula and gastrula stages. In contrast, ectodermal expression of *foxA* and *brachyury*, two likely indirect targets of Nodal required for mouth formation, was lost in the *goosecoid* morphants, consistent with the lack of stomodeum in these embryos (Figure 7A) [42]. Reciprocally, overexpression of *goosecoid* caused a dramatic expansion of *foxA* and *brachyury* (Figure 7B). Therefore, in the sea urchin as in vertebrates, *brachyury* and *foxA* are targets of Nodal signaling but unlike in vertebrates, in the sea urchin, they are not primary targets of Nodal since their expression depends on the zygotic expression of *goosecoid* [63]–[65]. Overexpression of *goosecoid* also expanded the expression of *deadringer* as reported previously by Bradham et al. [22], [66]. In contrast, the two dorsal marker genes *hox7* and *msx* failed to be expressed in the *goosecoid* overexpressing embryos consistent with previous studies showing that *goosecoid* overexpression suppresses expression of dorsal genes such as *tbx2/3* and *spec1* [35], [40]. Overexpression of *goosecoid* also abolished the expression of all the other ciliary band genes that we tested including *wnt8*, *univin*, *foxG*, *egip*, *gfi1* and *onecut/hnf6*. Taken together these observations suggest that *goosecoid* plays a double function, first by allowing expression of stomodeal genes such as *foxA* and *brachyury* and second by suppressing the expression of ciliary band and dorsal genes.

Once *goosecoid* and *foxA* have been turned on, Brachyury and FoxA cross regulate each other so that *brachyury* maintains *foxA* expression while *foxA* promotes *brachyury* expression (Figure 7C, 7D). When the function of either of the two genes was blocked with a morpholino, expression of the other gene was lost and the resulting embryos developed without a stomodeum. The role of these cross regulatory interactions between *brachyury* and *foxA* may be to stabilize and lock the specification of the ventral ectoderm that has been initiated by Nodal as described in the endomesoderm GRN, for example between the transcription factors *hex* and *tgif* [7].

### Tbx2/3: an early regulator of dorsal gene expression downstream of BMP2/4 signaling

Inhibition of *tbx2/3* function strongly perturbed establishment of dorsal-ventral polarity resulting in embryos with a rounded shape, which lacked ventral arms and had a strongly reduced dorsal region (Figure 8A). Molecular analysis revealed that ventral markers such as *chordin*, *foxA* or *brachyury* were expressed in *tbx2/3* morphants, albeit with reduced levels compared to controls (Figure 8A). A similar slight reduction was observed for the ciliary band markers *onecut/hnf6*, *fgfA* and *pax2/5/8*. In contrast, inhibition of *tbx2/3* function abolished the expression of several dorsal genes encoding transcription factors including *msx*, *dlx*, *irxA* and *atbf1* while the expression of other genes such as *smad6*, *glypican5*, *oasis* and *wnt5* appeared unaffected. These results identify *tbx2/3* as a key regulator of dorsal gene expression downstream of BMP2/4.

### IrxA: a negative regulator of *onecut/hnf6* downstream of *tbx2/3*

Since loss of BMP2/4 or Alk3/6 signaling causes ectopic expression of ciliary band genes in the dorsal ectoderm, it follows that in unperturbed embryos, a transcriptional repressor must act in the dorsal ectoderm downstream of BMP2/4 to prevent expression of ciliary band genes. Of the four transcription factors expressed in the dorsal ectoderm that we tested, only in the case of one of them did we observe robust ectopic expression of a ciliary band gene. This gene is *irxA*. In embryos injected with morpholinos against the *irxA* transcript, *onecut/hnf6* expression was strikingly expanded in the dorsal ectoderm (Figure 8B). This effect was very robust and the territory in which the ectopic expression of *onecut/hnf6* was observed was congruent with the expression territory of *irxA*. Interestingly, a small number of embryos injected with *irxA* morpholinos later developed with a thickened ectodermal region on the dorsal side that resembled an ectopic ciliary band (Figure 8B). This suggests that IrxA is a repressor of ciliary band genes downstream of BMP2/4.

### Onecut/hnf6: an upstream positive regulator of ciliary band gene expression

*onecut/hnf6* is of one of the earliest marker genes expressed in the presumptive ciliary band. *onecut/hnf6* morphants developed with a slightly reduced D/V axis but they clearly displayed a D/V polarity and a well-developed ciliary band (Figure 8C). Nevertheless, we found that the expression of several marker genes of the ciliary band was affected in the *onecut/hnf6* morphants. A reduced level of expression in the *onecut/hnf6* morphants was observed in the case of *pax2/5/8*, *foxG* and *dri* while in the case of *gfi1*, no expression was detected. *onecut/hnf6* is thus an upstream regulator of *gfi1*. Gfi proteins are conserved in *C. elegans* (Pag3), *Drosophila* (Senseless) and mice (Gfi1). In all three species, these zinc finger proteins play conserved roles in neural development [67]. Mice mutant for *gfi1* are deaf and ataxic while flies mutant for *senseless* lack sensory organs indicating that Gfi proteins regulate sensory organ development [67], [68]. One can therefore anticipate that Gfi1 likely plays a role in neural development in the sea urchin embryo as it does in vertebrates and in flies. Since *gfi1* is downstream of *onecut*, the ciliary band network therefore appears to be composed of at least two layers of zygotic factors.

### Positive and negative feed back loops downstream of Nodal and BMP signaling

#### Wnt8 signaling is required for maintenance of *nodal* expression

To identify the Wnt ligands responsible for maintenance of *nodal* expression, we examined the expression pattern of candidate genes encoding Wnt factors and tested them for their requirement to maintain *nodal* expression. Of the various Wnt ligands examined, only *wnt8* had a dynamic expression in the ectoderm at blastula stages (Figure 9A). Consistent with previous studies, we found that *wnt8* morphants are radialized [53]. In these embryos, *nodal* expression is strongly reduced or absent (Figure 9A). These findings indicate that, in addition to the essential *nodal* auto regulatory loop that is required early to maintain the expression of *nodal*, Wnt8 function is required zygotically to maintain *nodal* expression at blastula stages through a second positive feedback loop.

#### Chordin and Smad6 act as negative regulators of BMP signaling on the ventral and dorsal side respectively

We showed previously that most of the ectoderm of *chordin* morphants differentiated into an enlarged ciliary band, a finding that was difficult to reconcile with the demonstrated activity of Chordin as an inhibitor of BMP signaling. We found that the abnormal patterning of the ectoderm in the *chordin* morphants is associated with a strongly reduced expression of ventral genes such as *nodal*, *chordin*, *bmp2/4* and *goosecoid* and of dorsal genes such as *msx and wnt5* (Figure 9B). We confirmed that following injection of the *chordin* morpholino, *tbx2/3* was transiently ectopically expressed within the ventral ectoderm, consistent with the presence of ectopic BMP signaling in *chordin* morphants at mesenchyme blastula stage [26]. However, following this transient ectopic expression, *tbx2/3* expression was rapidly downregulated like that of other dorsal marker genes such as *msx* and *wnt5* (Figure 9B). Strikingly, expression of *onecut/hnf6* was de-repressed in the ectoderm of the *chordin* morphants at gastrula stages consistent with the reduced expression of *nodal* and *bmp2/4* in these embryos and with the development of an expanded ciliary band at later stages.

Overexpression of *smad6* phenocopied the BMP2/4 loss of function phenotype, resulting in partially radialized embryos with ectopic spicules and expanded ciliary band on the dorsal side (Figure 9C). This phenotype was correlated with the loss of *msx* expression and ectopic expression of *onecut/hnf6*. These observations together with the finding that expression of *smad6* is regulated by BMP signaling, strongly suggests that in the sea urchin as in vertebrates, Smad6 acts in a negative feedback loop required for the fine tuning of BMP signaling. High-level overexpression of *smad6* abolished the expression of Nodal target genes such as *chordin* and *foxA* and caused a massive ectopic expression of *onecut/hnf6* throughout the ectoderm indicating that one function of Smad6 may also be to restrict Nodal signaling to the ventral side.

## Discussion

### Essential roles of Nodal and BMP2/4 in patterning of the ectoderm along the D/V axis

In this study, taking advantage of the detailed phenotypic analyses and robust in situ hybridization procedures available in *Paracentrotus lividus*, we analyzed with a high level of spatial resolution the expression, the regulation and the function of most of the zygotic transcription factors and signaling molecules displaying restricted expression within the ectoderm of the sea urchin embryo. This analysis allowed us to assemble a gene regulatory network, the D/V GRN, which describes the regulatory interactions between these genes and provides a framework for understanding the developmental program responsible for patterning the embryo along the dorsal-ventral axis. Several interesting conclusions emerged from the resultant GRN. First, it provides a clear demonstration that the activities of Nodal and BMP2/4 account fully for the spatially restricted expression of all the known genes of this network: Nodal controls the expression of all the genes expressed specifically in the ventral ectoderm, and through BMP2/4, the expression of all the genes expressed specifically in the dorsal ectoderm. Both overexpression of these ligands and corresponding loss of function experiments produced very strong, all or none, effects consistent with the idea that Nodal and BMP2/4 are critical inputs that drive the D/V GRN. It should be noted that despite their essential roles, Nodal and BMP2/4 are certainly not the only ligands involved in D/V patterning of the ectoderm and other ligands more broadly expressed likely cooperate with Nodal and BMP2/4 to specify the ventral and dorsal regions. In particular, Nodal may bind to its receptor as a heterodimer with Univin, a GDF1/Vg1 ortholog, as shown in other models [24], [25] while BMP2/4 may heterodimerize with BMP5/8 to specify the dorsal ectoderm as shown in vertebrates and in *Drosophila* [69], [70]. Nevertheless, the key roles played by Nodal in this GRN together with the essential function of Nodal factors in D/V axis formation in vertebrates and basal chordates [71] reinforce the hypothesis that an ancestral function of Nodal may have been in the regulation of D/V axis formation in deuterostomes.

### Goosecoid, a repressor that drives a stomodeal regulatory sub circuit and represses ciliary band and dorsal genes

**Figure 10:**
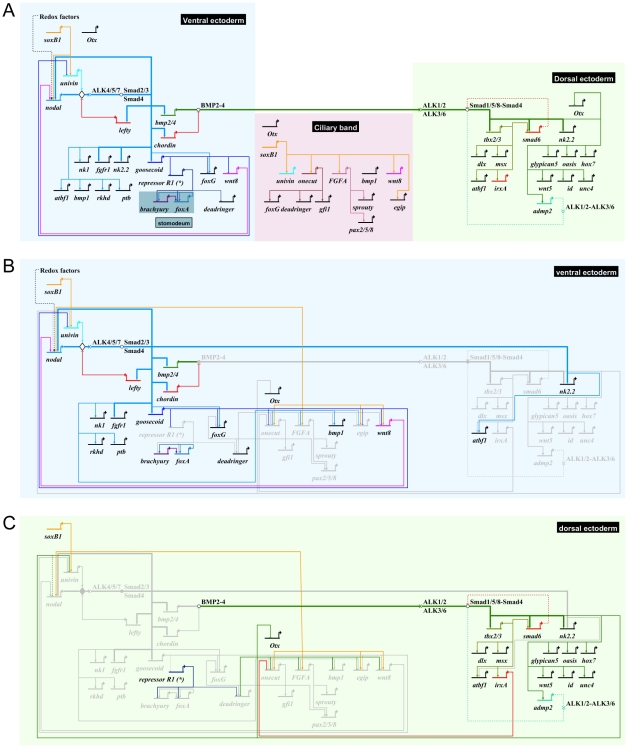
Representation of the gene regulatory network regulating regionalization of the ectoderm of the sea urchin embryo. (A) Biotapestry [123], [124] diagram of the provisional gene regulatory network describing the regulatory interactions that have been identified in this study. Arrows indicate positive transcriptional activation. Flat arrows indicate repression. The colored boxes represent the spatial domains as indicated. The linkages between Nodal and its immediate direct target genes as well as the linkages between BMP2/4 and its direct target genes are shown as bold arrows. The gene regulatory linkages sustained by solid evidence are presented as solid lines. The gene regulatory linkages that are hypothetical or suspected to be indirect are represented as dotted lines. Except for the bold arrows, no assumption on whether these interactions are direct or indirect is made. The linkage between Nodal and Delta, expressed in single cells of the ciliary band, is not represented here. (B) Gene regulatory interactions within the ventral ectoderm highlighting the repressive action of Goosecoid on ciliary band genes. The genes that are inactive are represented in light grey. (C) Gene regulatory interactions within the dorsal ectoderm highlighting the repressive action of irxA on *onecut/hnf6* and more generally of Nodal signaling on the expression of ciliary band genes.

A second key conclusion emerging from our D/V GRN is that in the sea urchin, Goosecoid is a key upstream element of a small regulatory circuit that controls mouth formation. In vertebrates ectopic expression of *goosecoid* promotes cell migration and induces incomplete secondary axes while loss of function studies implicate *goosecoid* in the function of the Spemann organizer and head formation [72]. The function of *goosecoid* during development of other deuterostome embryos has not been studied. In the sea urchin, previous studies reported that both overexpression and loss of function of *goosecoid* strongly perturbed establishment of the dorsal-ventral axis, however the target genes of *goosecoid* were not known and the role of this repressor within the ventral ectoderm remained largely unclear [35], [38], [40]. Our finding that *goosecoid* is a direct target of Nodal signaling strongly suggested that this gene could play a key role in specification of the ventral ectoderm downstream of Nodal. We have shown that Goosecoid likely regulates the expression of *deadringer* and *foxG* in the ventral ectoderm. Furthermore, we demonstrated that Goosecoid plays a critical role in mouth formation by regulating downstream target genes such as the stomodeal genes *brachyury* and *foxA*. This raises the possibility that an ancestral function of *goosecoid* may have been in the regulation of stomodeum formation. Consistent with this idea, *goosecoid* is expressed in the stomodeal region in both protostomes and deuterostomes and is co-expressed with *brachyury* and *foxA* in the oral region of cnidarians [73]. Since Goosecoid is a transcriptional repressor [74], this suggests that zygotic *goosecoid* activates *foxA* and *brachyury* by repressing the expression of a transcriptional repressor, the identity of which is presently unknown (Figure 10). Similar double repression mechanisms have been described in different GRNs. For example, in the sea urchin the skeletogenic mesoderm GRN, the repressor pMar has been proposed to repress *hes-C* as well as unidentified repressors to allow expression of genes specific of the PMC lineage [75], [76]. Similarly Schnurri, represses the expression of *brinker* to allow the expression of Dpp target genes in *Drosophila* imaginal discs [77]. One candidate for a repressor acting downstream of *goosecoid* is the transcriptional repressor ZEB1/Smad Interacting Protein 1 (Sip1) [78]. In the sea urchin embryo, Sip1 is expressed early in the presumptive ectoderm and its expression is downregulated at blastula stage, coincident with the onset of *goosecoid* expression [31] (see Figure S2 and S5). Experiments are currently being carried out in different labs to test this hypothesis.

Another important function of Goosecoid appears to be in the repression of ciliary band and dorsal genes. Overexpression of *goosecoid* potently repressed expression of ciliary band markers. Furthermore, knockdown of Goosecoid function caused ectopic expression of *univin*, *wnt8* and *foxG* in the ventral ectoderm. However, additional repressors likely cooperate with Goosecoid in this repression since inhibition of *goosecoid* function, unlike inhibition of *irxA* on the dorsal side, was not sufficient to derepress ciliary band markers genes such as *onecut* within the ventral ectoderm.

### Tbx2/3 an early mediator of BMP2/4 signaling

Tbx2/3 has a special status amongst dorsal genes since it is one of the earliest zygotic genes expressed on the presumptive dorsal side [40], [41]. Previous studies had shown that *tbx2/3* is expressed dynamically in a broad dorsal territory in all three germ layers and that its expression is regulated by BMP signaling [13], [26], [40], [41]. Indeed we showed that *tbx2/3* is a direct target of BMP2/4 signaling in the ectoderm and that its function is required for expression of several dorsally expressed transcription factors such as *msx*, *dlx*, *irxA* and *atbf1*. Intriguingly, previous studies in *Paracentrotus* failed to detect any D/V polarity defect in *tbx2/3* morphants [40]. In contrast, we found that *tbx2/3* is essential for D/V axis formation in this species. The reasons for this discrepancy are unclear. Interestingly, in vertebrates, *tbx2* is also a target of BMP4 signaling during D/V patterning of the optic cup [79]. Similarly, in hemichordates, which are positioned phylogenetically as the sister phylum of echinoderms, *tbx2/3* is a target of BMP2/4 suggesting that key genes that drive the D/V GRN are conserved in these two closely related phyla [80]. In vertebrates, *tbx2* and *tbx3*, unlike *brachyury*, which is a transcriptional activator, act as transcriptional repressors due to the presence of a strong repressor domain in their C-terminal region [81], [82]. It is therefore possible that the sea urchin Tbx2/3 protein also functions as a transcriptional repressor and that, like Goosecoid, it stimulates gene expression by relieving the repressive action of a transcriptional repressor. The identity of this hypothetical transcriptional repressor is presently unknown.

### IrxA- and BMP2/4-dependent repression of ciliary band gene expression

One of the most important findings of this study is the identification of *irxA* as a gene which acts downstream of BMP signaling to repress the ciliary band gene *onecut*. We previously reported that inhibition of BMP2/4 or Alk3/6 function causes an expansion of the presumptive ciliary band territory towards the dorsal side, and that this expansion is accompanied by the ectopic expression of the neural gene *onecut/hnf6* [26]. On the basis of this result we anticipated that one function of the BMP pathway in the dorsal ectoderm was to repress ciliary band gene expression and we postulated the existence of a BMP2/4 dependent repressor of ciliary band genes. We have now identified IrxA as one such repressor based on the following evidence. First, we showed that *irxA* expression is regulated by BMP2/4 signaling. Second, we showed that blocking *irxA* translation with morpholinos caused a robust ectopic expression of *onecut* in a sector of the dorsal ectoderm that coincides with the expression domain of *irxA*. Finally, it is established that Irx proteins can function as repressors by recruiting the Groucho Co-repressor [83], [84]. Since *irxA* is downstream of *tbx2/3* in the GRN, we might predict that blocking *tbx2/3* function should also result in ectopic expression of ciliary band genes. Surprisingly, we never observed ectopic expression of ciliary band marker genes in *tbx2/3* morphants. This observation is consistent with previous GRN studies, which reported that direct target genes are more strongly affected than indirect target genes or in other words, that when a perturbation affects the driver gene, it causes stronger effects on target genes than when the perturbation affects genes further upstream in the pathway [29]. However, the simplest explanation is that our *tbx2/3* morpholino may not be completely effective and that residual *irxA* expression may prevent ectopic expression of *onecut* in these embryos.

In vertebrates and in *Drosophila*, *irx* genes are involved in neural development [85]. In *Xenopus* for example, *irx1* promotes neural development by repressing *bmp4* expression in the neural plate. It was therefore surprising to find that in the sea urchin embryo, *irxA* acts downstream of BMP2/4 to negatively regulate neural marker genes. Nevertheless, the identification of *irxA* as a BMP2/4 dependent repressor of ciliary band gene expression strongly supports our proposal that the default state of the ectoderm in the absence of TGF beta signaling is the ciliary band and that the ectoderm is patterned by two successive inductive events that repress the ciliary band fate on the ventral and dorsal sides.

### Extracellular and intracellular modulators of Nodal and BMP2/4 signaling

#### *admp2* and *glypican5*: two components of a positive feedback loop downstream of BMP2/4?

Positive and negative feedback loops are essential components of gene regulatory networks. Indeed, in the sea urchin like in vertebrates, maintenance of *nodal* expression critically relies on autoregulation [12], [13], [14], [86]. Another auto regulatory loop that is highly conserved in vertebrates is found in the BMP signaling pathway with BMP signaling stimulating BMP transcription. However, in the sea urchin unlike in vertebrates, BMP2/4 signaling does not stimulate its own expression since transcription of *bmp2/4* occurs on the ventral side while BMP signaling occurs on the dorsal side [26]. While BMP2/4 signaling did not induce *bmp2/4* expression in dorsal cells, we found that BMP2/4 signaling instead induces expression of *admp2* and promotes expression of *glypican5*, a positive regulator of BMP signaling [26]. The dorsal expression of *admp2* and *glypican5* was expanded following overexpression of *bmp2/4* (Figure 3) and abolished in Nodal and/or BMP2/4 morphants (Figure 5, Figure 6) indicating that expression of these genes is regulated by BMP2/4 signaling. The positive regulation of *admp2* by BMP2/4 in the sea urchin contrasts with the regulation of *admp genes* in vertebrates. In *Xenopus* or zebrafish, *bmp4* and *admp* are expressed at opposite poles of the embryo and are under opposite transcriptional control: high levels of BMP signaling stimulate *bmp4* expression but repress transcription of *admp*, a property that has been correlated with the ability of dorsal halves of early embryos to regulate and re-establish a D/V axis [87], [88], [89], [90]. In the sea urchin, both dorsal and ventral halves of partial embryos can regulate and regenerate a complete D/V axis [91]. However, in this embryo, *bmp2/4* and *admp2* are not under opposite transcriptional control since BMP signaling does not repress but stimulates *admp*2 expression.

Whether *admp2* cooperates with BMP2/4 and contributes to specification of dorsal cells is presently not known but one can speculate on the possible role of this ligand as part of positive feed back loop downstream of BMP2/4 signaling. Therefore, while a BMP2/4 synexpression group does not exist in the sea urchin, BMP2/4 may activate a positive regulatory loop by inducing expression of *admp2* to reinforce BMP signaling within the dorsal territory. Future studies are required to clarify the function of ADMP2 during D/V patterning of the ectoderm and to determine if this BMP ligand contributes to the activation of pSmad1/5/8 signaling during normal and regulative development.

#### Wnt signaling and maintenance of *nodal* expression

Previous studies had shown that when the canonical Wnt pathway is inhibited by using a dominant negative form of TCF, expression of *nodal* is lost at blastula stage [13]. By analyzing *nodal* expression at early stages in *cadherin* injected embryos Angerer and colleagues showed that *nodal* expression is initiated normally in animalized embryos but that it is not maintained [92]. They further provided evidence that the loss of *nodal* expression in these animalized embryos is caused by the persistence of the expression of the FoxQ2 repressor in the ectoderm. Here we have identified Wnt8 as a ligand required to maintain *nodal* expression. The exact period when Wnt8 signals are required to maintain *nodal* expression is not known. During cleavage and blastula stage, *wnt8* is expressed in the vegetal pole region and starting at mesenchyme blastula stage, *wnt8* expression in a large belt of ectodermal cells (Figure 9A). One possibility is that Wnt8 signals emitted by the ventral and/or lateral ectoderm are responsible for the maintenance of *nodal* at the beginning of gastrulation. Alternatively, Wnt8 signals may be required earlier for the restriction of *foxQ2* expression at early blastula stages. Future studies are required to resolve this issue. It is remarkable that, during mouse embryogenesis, a Wnt signal is also required to maintain high levels of *nodal* expression in posterior cells through a TCF dependent enhancer and that in the absence of Wnt3, *nodal* transcription is initiated but it is not maintained [93]. Therefore, both in the sea urchin embryo and in the mouse embryo, in addition to the *nodal* auto regulatory loop, a second positive regulatory input from a Wnt ligand is required to maintain *nodal* expression.

#### Restriction of BMP signaling by Chordin is essential for D/V axis formation

Lastly, this study helped to resolve a paradox regarding the *chordin* loss of function phenotype. Chordin morphants display an expanded ciliary band covering the animal hemisphere, a reduced dorsal side and a pair of parallel spicules [26]. In vertebrates, *chordin* is expressed within the organizer and promotes neural differentiation by interrupting a BMP positive auto regulatory loop in the prospective neural ectoderm. In the sea urchin, *chordin* likely functions in a similar way by preventing BMP signaling within the ventral and dorsal ectoderm [26]. Since the function of Chordin as an inhibitor of BMP signaling is conserved, the reason why most of the ectoderm adopted a ciliary band fate instead of an epidermal fate in the *chordin* morphants remained unclear. We showed that in addition to ectopic BMP signaling, *chordin* morphants suffer from a strong downregulation of *nodal* expression leading ultimately to derepression of ciliary band and neural genes. One possibility is that the transient ectopic BMP signaling observed on the ventral side of *chordin* morphants interferes with Nodal signaling and interrupts the Nodal auto regulatory loop causing the subsequent loss of *bmp2/4* expression. Taken together, these findings highlight the crucial role played by *chordin* in D/V patterning of the ectoderm in the sea urchin embryo by showing that *chordin* is required for normal patterning along the whole dorsal-ventral axis.

### A new model of specification and regionalization of the ectoderm: the ciliary band as the default state of the ectoderm in the absence of Nodal and BMP signaling

The results obtained in this study largely support this idea that the default state of the ectoderm in the absence of Nodal and BMP signaling is a ciliary band-like ectoderm that expresses a number of neural genes and that Nodal and BMP2/4 restrict this ciliary band fate by specifying the ventral and dorsal ectoderm. The first hint that the default state of the ectoderm in the absence of TGF beta signaling is the ciliary band is that several genes whose expression is later restricted to the ciliary band territory are expressed throughout the ectoderm at earlier stages. This is for example apparent for *fgfA*, *univin* and *wnt8*, which are expressed in a belt of cells that includes most of the presumptive ectoderm at blastula stages. The expression of *fgfA*, *univin* and *wnt8* is subsequently repressed on the ventral and dorsal sides during gastrulation thereby restricting the expression of these genes to the ciliary band domain. Several additional lines of evidence support the idea that the default state of the ectoderm in the absence of TGF beta signaling is a ciliary band and neural fate and that alternative ectodermal fates must be induced by active signaling. First, overexpression of both *nodal* and *bmp2/4* strongly antagonized the expression of ciliary band and neural markers such as *onecut*, *foxG* and *gfi1*, with *bmp2/4* leading to a very potent inhibition of ciliary band formation. Second, in the *lefty* morphants the ciliary band failed to form while in the absence of Nodal and BMP2/4 signaling, the ventral and dorsal ectodermal regions were not specified and most of the ectoderm differentiated instead into a thickened ciliated ectoderm that resembled the ciliary band ectoderm and expressed all tested ciliary band markers. These ciliary band markers were de-repressed throughout the ventral and dorsal ectoderm in the *nodal* morphants while in the absence of BMP2/4, which acts as a dorsal inducer, or of *alk3/6*, which is required to transduce BMP2/4 signals, only specification of the dorsal ectoderm was perturbed and ectopic expression of these ciliary band genes was detected only on the dorsal side. A third argument is that the presumptive ciliary band territory is also a region in which *fgfA* and *vegf* are expressed and where MAP kinase activity is high [48], [56], [94]. Studies in vertebrates have shown that the activity of the MAP kinase ERK inhibits both BMP signaling and neuralization by phosphorylating Smad1 in the linker region thereby preventing its nuclearization. We thus predict that during normal development of the sea urchin embryo, the high MAP kinase activity present in the lateral ectoderm promotes neural fates within the presumptive ciliary band by inhibiting the activity of pSMAD1/5/8 and pSMAD2/3. Thus, in the absence of Nodal and BMP signaling, signals such as FGFA that are normally present at the level of the lateral ectoderm are ectopically expressed in the ventral and dorsal regions where they may promote ectopic neuron formation [26], [27]. One last but crucial argument that supports our model of the ciliary band as a default state of the ectoderm in the absence of TGF beta signaling is that we identified *irxA* and possibly Goosecoid as repressors of a subset of ciliary band genes downstream of Nodal or BMP signaling. One read-out of Nodal and BMP2/4 signaling therefore appears to be active repression of the ciliary band fate as we had predicted [26].

Yaguchi and colleagues previously demonstrated that in the absence of Wnt signaling, most of the ectoderm differentiates as a neurogenic ectoderm that expresses markers of the animal pole [27]. Since many ciliary band genes are also expressed in the animal pole, it could be argued that the ectopic expression of ciliary band marker genes observed following inhibition of Nodal or BMP signaling also reflects an expansion of the animal pole domain. This can be ruled out for several reasons. First, we showed that the expression of animal pole markers such as *foxQ2*, is unaffected in Nodal morphants or in embryos treated with a pharmacological inhibitor of the Nodal receptor. Second, Yaguchi et al. showed that the number and location of serotonergic neurons of the apical organ are unaffected by inhibition of Nodal signaling. Importantly, we showed that *pax2/5/8*, which is expressed in the vegetal part of the ciliary band but not in the animal pole region behaved exactly like the other ciliary band marker genes and was strongly derepressed in the ventral and dorsal ectoderm of Nodal morphants. Taken together these observations indicate that the lateral ectoderm of the prospective ciliary band, not the animal pole domain, is expanded in the Nodal morphants.

**Figure 11:**
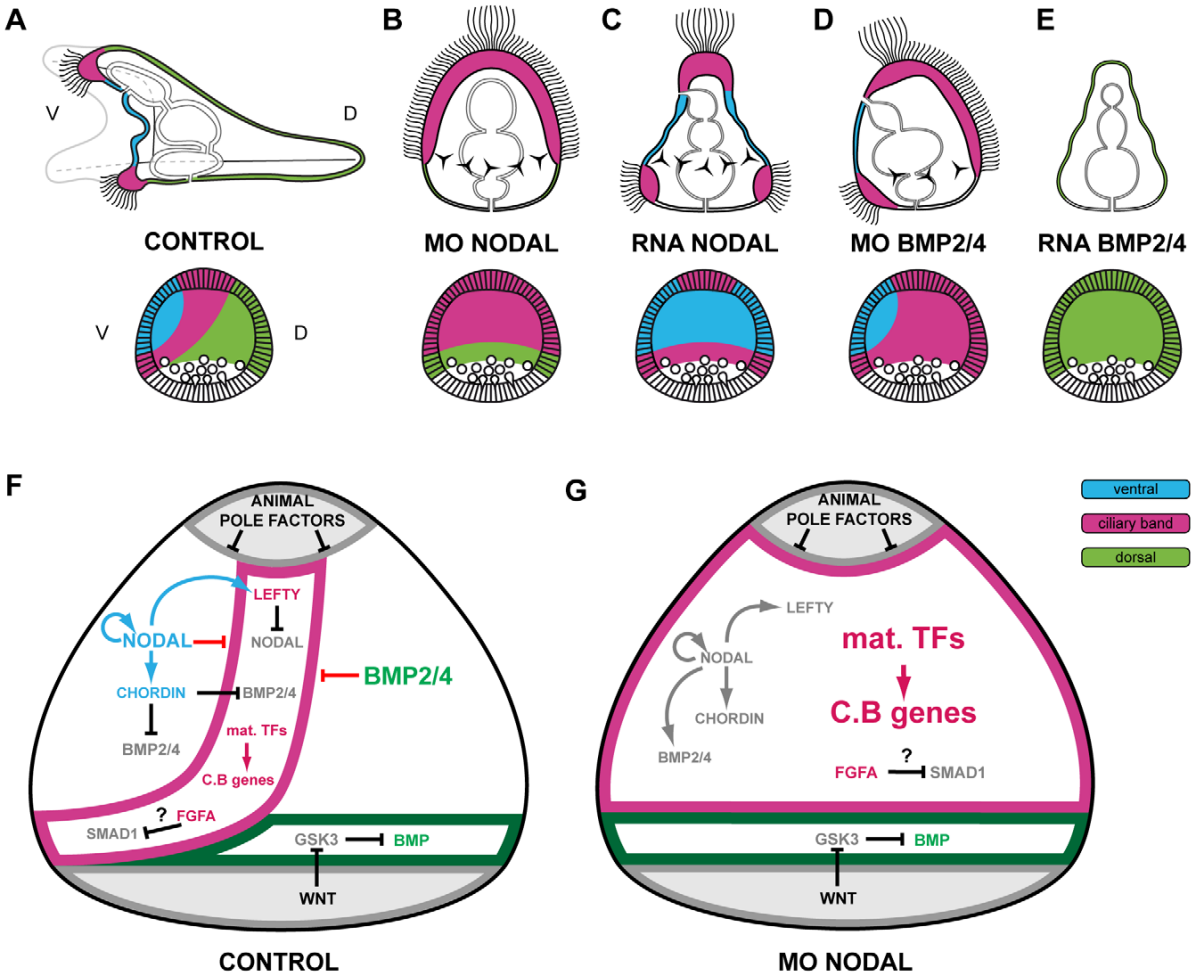
Changes in identity of ectodermal territories following perturbations of Nodal or BMP signaling and novel model of ectoderm patterning. Schemes describing the morphology of control embryos and perturbed embryos. (A) control embryo. The thick ciliated epithelium of the ciliary band is restricted to a belt of cells at the interface between the ventral and dorsal ectoderm. (B) Nodal morphant. Most of the ectoderm differentiates into an expanded large ciliary band. An animal pole domain is nevertheless present in these embryos as shown by the presence of the apical tuft and at the molecular level by the expression of apical domain marker genes. In these embryos, the ectoderm surrounding the blastopore differentiates into dorsal ectoderm. (C) embryo overexpressing Nodal. Most of the ectoderm differentiates into ventral ectoderm. A ciliary band-like ectoderm forms at the animal pole and in the ectoderm surrounding the blastopore. (D) BMP2/4 morphants. An ectopic ciliary band forms in the dorsal ectoderm in addition to the normal ciliary band. (E) *bmp2/4* overexpressing embryo. All the ectoderm has a dorsal identity. The animal pole domain is largely absent. The triradiated stars represent the spicule rudiments. (F) Proposed model for regionalization of the ectoderm of the sea urchin embryo through restriction of the ciliary band fate by Nodal and BMP signaling. Maternal factors such as SoxB1 promote the early expression of ciliary band genes within the ectoderm. Nodal signaling on the ventral side promotes differentiation of the ventral ectoderm and stomodeum and represses the ciliary band fate probably through the activity of Goosecoid as well as of additional repressors. Nodal induces its antagonist Lefty, which diffuses away from the ventral ectoderm up to the presumptive ciliary band territory. Within the ventral ectoderm, Nodal induces expression of *bmp2/4* and of its antagonist *chordin*. Chordin prevents BMP signaling within the ventral ectoderm and probably within the presumptive ciliary band region. At blastula stages, protein complexes containing BMP2/4 and Chordin can diffuse towards the dorsal side to specify dorsal fates. In the dorsal ectoderm, BMP signaling strongly repress the ciliary band fate partly by inducing the expression of the *irxA* repressor. A high level of MAP kinase activity resulting from FGFA signaling in the lateral ectoderm likely contributes to maintain a low level of Nodal and BMP signaling within the presumptive ciliary band region by phosphorylating Smad1/5/8 and Smad2/3 in the linker region, which inhibits their activity. The presence of Chordin and Lefty in the prospective ciliary band allows expression of ciliary band genes to be maintained in this region. The ectoderm surrounding the blastopore differentiates into dorsal ectoderm likely because it receives Wnt signals that antagonize GSK3 and promote BMP signaling. (G) In the absence of Nodal signaling, both the ventral and the dorsal inducing signals are not produced, ciliary band genes are not repressed and unrestricted MAP kinase signaling promotes differentiation of the ventral and dorsal ectoderm into neural ectoderm and ciliary band. The genes or proteins that are inactive are represented in light grey.

Our study suggests that specification of the ciliary band is likely initiated by a combination of maternal factors such as SoxB1 and by zygotic factors such as FGFA, Otx and Onecut/Hnf6 whose expression is initiated independently of the Nodal and BMP2/4 signals (Figure 10). These zygotic genes initially show a broad expression in the ectoderm, which then becomes restricted to the presumptive ciliary band by the activity of transcriptional repressors such as Goosecoid and IrxA expressed in the ventral or dorsal ectoderm downstream of Nodal or BMP2/4. Collectively our results suggest that the neural ectoderm of the ciliary band forms in a territory that is devoid of Nodal and BMP2/4 signaling (Figure 11). On the dorsal side, inhibition of BMP signaling appears to be sufficient to trigger formation of the ciliary band as was observed in BMP2/4 or Alk3/6 morphants or in embryos injected with low doses of *smad6* mRNA. Similarly, on the ventral side, inhibition of Nodal signaling is sufficient to initiate formation of a ciliary band since BMP signaling does not occur on the ventral side but on the dorsal side [26]. In this case, ectopic neural differentiation likely results from inhibition of ventral differentiation. This highlights that, in the sea urchin ectoderm, preventing ventral cells to differentiate downstream of Nodal signaling promotes neural differentiation just as efficiently as inhibiting BMP signaling on the dorsal side. Similarly, in zebrafish embryos, inhibition of Nodal signaling causes the transfating of prospective mesendodermal cells into neural cells [95], [96] and in the mouse, lack of Nodal signaling causes precocious neural differentiation [97]. Therefore, in the sea urchin embryo like in vertebrate embryo models, neural differentiation can result both from inhibition of BMP signals as well as from inhibition of other signals that regulate the fate of early blastomeres and allocate cells to embryonic territories and germ layers.

In summary, our results show that in the sea urchin embryo, the neurogenic territory of the ciliary band is not induced by an interaction between the ventral and dorsal territories as previously suggested [98], but that it represents the default state of the ectoderm in the absence of Nodal and BMP signaling. Nodal and BMP2/4 may therefore be regarded as factors that are required to prevent premature differentiation of ectodermal cells into neural cells as much as factors that are required for specification of the ventral and dorsal ectoderm.

### Comparison with previous GRNs

Another recent GRN analysis of ectoderm specification in *S. purpuratus* was performed using nanostring technology [29]. A comparison of the architecture of the gene regulatory networks derived from this study and ours reveals the expected similarities but also some major differences. A common central element in the architecture of both networks is the critical dependence of dorsal genes on non-autonomous signaling by BMP2/4, a feature already proposed previously [13]. Another point of convergence is that both studies pointed to *goosecoid* and *tbx2/3* as important early zygotic genes downstream of Nodal and BMP2/4: both studies identified *brachyury* as a downstream target of Goosecoid, and *dlx* and *irx*A as downstream targets of Tbx2/3. Finally, both studies identified *foxG* and *deadringer* as downstream targets of Nodal.

The first important difference in the architecture of the two proposed networks is that whereas our study defines the default state of the ectoderm in the absence of Nodal and BMP signals as a ciliary band-like ectoderm, the network proposed by Su et al. largely ignores formation of the ciliary band. Another important difference between the two studies concerns the dependence of ventral genes on Nodal. Su et al. argued that only part of the oral ectoderm specification system is downstream of Nodal [29]. According to the authors, a number of regionally expressed genes including *onecut/hnf6*, *otx2*, *lim1*, and *foxA*, are activated “specifically in the oral ectoderm…exactly the same with or without *nodal*”, leading them to speculate that hypothetical Nodal independent early oral ectoderm signals regulate these genes in the ventral ectoderm. We do not agree with this interpretation, since from our in situ analysis, it is clear that these genes cannot be considered as oral-specific markers. Furthermore, we showed that the expression of *onecut/hnf6*, *otx2*, *lim1*, and *foxA* in the presumptive ectoderm region of Nodal morphants was not regionalized, consistent with the absence of any oral territory in these embryos. The expression of *onecut/hnf6* and *otx2* is first initiated in a territory much larger than the ventral ectoderm, before subsequently becoming restricted to either to the ciliary band (*onecut/hnf6*) or to a broader territory that also includes the ventral ectoderm (*otx2*). We thus interpret the continued expression of *onecut/hnf6* and *otx2* in the ectoderm as reflecting adoption of a ciliary band character by the entire ectoderm. Concerning *foxA*, the *nodal*-independent detection of the mRNA reported by Su et al is undoubtedly due to the abundant expression of this gene in a distinct endodermal territory, which, unlike the oral ectoderm expression, is largely Nodal-independent. The *foxA* example highlights the importance of using methods that allow spatial resolution to analyze the expression of genes with complex expression patterns in epistasis experiments.

According to Otim and colleagues and Su and colleagues, two genes, *onecut/hnf6* and *deadringer*, play essential roles in the DV GRN. Using an “unconventional morpholino” that targeted a sequence 660 bp downstream of the first ATG but that did not target a splice junction, Otim et al. reported that “inhibition” of *hnf6/onecut* function eliminated D/V polarity and caused a radialized phenotype that strikingly resembled the Nodal loss of function. Using the same reagent, Su et al. expanded this analysis and further argued that a positive regulatory input from *onecut/hnf6* is required for the expression of several key regulators such as *nodal*, *goosecoid*, *lefty*, *chordin*, and *bmp2/4* [29], [46]. These results are highly surprising since morpholinos are predicted to be ineffective at blocking translation when they target sequences after the first 25 bases following the initiator ATG [99], [100]. Using two different and more conventional morpholinos targeting the 5′ leader or the translation start site of the *P. lividus hnf6/onecut* transcript, we were unable to reproduce either the striking *hnf6/onecut* morphant phenotypes originally reported by Otim and colleagues or the effects on *nodal*, *goosecoid*, *lefty*, *chordin*, and *bmp2/4* reported by Su and colleagues. It is therefore very unlikely that *onecut/hnf6*, which is expressed only transiently within the ventral ectoderm, plays the crucial role proposed by these authors in this gene regulatory network. Regarding *deadringer*, Su et al. found that *deadringer* morphants display a much reduced expression of ventral genes such as *goosecoid*, *NK1* and *hes* as well as a strongly reduced expression of dorsal genes such as *irx*, *nk2.2* and *tbx2.3*. Again, these results are surprising since the published cDNA sequence of *deadringer* used by Su et al. to design their morpholino as well as the associated predictions of the translation start site of the protein are probably incorrect and correspond to a truncated protein sequence as suggested by our sequence analysis of the genomic *S. purpuratus deadringer* locus and the analysis of the *deadringer* cDNAs in *Paracentrotus* (Figure S1). In addition, using two different morpholinos against the *P. lividus deadringer* transcript, we were unable to reproduce the published drastic effects of *deadringer* morpholinos on the expression of ventral and dorsal marker genes. It is therefore also unlikely that *deadringer* plays the role that it had been previously attributed in the *S. purpuratus* GRN.

Finally, it has been argued that specific aboral differentiation genes such as *CyIIIa* and *spec1* are transcriptionally activated in the aboral ectoderm long before late blastula and that this implied the existence of an early asymmetry in the aboral ectoderm that affected transcriptional activity. Su et al. postulated that this asymmetry may be a redox gradient that would directly regulate the transcriptional activity of aboral genes such as *CyIIIa* and *tbx2/3*. Our results oppose this view. In *Paracentrotus*, the ectodermal expression of *tbx2/3* is essentially lost following inhibition of Nodal or BMP2/4 signaling. While it is true that a residual *tbx2/3* expression is observed in the Nodal morphants at gastrula stage, this expression is restricted to the vegetal most regions and therefore likely reflects the response of this gene to signals that act along the animal-vegetal axis rather than response to a redox gradient along the D/V axis. Furthermore, in *Paracentrotus*, expression of *CyIII* genes is first ubiquitous and only becomes restricted to the dorsal ectoderm at mesenchyme blastula stage (see Figure S5), coinciding with the nuclear translocation of pSmad1/5/8 in dorsal cells. In other words, we never observed any marker gene that was expressed specifically in the dorsal ectoderm before the onset of BMP signaling i.e. at late blastula stage. Our observations therefore do not support the view that the asymmetrical *CyIIIa* or *tbx2/3* expression is driven by an early red-ox gradient, at least not in *Paracentrotus*, but suggest that their expression is more likely driven by differential Nodal and BMP signaling along the dorsal-ventral axis.

### Nodal and BMP inhibition as an ancestral mechanism of neural induction?

A comparison of the mechanisms of neural induction in different species reveals both similarities and divergences regarding the signaling pathways involved. In *Xenopus*, inhibition of both Nodal and BMP signaling appears to be essential for neural induction, although FGF signaling is likely implicated in the early steps of this process [101], [102]. Similarly, in mammals, both Nodal and BMP signaling have been involved in neural differentiation, the strongest evidence being that most epibast cells of mouse embryos mutant for *nodal* or *bmpr1* display widespread and precocious expression of anterior neural markers [97], [103]. In the chick and in zebrafish, there is strong evidence that FGF signaling regulates neural induction partly through the regulation of expression of BMP ligands and of BMP antagonists [104], [105], [106]. In contrast in ascidians, which are basally branching but divergent chordates, FGF signals are the key players in neural induction by directly regulating the expression of neural markers such as *otx* [107]–[109]. Inhibition of BMP signaling does not appear to play a role in this process [110] while Nodal plays a distinct, inductive role in patterning of the neural plate [111]. Similarly, in hemichordates, which together with the echinoderms form a sister group of the chordates and have a diffuse neural system, BMP signaling does not appear to play a role in the choice between neural and epidermis [80].

Our experiments in the sea urchin embryo show that inhibition of Nodal and BMP signaling is central to neural induction in echinoderms and that in the absence of Nodal or BMP signaling, most cells of the ectoderm differentiate into a neurogenic ectoderm. Since BMP signaling also regulates neural differentiation in insects [112] and annelids [113], it appears likely that inhibition of Nodal and BMP signaling may have been an ancestral mechanism to specify neural cells not only in deuterostomes but also perhaps in bilateria, and thus that the neural specification mechanisms used in ascidians and hemichordates have diverged during evolution.

Although in the sea urchin inhibition of Nodal causes the ventral ectoderm to adopt ultimately a neurogenic ectodermal fate, it should be kept in mind that our experiments also suggest that Nodal may have an early and positive role in specification and/or patterning of the neurogenic territory of the ciliary band since we showed that Nodal promotes the expression of Delta in a subpopulation of ciliary band cells and drives the early expression of the neural gene *foxG*. Therefore, in the sea urchin as in chordates, in addition to its general inhibitory role on neural induction, Nodal may also play a positive role in specification and/or patterning of the neural territory [111], [114], [115].

In conclusion, this large scale, systematic GRN analysis has allowed us to identify a number of key gene regulatory interactions and to build a provisional gene regulatory network describing specification of the three main ectodermal territories of the sea urchin embryo. It has not only uncovered key and probably ancient regulatory sub circuits that drive morphogenesis of the ectoderm, but has also allowed us to propose a new model of how specific regions of the ectoderm are induced over a default state, and of how the ectoderm is patterned by successive rounds of induction by TGF beta ligands. This relatively simple model captures most of the results derived from the functional analyses of Nodal and BMP2/4 in the sea urchin embryo and provides testable predictions for futures studies. Finally, our study illustrates the power of the GRN based approaches which can provide a global perspective on a set of genes regulating a biological process, explaining how this process works and what happens when it fails.

## Materials and Methods

### Animals, embryos, and treatments with recombinant proteins and inhibitors

Adults sea urchins (*Paracentrotus lividus*) were collected in the bay of Villefranche-sur-Mer. Embryos were cultured as described previously [116], [117]. When required, fertilization envelopes were removed by adding 2mM 3-amino-1,2,4 triazole 1 min before insemination to prevent hardening of this envelope followed by filtration through a 75µm nylon net. SB431542 (10 µM in sea water) was diluted from stocks solutions in DMSO, and embryos incubated in 24 well plates protected from light. In controls experiments, DMSO was added at 0.1% final concentration. NiCl_2_ was used at 0.5 mM. SB431542 and nickel treatments were performed continuously starting 30 min after fertilization. Continuous treatments with recombinant mouse Nodal (1µg/ml) and BMP4 proteins (0.5 µg/ml) (R&D) started at the 16-cell-stage and used embryos lacking the fertilization envelope. We verified with a set of 10 genes that RNA overexpression and recombinant proteins produced equivalent effects for both Nodal and BMP.

To determine if marker genes are direct or indirect targets of Nodal or BMP4 signaling, embryos at the swimming blastula/late blastula, early mesenchyme blastula stage or at gastrula stage from which the fertilization envelope had been removed were treated for 2h with recombinant proteins in the presence or absence of protein synthesis inhibitors. To block protein synthesis, puromycin or emetine was added at a final concentration of 360µM (200µg/ml) or 5µM (10µg/ml) respectively using stock solutions prepared in DMSO. In control experiments embryos were treated with 0.1% DMSO or with Puromycin at 200µg/ml or emetine at 10µg/ml. Development of the treated embryos was usually arrested 30 min after addition of the inhibitor, an indication of the effectiveness of the reagent and after 3–4h, all the treated embryos underwent a massive and brutal apoptosis, an effect characteristic of treatments with protein synthesis inhibitors. In the case of *nodal*, *bmp2/4*, *lefty*, *goosecoid*, *fgfr1*, *chordin*, *nk2.2*, *tbx2.3*, treatments were performed at the swimming blastula stage. In the case of *nk1*, *foxA*, *brachyury*, *foxG*, *dlx*, *hox7*, *id*, *irxA*, *glypican5*, *cyIIIa*, *admp2*, *smad6* and *msx*, treatments were performed at the early mesenchyme blastula sage. In the case of *deadringer*, *atbf1*, *msx*, *wnt5*, *irxA* and *dlx* treatments were also performed at gastrula stage. Short treatments with Nodal or BMP4 failed to induce ectopic expression of any marker gene at gastrula stage suggesting that most of the genes expressed at this stage are indirect targets of Nodal and BMP2/4 or alternatively that at this stage, ectodermal territories are resistant to respecification by exogenous Nodal or BMP4.

### cDNA sequences and cloning of full-length transcripts

Most of the genes analyzed in this study were discovered in the course of a random in situ hybridization screen using cDNA libraries from various stages (T. Lepage unpublished). Additional marker genes were discovered in a second in situ screen aimed at analyzing the expression profiles of all the transcription factors and signaling molecules expressed during early sea urchin development [30] using a *Paracentrotus lividus* EST library (http://goblet.molgen.mpg.de/cgi-bin/webapps/paracentrotus.cgi). When the isolated clones were incomplete, full-length cDNA sequences were obtained either by screening cDNA libraries with conventional methods and sequencing the corresponding clones. In certain cases, 5′RACE was performed using the Smart RACE kit (Clontech) to obtain the 5′ sequences. A list of all the *Paracentrotus* transcripts analyzed in this study with a summary of their temporal and spatial expression patterns is provided in Table 1 together with the corresponding accession numbers and original references describing these genes. Note that in the case of *deadringer*, the sequence of the *Paracentrotus lividus* clones diverged significantly from the published *Strongylocentrotus purpuratus* sequence. The published *S. purpuratus deadringer* transcript is predicted to encode a 490 amino acid protein. However, all the 13 independent *deadringer* cDNA clones that we sequenced encoded a protein 100 amino acids longer on the N-terminal side. Furthermore, translation of the *S. purpuratus* genomic sequence upstream of the predicted first ATG revealed the presence of a much longer open reading frame compared to the published *deadringer* protein sequence that encoded a protein highly similar to the deduced protein sequence from *Paracentrotus* (see Figure S1). This indicates that the previously published *deadringer* mRNA sequence was probably incorrect on the 5′ end and that the predicted *deadringer* protein sequence deduced from this mRNA was truncated. Since morpholinos fail to block translation when their target sequence is located after the first 25 bp following the initiator ATG [100], the conclusions derived from previous functional studies of *deadringer* in *S. purpuratus*, which relied on a truncated sequence, are probably erroneous.

### Characterization of the temporal and spatial expression of regulatory genes of the network

For each gene of the network, a detailed analysis of the expression pattern was performed using digoxygenin labeled probes and in some cases, the temporal expression was analyzed by Northern blotting to verify maternal expression and to determine the exact onset of zygotic gene expression (Figure S2). In situ hybridization was performed following a protocol adapted from Harland [118] with antisense RNA probes and staged embryos. For marker genes expressed in ventral or dorsal territories at early stages, and for genes with complex expression profiles, double in situ hybridization was performed to confirm the orientations of the expression pattern. In this case, the two probes were hybridized and developed simultaneously. Probes derived from pBluescript vectors were synthesized with T7 RNA polymerase after linearization of the plasmids by NotI, while probes derived from pSport were synthesized with SP6 polymerase after linearization with SfiI. Control and experimental embryos were developed for the same time in the same experiments. Two color in situ hybridization was used following the procedure of Thisse et al. [119].

### Overexpression analysis

For overexpression studies the coding sequence of the genes analyzed was amplified by PCR with the Pfx DNA polymerase (Invitrogen) using oligonucleotides containing restriction sites and cloned into pCS2 [120]. Capped mRNAs were synthesized from NotI-linearized templates using mMessage mMachine kit (Ambion). After synthesis, capped RNAs were purified on Sephadex G50 columns and quantitated by spectrophotometry. RNAs were mixed with Tetramethyl Rhodamine Dextran (10000 MW) or Texas Red Dextran (70000 MW) or Fluoresceinated Dextran (70000 MW) at 5 mg/ml and injected in the concentration range 100–800µg/ml. The *nodal*, *bmp2/4*, *fgfA*, *univin*, *alk3/6QD*, and *chordin* pCS2 constructs have been described in Duboc et al. (2004), Röttinger et al. (2008), Range et al. (2007) and Lapraz et al. (2009). The pCS2 *goosecoid* construct is described in [40]. RNA derived from the following additional constructs were made (the cloning sites are indicated in parenthesis): *pCS2foxA* (ClaI-XbaI); *pCS2deadringer* (EcoRI-XhoI); pCS2*foxG* (ClaI-XhoI); pCs2*smad6* (EcoRI-XbaI); *pCS2pax2/5/8* (BamHI-XhoI); *pCS2tbx2/3* (BamHI-XhoI); *pCS2msx* (BamHI-XhoI); *pCS2nk2.2* (BamHI-XhoI).

### Perturbation analysis with morpholinos

Morpholino antisense oligonucleotides were obtained from GeneTools LLC (Eugene, OR). The *nodal*, *BMP2/4*, *Alk4/5/7,Alk3/6*, *univin*, *lefty* and *soxB1* morpholinos are described in [12]–[14], [26]. Since morpholinos can have side effects or display toxicity or produce variable reductions in gene activity [121], we designed and tested several morpholinos for each gene. A pair of morpholinos that did not display toxic effects was selected for further use (a morpholino was considered toxic if it caused developmental arrest during cleavage or a massive cell death at the onset of gastrulation when injected at low doses (0.1–0.3 mM)). In the cases of *nodal*, *bmp2/4*, *alk3/6*, *Alk4/5/7*, *univin* and *soxB1*, the efficiency of the morpholino to downregulate the expression of previously characterized targets genes was systematically assessed in control experiments [13], [14], [26]. The phenotypes observed for *nodal, bmp2/4, brachyury chordin, foxA, fgfA, goosecoid, irxA, lefty, tbx2/3, dlx, msx, onecut/hnf6, soxB1, univin, wnt8* morpholinos were considered specific since they were confirmed with a separate, non-overlapping morpholino. In the case of *alk3/6*, *alk4/5/7* and *nodal*, a rescue experiment had previously been performed demonstrating the specificity of these reagents [13], [14], [26]. The phenotypes observed were always consistent with the zygotic expression pattern of the targeted genes and with previous well-established functional data [13], [14], [26], [35], [42], [122]. We did not observe inconsistent phenotypes among several knockdowns except in one case, in which knocking down Tbx2/3, an upstream regulatory gene of *irxA*, did not cause the same effect on the IrxA target gene *onecut/hnf6* as knocking down *irxA* itself suggesting that the *tbx2/3* morphant phenotype is a hypomorphic phenotype and not a null. In the case of the ventrally expressed genes *nodal* and *bmp2/4*, we observed strong non autonomous effects consistent with the demonstrated translocation of BMP2/4 from the ventral to the dorsal ectoderm and with the role of BMP2/4 as relay downstream of Nodal [26]. In contrast, we never observed strong effects on the expression of ventral markers by morpholinos targeting genes expressed dorsally. In three cases, (*dlx*, *msx*, *foxG*) a morphological phenotype was consistently observed but molecular analysis failed to detect significant perturbations in the expression of the genes analyzed. Other morpholinos pairs (*deadringer*, *hox7*, *nk2.2*, *oasis*, *wnt5*) gave very weak or not always reproducible phenotypes. Molecular analysis on embryos injected with these morpholinos failed to detect significant and reproducible changes in gene expression in any of the ventral, dorsal or ciliary band markers genes that we tested. In a few cases, (*atbf1*, *klf2/4*) all the morpholinos synthesized were highly toxic and were not studied further. The loss of function phenotypes of *29D*, *tubulinß3*, *egip*, *CyIIIa*, *admp2*, *fgfr1*, *pax2/5/8*, *unc4*, *nk1*, *id*, *rkhd* and *ptb* and *otx* were not analyzed in this study and these genes were only used as markers in the following experiments. The sequences of all the morpholino oligomers used in this study are listed below. The most efficient morpholino of each pair is labeled with a star.

alk4/5/7 Mo 1: TAAGTATAGCACGTTCCAATGCCAT

alk3/6: Mo1: TAGTGTTACATCTGTCGCCATATTC

brachyury Mo1: AGCATCGGCGCTCATAGCAGGCATA

brachyury Mo2*: CTGGCAGAAGATGTACTTCGACGAT

bmp2/4 Mo1*: GACCCCAGTTTGAGGTGGTAACCAT

bmp2/4 Mo2: CATGATGGGTGGGATAACACAATGT

chordin Mo1*: GGTATAAATCACGACACGGTACATG

chordin Mo2: CGAAGATAAAAACTTCCAAGGTGTC

deadringer Mo1: TGCTCGCGGTAACAAGTGATTCCAT

deadringer Mo2: TTATATGGCAAAGGACTTCTACAGC

dlx Mo1: CCCACGTCAAATGAATACATCAACA

dlx Mo2: AAACACGTTTAGAATCCTCACGACT

fgfA Mo1: ACTTTCATCCATTTTCGCTTTCATG

fgfA Mo2*: ACACATTTTGGATACTTACAGCTCC

foxA Mo1: CATGGGTTCCTCCTTGAAATCCACG

foxA Mo2*: TGAAAGATTAAAGTAGCACAGTCAG

foxG Mo1*: TCCGATGAATGTGCATGAAAAACTG

foxG Mo2: CTTCTTGCTAAATACCAAGTTGGAG

goosecoid Mo1*: TGTCTGGAAGGTAATAGTCCATCTC

goosecoid Mo2: AGATCAGAGCTAACCACTTAGGACG

hnf6/onecut: Mo1: AGCCGCTGGACCTCAAACGCGAAGA

hnf6/onecut Mo2*: AAAATGATAATGTGGTCTCCGTCGC

hox7 Mo1: TGACGAAATACGAACTCGAACTCAT

hox7 Mo2: ACCACTTCATTAATAGCCAAAACCT

irxA Mo1: ATTGTGGATAACTGCTCGTCGTCAT

irxA Mo2: TTGTTGAAATCAACTTTGAGACGAT

Lefty Mo1: GGAGCGCCATGAGATAATTCCATAT

Lefty Mo2: GGAGATGGGCAAAATATGAAGATAC

msx Mo1: CGACTTGATGGAAGAAAATTATTCC

msx Mo2 : TTATCGCTTTAAGAATGACCAAGGA

NK1 Mo1: AAGCATTGAGAATCCCTAAAACTGC

NK1 Mo2: CATGTGCTCTGTTCAGACGGTCAAC

nk2.2 Mo1: ATCAACATTCATACGATGTCTCTAT

nk2.2 Mo2: ATAGTTAATTCCACACCACCCACTT

nodal Mo1*: ACTTTGCGACTTTAGCTAATGATGC

nodal Mo2: ATGAGAAGAGTTGCTCCGATGGTTG

tbx2/3 Mo1: TCGACGAACCACCAAATCTTGAGCA

tbx2/3 Mo2* : TCGGCAAAAGCCTCCGAGTCCAAAT

Oasis Mo1: CTCTTCACCTAAAAGCCCATCCATG

Oasis Mo2: CCAATTTGGGCCGTAGTCGAGGGAC

soxB1 Mo 1*: GACAGTCTCTTTGAAATTAGACGAC

soxB1 Mo2: GAAATAAAGCCAAAGTCTTTTGATG

univin Mo1*: ACGTCCATATTTAGCTCGTGTTTGT

univin Mo2: GTTAAACTCACCTTTCTAAACTCAC

wnt8 Mo1: GAACAACTGCCGTAAAGATATCCAT

wnt8 Mo2*: AACAGTCCAAATATGAAGTTCAAAC

As a control for defects related to injection and egg quality, we used morpholinos directed against the hatching enzyme gene: 5′-GCAATATCAAGCCAGAATTCGCCAT-3′ or against the Nemo like kinase transcript -5′-TCGGAGGCAGACCAGCAGCGAGAAA-3′. Embryos injected with either of these morpholinos at 1mM normally develop into pluteus larvae. Morpholinos oligonucleotides were dissolved in sterile water and injected at the one-cell stage together with Tetramethyl Rhodamine Dextran (10000 MW) at 5 mg/ml. For each morpholino a dose-response curve was obtained and a concentration at which the oligomer did not elicit non-specific defect was chosen. Approximately 2–4 pl of oligonucleotide solution at 0.5 mM were used in most of the experiments described here. For morphological observations, about 150–200 eggs were injected in each experiment. To analyze gene expression in the morphants a minimum of 50–75 injected embryos were hybridized with a given probe. All the experiments were repeated at least twice and only representative phenotypes observed in more than 80% of embryos are presented.

## Supporting Information

Figure S1 Sequence analysis of the deadringer cDNA. (A) Partial genomic sequence of the *deadringer* locus and predicted protein sequence encoded by the first two exons of the *Strongylocentrotus purpuratus deadringer* gene. The sequence represents the first two exons and the first intron of the deadringer gene. The translation start site predicted from the analysis of several *Paracentrotus lividus* cDNAs and the predicted protein sequence are shown. The solid bar in position 1285 of the nucleotide sequence indicates the ATG previously thought to encode the initiator methionine. (B) Sequence comparison of the predicted *Paracentrotus lividus* Deadringer protein sequence with the predicted *Strongylocentrotus purpuratus* Deadringer protein sequence deduced from the genomic sequence. Note that the published Spu-deadringer protein sequence starts at position 1285 and therefore is truncated of the first 100 aminoacids.
(0.92 MB PDF)

Figure S2 Northern blot analysis of *nodal*, *bmp2/4*, *goosecoid* and *sip1* expression during development of the sea urchin embryo. Embryonic stages are : egg (E), 16 cells (16), 32 cell-stage (32), 60 cells (60), very early blastula (B1), early blastula (B3), swimming blastula (SB), mesenchyme blastula (MB), early gastrula (EG), late gastrula (LG), prism (Pr), Pl pluteus. Loading control is 28S mRNA.
(3.37 MB TIF)

Figure S3 Expression of ectodermal marker genes following ventralization with nickel chloride - Embryos were treated with nickel chloride starting after fertilization and the expression of ventral, dorsal and ciliary band genes was analyzed at the relevant stages. Treatment with nickel caused an expansion of all the ventral marker genes, and strongly repressed the expression of dorsal and ciliary band marker genes. The effects of Nickel treatments on marker gene expression are largely similar to those resulting from Nodal overexpression. However, a few intriguing differences can be noticed. For example, nickel treatment more efficiently suppressed the expression of markers of the animal pole region and ciliary band markers, such as *gfi1* or *onecut/hnf6*, than *nodal* overexpression. However, in the case of *fgfA*, the opposite result was observed with *nodal* overexpression more efficiently repressing expression of *fgfA* in the animal pole and vegetal ectoderm regions than nickel treatment. lv, lateral view, vv, vegetal pole view, fv, frontal view.
(8.54 MB TIF)

Figure S4 Expression of ectodermal marker genes following inhibition of the Nodal receptor with the pharmacological agent SB432542. Embryos were treated with SB431542 at 10 µM starting after fertilization and expression of ventral dorsal or ciliary band marker genes was analyzed at the relevant stages. Blocking Nodal signaling at the level of the receptor abolished the expression of ventral and dorsal marker genes and caused ectopic expression of ciliary band genes. lv, lateral view, vv, vegetal pole view, fv, frontal view.
(6.02 MB TIF)

Figure S5 Expression pattern of *CyIII* and *sip1* during early development of *Paracentrotus lividus*. (A) The probe used corresponds to the CyIIIb transcript and crosshybridizes with CyIIIa. CyIII genes are expressed ubiquitously at early and hatching blastula stages. Starting at late blastula stage, *CyIII* transcripts accumulate preferentially in the ectoderm on the dorsal side. At mesenchyme blastula and gastrula stages, *CyIII* expression is restricted to the dorsal ectoderm and ventral SMCs. (B) At early blastula stage *sip1* is expressed in two thirds of the embryo. Double in situ hybridization a probe for *skeT*, a gene expressed in the skeletogenic mesodermal precursors indicates that *sip1* is expressed mostly in the presumptive ectoderm derived from the animal hemisphere. lv, lateral view, vv, vegetal pole view.
(2.57 MB TIF)

Table S1 Gene knockdown and overexpression experiments used to construct the network.
(0.07 MB PDF)

Table S2 Gene knockdown without detectable effect as examined by in situ hybridization.
(0.06 MB PDF)
