# Supplementary material for: Ancestral Regulatory Circuits Governing Ectoderm Patterning Downstream of Nodal and BMP2/4 Revealed by Gene Regulatory Network Analysis in an Echinoderm
Source: PLoS Genet. 2010 Dec 23;6(12):e1001259. doi: 10.1371/journal.pgen.1001259 (PMC3009687; doi:10.1371/journal.pgen.1001259)
Supplement: Figure S1 — Sequence analysis of the deadringer cDNA. (A) Partial genomic sequence of the deadringer locus and predicted protein sequence encoded by the first two exons of the Strongylocentrotus purpuratus deadringer gene. The sequence represents the first two exons and the first intron of the deadringer gene. The translation start site predicted from the analysis of several Paracentrotus lividus cDNAs and the predicted protein sequence are shown. The solid bar in position 1285 of the nucleotide sequence indicates the ATG previously thought to encode the initiator methionine. (B) Sequence comparison of the predicted Paracentrotus lividus Deadringer protein sequence with the predicted Strongylocentrotus purpuratus Deadringer protein sequence deduced from the genomic sequence. Note that the published Spu-deadringer protein sequence starts at position 1285 and therefore is truncated of the first 100 aminoacids. (0.92 MB PDF) [file pgen.1001259.s001.pdf]

A

```
   1 CAATTATTTCATGACCGGTGTATTAAACCTGCGTGAGTCAAGTGGACAAAGTCATTCAAACAGTTAGACAGAAGCTCGCAGTCATCACACAATCCTTCTCTGTTAGTCCAATCATGACAC 120
     ---------|---------|---------|---------|---------|---------|---------|---------|---------|---------|---------|---------|
 121 TCGCTCATAAGGTGTAAACTTTTTCGACGAGAGAGTTATTTTGTCAAGTCAAAACAATAAATTAGGAATCGCGGGATAACCGACTTTGCAACCAGTGATTTGAGCGCTCGAGCGGAGATC 240
     ---------|---------|---------|---------|---------|---------|---------|---------|---------|---------|---------|---------|
 241 GGGTGAACCACCGGGCGGTCAGCTACGGGCGTGAGGAGAGCGAAAGTTCGGAGACTCCACCTGTTAATATCAGGCGATCTCATCGAGAAAATTTCTTCAACATAAACGGGAAatggaatc 360
   1                                                                                                                   M  E  S  3
     ---------|---------|---------|---------|---------|---------|---------|---------|---------|---------|---------|---------|
 361 actttataccgcggcaacaaaacatatgGTGAGTTCTTGACATTCTTGGTTCTGTTATGGGATGGGAATTTCCCCTGTTAATATAAATAATTAAAATTTATCGGTATTGCGGTCAATAAT 480
   4  L  Y  T  A  A  T  K  H  M ---------|---------|---------|---------|---------|---------|---------|---------|---------|   12
 481 TGCCGATTTCTGGTGCCTGGGTGCATGAAGAGACCGATGTTTTTTTCTTTTTCATGTGTGTATGTGTATGTTTTTTTGACTGATTGTTTTTGTGTCTGTTAATTGTATTTACGAAATCAT 600
     ---------|---------|---------|---------|---------|---------|---------|---------|---------|---------|---------|---------|
 601 GTATGATGTATACATTGATGTTTCGCATTTATATTTCAGGTCCAAATTTGTTTTCTTTTGCTTTAAATATGTTTCTTTTTTCTATGTATTCATCTTTTACTTCGCTAGTTTTTGTTTTGT 720
     ---------|---------|---------|---------|---------|---------|---------|---------|---------|---------|---------|---------|
 721 TATTCAGGAAAGTGTCTGATTGTTAAGACCATTTTGCAGTTTTTACAATAACAACAAGTATATTGCAAATTAGACAGTAAAAGCCCCTTCAATATAAAGCTAAATGATTCAGATGAAAGC 840
     ---------|---------|---------|---------|---------|---------|---------|---------|---------|---------|---------|---------|
 841 CTATAAAAATATTGATGATATAACAAGGGCCCAAGTAGTGACCTATTTGCAAAAATATTTGTTCACGAGAATTTATGATGTTAGCAATTGCTTTTCTTCCCTCCCTTCTTTCTCTCTATA 960
     ---------|---------|---------|---------|---------|---------|---------|---------|---------|---------|---------|---------|
 961 TGCTATCAAATCAGGCTTTTATGACAATTCTCGTAAATGTTATCTTTTTTTAATGCAGtcaccgaggcatcttgctatgtttgagatgtcacgaagagagatcctcgagaaatatatcca 1080
  13                                                           S  P  R  H  L  A  M  F  E  M  S  R  R  E  I  L  E  K  Y  I  H 33
     ---------|---------|---------|---------|---------|---------|---------|---------|---------|---------|---------|---------|
1081 tcaaaatggggaagatgagagtgaataccaagagaaagtgaggagaatgagtgtggatactaccagtgatcacgaagaggaacgtgttaaggaggagcgggctggagctacagagattga 1200
  34  Q  N  G  E  D  E  S  E  Y  Q  E  K  V  R  R  M  S  V  D  T  T  S  D  H  E  E  E  R  V  K  E  E  R  A  G  A  T  E  I  D 73
     ---------|---------|---------|---------|---------|---------|---------|---------|---------|---------|---------|---------|
1201 tgagatggagagaaggagggtcatggaggagcagaggcggatcattgaggaacagaggaggatagtagaagagcagcgccggaggatggttgaagaccagcgaagacagctcatggagga 1320
  74  E  M  E  R  R  R  V  M  E  E  Q  R  R  I  I  E  E  Q  R  R  I  V  E  E  Q  R  R  R  M  V  E  D  Q  R  R  Q  L  M  E  E 113
     ---------|---------|---------|---------|---------|---------|---------|---------|---------|---------|---------|---------|
1321 agaggatgaggagaggaggctcattcttgaggagcagaggcggcggatgatgagagcagatcgagatgaagaagaggaggaggaggaggaggaagaggaggaggaaagggaagaagatga 1440
 114  E  D  E  E  R  R  L  I  L  E  E  Q  R  R  R  M  M  R  A  D  R  D  E  E  E  E  E  E  E  E  E  E  E  E  E  R  E  E  D  D 153
     ---------|---------|---------|---------|---------|---------|---------|---------|---------|---------|---------|---------|
1441 tggaaggcgatcagaggatgagatgagggaggatgaacccccctggtagacgtgaaaccagccacgcccatattgatctgaacatgatgcgagccaatgcccacctcaaggagatgatgga 1560
 154  G  R  R  S  E  D  E  M  R  E  D  E  P  P  G  R  R  E  T  S  H  A  H  I  D  L  N  M  M  R  A  N  A  H  L  K  E  M  M  D 193
     ---------|---------|---------|---------|---------|---------|---------|---------|---------|---------|---------|---------|
1561 taagaaccgtcgatttgtttctacccgcctggaggaacccatcacccagtctcctcctctcaccaacggtagtaaccatgacaacgaccatgatccctacctctcccaccgagcagctca 1680
 194  K  N  R  R  F  V  S  T  R  L  E  E  P  I  T  Q  S  P  P  L  T  N  G  S  N  H  D  N  D  H  D  P  Y  L  S  H  R  A  A  H 233
     ---------|---------|---------|---------|---------|---------|---------|---------|---------|---------|---------|---------|
1681 tggaggcagtcctgatttaccacactcctacatgaaagcagcgcatcctctcatcaagaaagaggatggaatcgctaagatggagatggatattgggttgaaagatgagatgaagatggg 1800
 234  G  G  S  P  D  L  P  H  S  Y  M  K  A  A  H  P  L  I  K  K  E  D  G  I  A  K  M  E  M  D  I  G  L  K  D  E  M  K  M  G 273
     ---------|---------|---------|---------|---------|---------|---------|---------|---------|---------|---------|---------|
1801 agcaggcctggaagacagagatggagacaaacctcagacggaatggtcttttgaagaacagttcaaacag 1870
 274  A  G  L  E  D  R  D  G  D  K  P  Q  T  E  W  S  F  E  E  Q  F  K  Q    296
```

B

```
S.P  MESLYTAATKHMSPRHLAMFEMSRREILEKYIHQNGEDESEYQEKVRRMSVDTTSDHEEE
P.L  MESLVTASTKHLSPRHLAMYEMSRREILEKYIHQNGQDEAEYHERVRRMSVDTASDHDEE

S.P  RVKEERAGATEIDEMERRRVMEEQRRIIEEQRRIVEEQRRRMVEDQRRQLMEEEDEERRL
P.L  RIKEER-GAMENEEMERRRVMEEQRRIIEEQRRIVEEQRRRMVEEQRRQLMEEEDEERRL

S.P  ILEEQRRRMMRADRDEEEEEEEEEEEEEREEDDGRRSEDEMREDEPPGRRETSHAHIDLN
P.L  LLEEQRRRMMRPDHDEEEEEEEEHRDDD----DGGHSGEEEMREDELSGRRDTSHAHIDLN

S.P  MMRANAHLKEMMDKNRRFVSTRLEEPITQSPPLTNGSNHDNDHDPYLSHRAAHGGSPDLP
P.L  MMRANSHFKEMIDKNRRFVSARPEDTISHSPPLTNGSVHDNDHDPYLSHRAGHGGSPDLP

S.P  HSYMKAAHPLIKKEDGIAKMEMDIGLKDEMKMGAGLEDRDGDKPQTEWSFEEQFKQLYEL
P.L  HSYMKAAHPLIKKED--AKMEMDVGLKDDLKIGGGLDDRDGEKPQTEWSFEEQFKQLYEL

S.P  STDSKRKEFLDDLFSYMQKRGTPVNRIPIMAKQVLDLYELYNLVVAKGGLVEVINKKQWR
P.L  SSESKRKEFLDDLFSFMQKRGTPVNRIPIMAKQVLDLYELYNLVVAKGGLVEVINKKQWR

S.P  EITKGLNLPASITSAAFTLRTQYMKYLYPYECEKKGLSSPSELQSAIDGNRREGRRPSYH
P.L  EITKGLNLPASITSAAFTLRTQYMKYLYPYECEKKSLSSPSELQSAIDGNRREGRRPSYH

S.P  SPHMHPRGPSLAYHHGLDLHTPSGSPPPTMIPHPSRIPTLPPRLSPSTSPIEDDHPVPLG
P.L  SPHMHPRGPTLAYHHGLDLHTPSGSPPPTMIPHPSRIPTLPTRLSPSTSPIEDDHPAPLT

S.P  FPRQGTLSHAAMLAELAERGSIPPPSKRSLLAEEHHHRLLQLQQQHLVMPTAHLKVSSAR
P.L  LPRQGALNHAAMLAELAERGSIPPPAKRSLLAEEHHQRLLQLQQQHLLMSTAHMKVSSAR

S.P  AHPENGMPLFEMRGDNSLVMSIELNSVLYQGVLYPRGGTRS-----SLDRDTSTPV
P.L  AHPENGMPLFEMRGDNSLVMSIELNNVLYQGVLYPRGVPGHPLIGIPVPRSSASPK
```

Fig. S1
