# Supplementary figures and images for: Ancestral Regulatory Circuits Governing Ectoderm Patterning Downstream of Nodal and BMP2/4 Revealed by Gene Regulatory Network Analysis in an Echinoderm
Source: PLoS Genet. 2010 Dec 23;6(12):e1001259. doi: 10.1371/journal.pgen.1001259 (PMC3009687; doi:10.1371/journal.pgen.1001259)

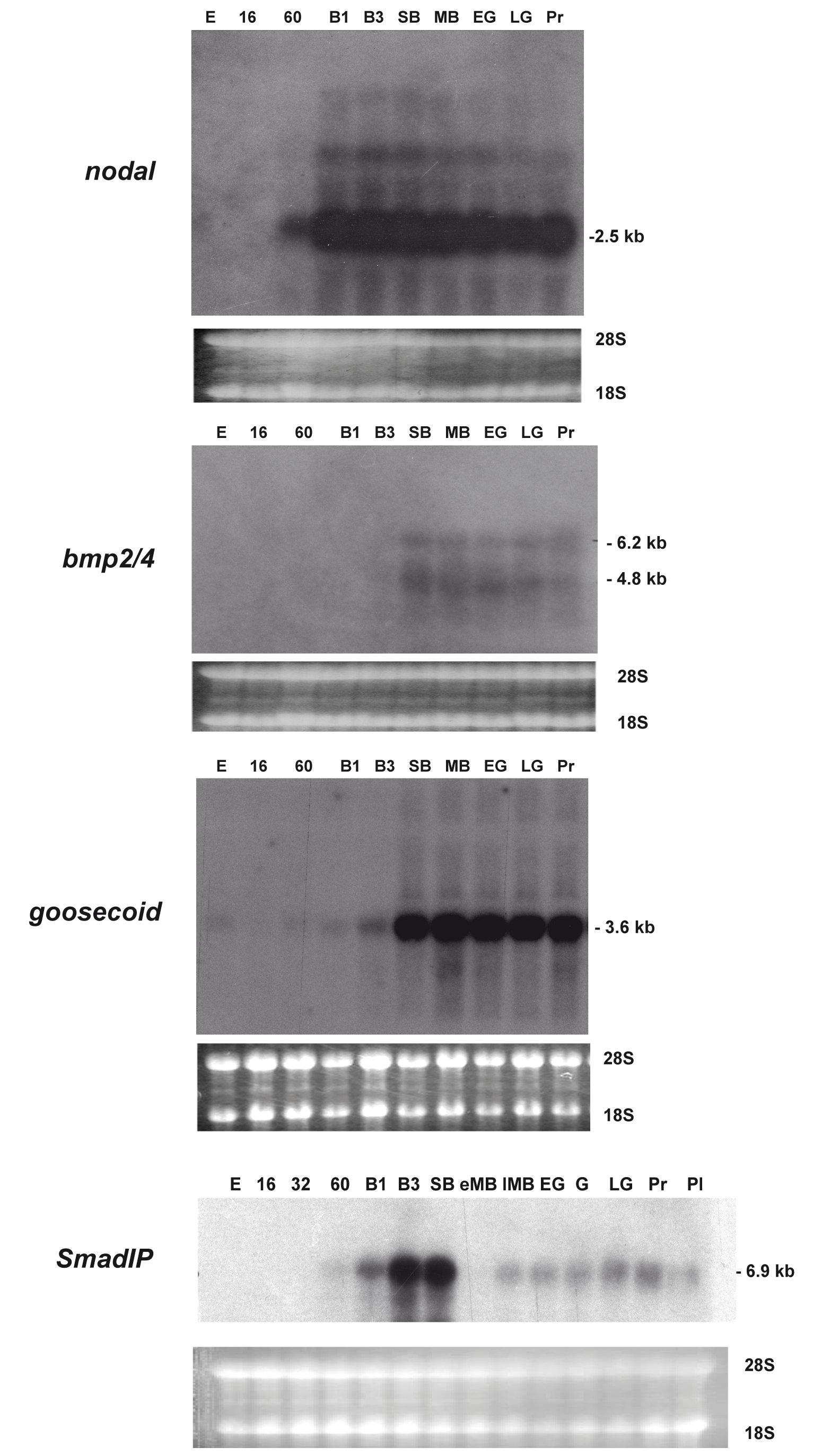

Supplement: Figure S2 — Northern blot analysis of nodal, bmp2/4, goosecoid and sip1 expression during development of the sea urchin embryo. Embryonic stages are : egg (E), 16 cells (16), 32 cell-stage (32), 60 cells (60), very early blastula (B1), early blastula (B3), swimming blastula (SB), mesenchyme blastula (MB), early gastrula (EG), late gastrula (LG), prism (Pr), Pl pluteus. Loading control is 28S mRNA. (3.37 MB TIF) [file pgen.1001259.s002.tif]

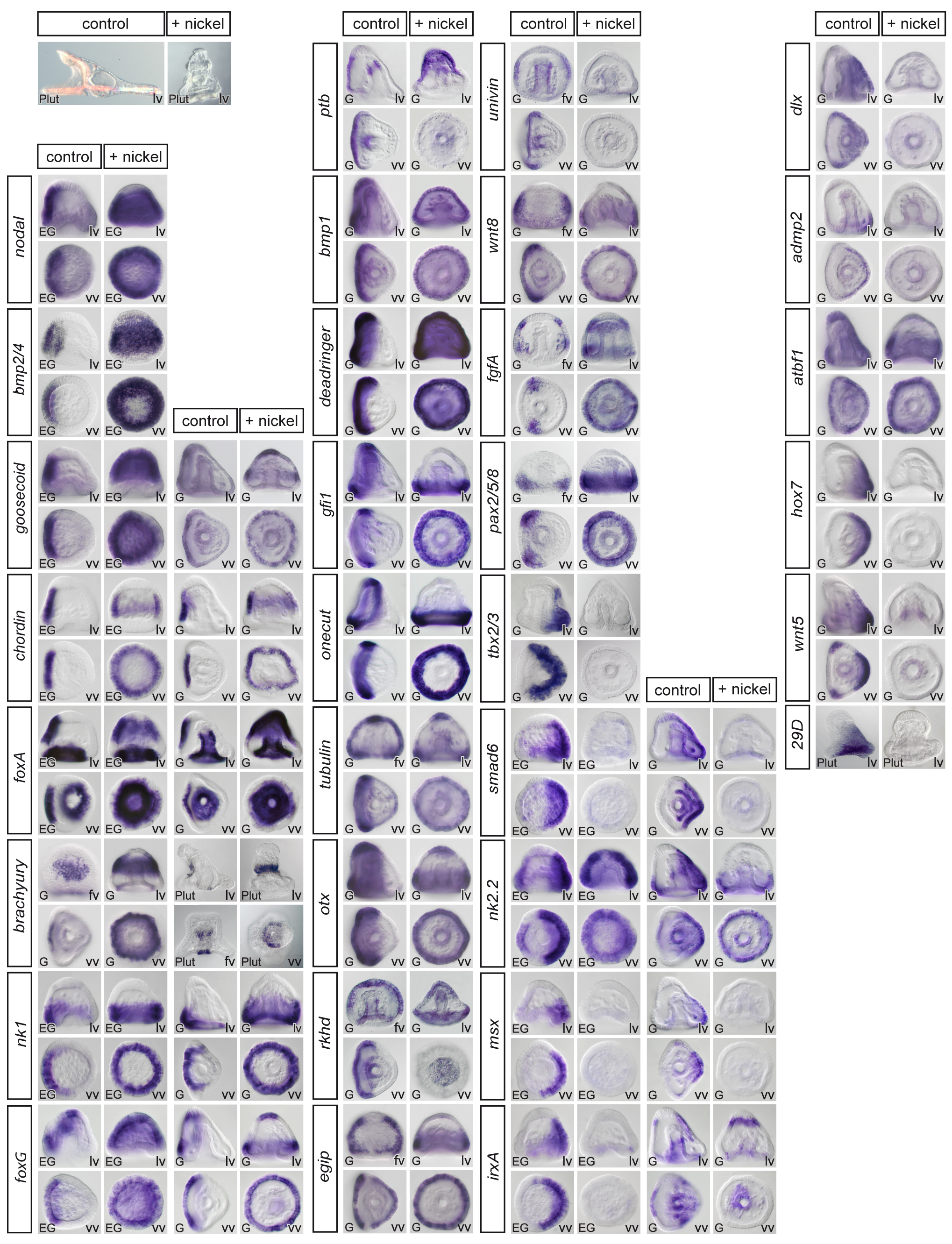

Supplement: Figure S3 — Expression of ectodermal marker genes following ventralization with nickel chloride - Embryos were treated with nickel chloride starting after fertilization and the expression of ventral, dorsal and ciliary band genes was analyzed at the relevant stages. Treatment with nickel caused an expansion of all the ventral marker genes, and strongly repressed the expression of dorsal and ciliary band marker genes. The effects of Nickel treatments on marker gene expression are largely similar to those resulting from Nodal overexpression. However, a few intriguing differences can be noticed. For example, nickel treatment more efficiently suppressed the expression of markers of the animal pole region and ciliary band markers, such as gfi1 or onecut/hnf6, than nodal overexpression. However, in the case of fgfA, the opposite result was observed with nodal overexpression more efficiently repressing expression of fgfA in the animal pole and vegetal ectoderm regions than nickel treatment. lv, lateral view, vv, vegetal pole view, fv, frontal view. (8.54 MB TIF) [file pgen.1001259.s003.tif]

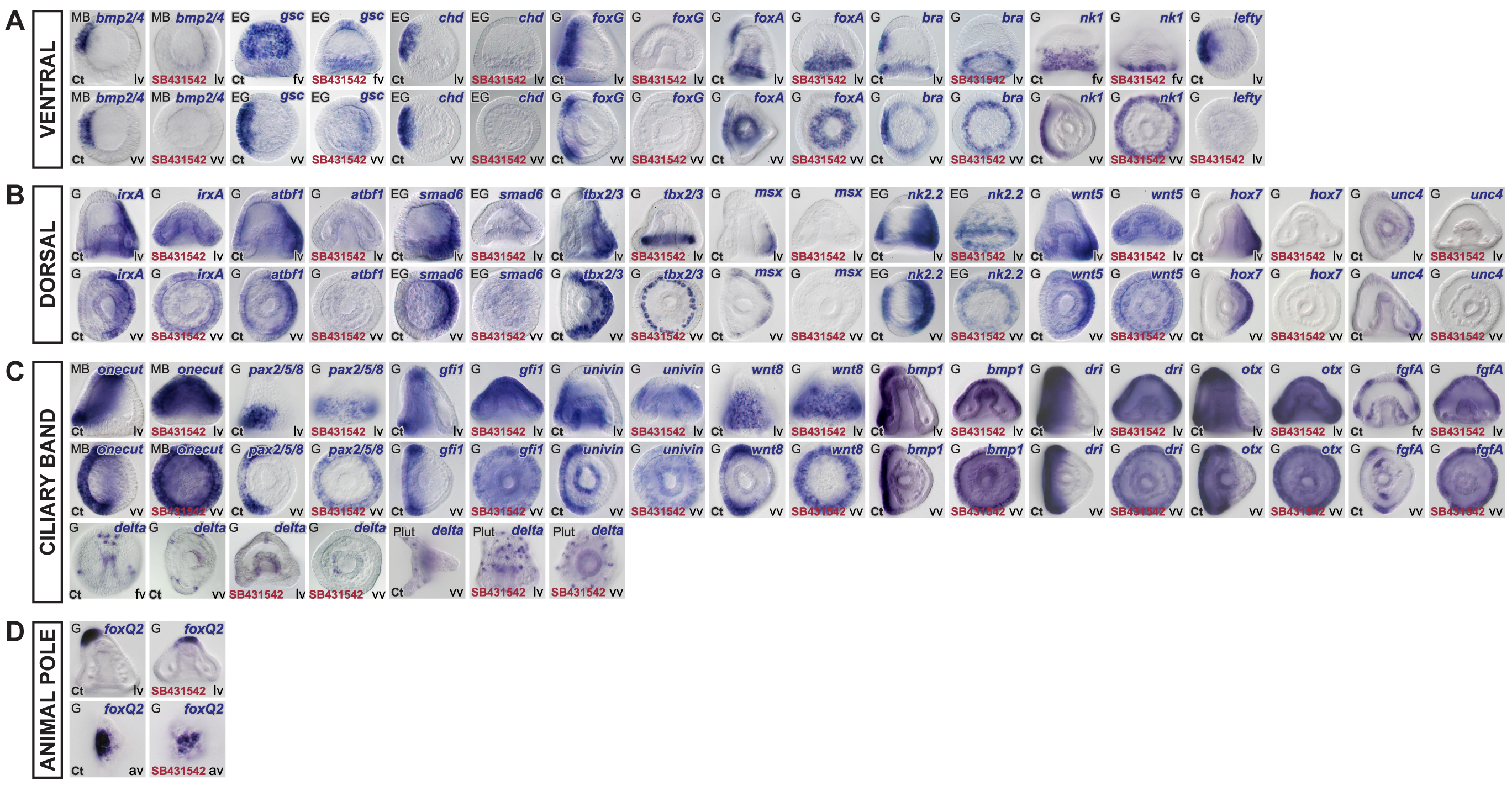

Supplement: Figure S4 — Expression of ectodermal marker genes following inhibition of the Nodal receptor with the pharmacological agent SB432542. Embryos were treated with SB431542 at 10 µM starting after fertilization and expression of ventral dorsal or ciliary band marker genes was analyzed at the relevant stages. Blocking Nodal signaling at the level of the receptor abolished the expression of ventral and dorsal marker genes and caused ectopic expression of ciliary band genes. lv, lateral view, vv, vegetal pole view, fv, frontal view. (6.02 MB TIF) [file pgen.1001259.s004.tif]

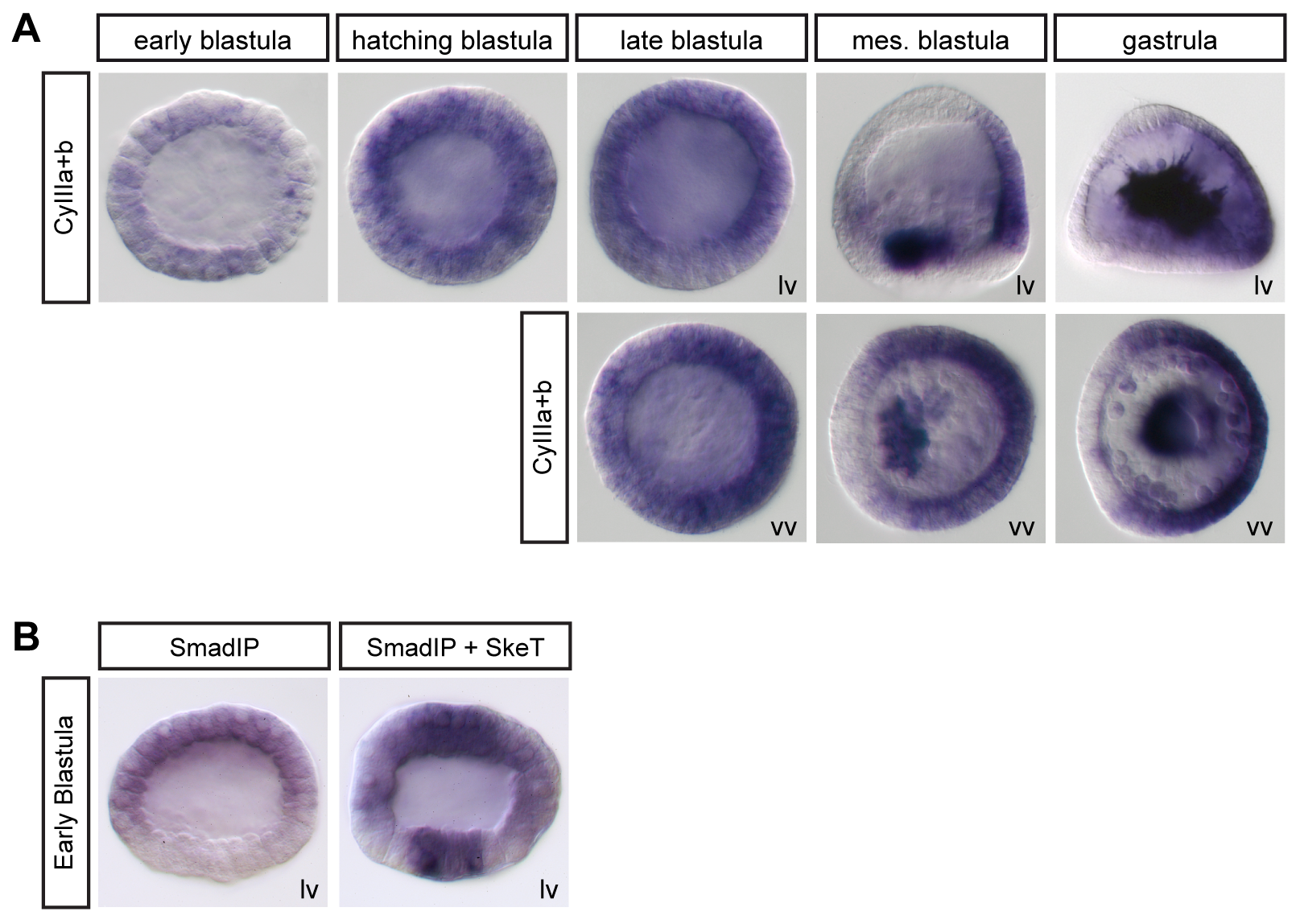

Supplement: Figure S5 — Expression pattern of CyIII and sip1 during early development of Paracentrotus lividus. (A) The probe used corresponds to the CyIIIb transcript and crosshybridizes with CyIIIa. CyIII genes are expressed ubiquitously at early and hatching blastula stages. Starting at late blastula stage, CyIII transcripts accumulate preferentially in the ectoderm on the dorsal side. At mesenchyme blastula and gastrula stages, CyIII expression is restricted to the dorsal ectoderm and ventral SMCs. (B) At early blastula stage sip1 is expressed in two thirds of the embryo. Double in situ hybridization a probe for skeT, a gene expressed in the skeletogenic mesodermal precursors indicates that sip1 is expressed mostly in the presumptive ectoderm derived from the animal hemisphere. lv, lateral view, vv, vegetal pole view. (2.57 MB TIF) [file pgen.1001259.s005.tif]
