# Supplementary material for: Ancestral Regulatory Circuits Governing Ectoderm Patterning Downstream of Nodal and BMP2/4 Revealed by Gene Regulatory Network Analysis in an Echinoderm
Source: PLoS Genet. 2010 Dec 23;6(12):e1001259. doi: 10.1371/journal.pgen.1001259 (PMC3009687; doi:10.1371/journal.pgen.1001259)
Supplement: Table S1 — Gene knockdown and overexpression experiments used to construct the network. (0.07 MB PDF) [file pgen.1001259.s006.pdf]

Interactions deduced from experiments to construct the D/V gene network

A	Perturbed gene INPUT	Spatial domain (*) INPUT	ASSAY	Analyzed gene OUTPUT	Spatial domain (*) OUTPUT	Interaction deduced between INPUT and OUTPUT genes	Summary	Direct interaction ?	Comment	Additional reference on the interaction (*P. lividus*)
	SoxB1	Whole ectoderm	Loss of function	bmp1	Ventral ectoderm + Ciliary band (G, Pr)	Activation	SoxB1 -> bmp1	Unknown		
	SoxB1	Whole ectoderm	Loss of function	chordin	ventral ectoderm (SB->G)	Activation	SoxB1 -> chordin	Unknown		
	SoxB1	Whole ectoderm	Loss of function	deadringer	Ventral ectoderm + Ciliary band (G, Pr)	Activation	SoxB1 -> deadringer	Unknown		
	SoxB1	Whole ectoderm	Loss of function	egip	Ciliary band + Dorsal ectoderm (strong at the apex, weaker elsewhere) (LG, Pr)	Activation	SoxB1 -> egip	Unknown		
	SoxB1	Whole ectoderm	Loss of function	FGFA	Whole ectoderm early, then inhibited dorsally before being restricted to the ciliary band (MB, G, Pr)	Activation	SoxB1 -> FGFA	Unknown		
	SoxB1	Whole ectoderm	Loss of function	foxG	Ventral ectoderm + border with ciliary band - stomodeum (MB, G), then restricted to the ciliary band only (LG/Pr)	Activation	SoxB1 -> foxG	Unknown		
	SoxB1	Whole ectoderm	Loss of function	gfi1	Ciliary band (lateMB, G, Pr)	Activation	SoxB1 -> gfi1	Unknown		
	SoxB1	Whole ectoderm	Loss of function	glypican5	Dorsal ectoderm (MB, G, Pr)	Activation	SoxB1 -> glypican5	Unknown		
	SoxB1	Whole ectoderm	Loss of function	onecut	Whole ectoderm early (blastula), then restricted to the ciliary band (late MB -> Pr)	Activation	SoxB1 -> onecut	Unknown		
	SoxB1	Whole ectoderm	Loss of function	otx	Whole ectoderm early, inhibited in the dorsal most ectoderm (late MB -> Pr), inhibited later in central ventral ectoderm (presumptive stomodeum, LG and Pr stages)	Repression	SoxB1 -> otx	Unknown		
	SoxB1	Whole ectoderm	Loss of function	pax2/5/8	Ciliary band (vegetal part) (G)	Activation	SoxB1 -> pax2/5/8	Unknown		
	SoxB1	Whole ectoderm	Loss of function	univin	Whole ectoderm early, then inhibited dorsally (MB) before being restricted to the lateral part of the ciliary band (G, Pr). At G stage, a weak expression persists in the ventral ectoderm.	Activation	SoxB1 -> univin	Unknown		Range and Lapraz et al., 2008
	SoxB1	Whole ectoderm	Loss of function	wnt8	ventral ectoderm + lateral ectoderm (MB), then restricted to the ciliary band (G, Pr)	Activation	SoxB1 -> wnt8	Unknown		
	Nodal	Ventral ectoderm (64C->G)	Gain of function	29D	dorsal ectoderm (Pr)	Inhibition	Nodal -> 29D	Unknown		Duboc et al, 2004
	Nodal	Ventral ectoderm (64C->G)	Gain of function	admp2	Lower dorsal ectoderm (MB, G)	Inhibition	Nodal -> admp2	Unknown		
	Nodal	Ventral ectoderm (64C->G)	Gain of function	atbf1	Lower ventral + dorsal ectoderm (G)	Inhibition	Nodal -> atbf1	Unknown		
	Nodal	Ventral ectoderm (64C->G)	Gain of function	bmp1	Ventral ectoderm + Ciliary band (G, Pr)	Activation	Nodal -> bmp1	Unknown		
	Nodal	Ventral ectoderm (64C->G)	Gain of function	bmp2/4	ventral ectoderm (EB->G)	Activation	Nodal -> bmp2/4	Unknown		
	Nodal	Ventral ectoderm (64C->G)	Gain of function	brachyury	ventral ectoderm, then stomodeum	Activation	Nodal -> brachyury	Unknown		Duboc et al, 2004
	Nodal	Ventral ectoderm (64C->G)	Gain of function	chordin	ventral ectoderm (SB->G)	Activation	Nodal -> chordin	Unknown		Lapraz et al, 2009
	Nodal	Ventral ectoderm (64C->G)	Gain of function	deadringer	Ventral ectoderm + Ciliary band (G, Pr)	Activation	Nodal -> deadringer	Unknown		
	Nodal	Ventral ectoderm (64C->G)	Gain of function	delta	Neuron precursor (Pr)	Inhibition	Nodal -> delta	Unknown		
	Nodal	Ventral ectoderm (64C->G)	Gain of function	dlx	dorsal ectoderm (MB, G)	Inhibition	Nodal -> dlx	Unknown		
	Nodal	Ventral ectoderm (64C->G)	Gain of function	egip	Ciliary band + Dorsal ectoderm (strong at the apex, weaker elsewhere) (LG, Pr)	Inhibition	Nodal -> egip	Unknown		
	Nodal	Ventral ectoderm (64C->G)	Gain of function	fgfA	Whole ectoderm early, then inhibited dorsally before being restricted to the ciliary band (MB, G, Pr)	Inhibition	Nodal -> fgfA	Unknown		Röttinger et al, 2008
	Nodal	Ventral ectoderm (64C->G)	Gain of function	fgfr1	apical + ventral ectoderm (SB->G)	Activation	Nodal -> fgfr1	Unknown		
	Nodal	Ventral ectoderm (64C->G)	Gain of function	foxA	ventral ectoderm, then stomodeum (MB->Pr)	Activation	Nodal -> foxA	Unknown		
	Nodal	Ventral ectoderm (64C->G)	Gain of function	foxG	Ventral ectoderm + border with ciliary band - stomodeum (MB, G), then restricted to the ciliary band only (LG/Pr)	Activation + Inhibition	Nodal -> foxG	Unknown	induction of ectopic expression in a belt of cells in the animal and vegetal ectoderm inhibition in median territory (presumably corresponding to future [illegible]	
	Nodal	Ventral ectoderm (64C->G)	Gain of function	foxQ2	whole ectoderm early, then restricted to apical domain (B, MB, G)	Independent	N/A	N/A		

A

Perturbed gene INPUT	Spatial domain (*) INPUT	ASSAY	Analyzed gene OUTPUT	Spatial domain (*) OUTPUT	Interaction deduced between INPUT and OUTPUT genes	Summary	Direct interaction ?	Comment	Additional reference on the interaction (*P. lividus*)
Nodal	Ventral ectoderm (64C->G)	Gain of function	gfi1	Ciliary band (lateMB, G, Pr)	Inhibition	Nodal -> gfi1	Unknown		
Nodal	Ventral ectoderm (64C->G)	Gain of function	goosecoid	ventral ectoderm (B, MB), then in a ring around the stomodeum (G)	Activation	Nodal -> goosecoid	Unknown		Duboc et al, 2004
Nodal	Ventral ectoderm (64C->G)	Gain of function	hox7	dorsal ectoderm (MB, G, Pr)	Inhibition	Nodal -> hox7	Unknown		
Nodal	Ventral ectoderm (64C->G)	Gain of function	irxA	Dorsal ectoderm (MB), then upper dorsal ectoderm + stomodeum (G)	Activation + Inhibition	Nodal -> irxA	Unknown	activation : extension of stomodeal territory, inhibition of dorsal expression	
Nodal	Ventral ectoderm (64C->G)	Gain of function	lefty	ventral ectoderm (from early blastula to G)	Activation	Nodal -> lefty	Unknown		Duboc et al, 2004
Nodal	Ventral ectoderm (64C->G)	Gain of function	msx	Dorsal ectoderm (MB), then restricted to lower dorsal ectoderm (G)	Inhibition	Nodal -> msx	Unknown		
Nodal	Ventral ectoderm (64C->G)	Gain of function	nk1	ventral ectoderm (lower half/vegetal)	Activation	Nodal -> nk1	Unknown		
Nodal	Ventral ectoderm (64C->G)	Gain of function	nk2.2	Lower ventral + dorsal ectoderm (LB -> G)	Activation	Nodal -> nk2.2	Unknown		
Nodal	Ventral ectoderm (64C->G)	Gain of function	onecut	Whole ectoderm early (blastula), then restricted to the ciliary band (late MB -> Pr)	Inhibition	Nodal -> onecut	Unknown		
Nodal	Ventral ectoderm (64C->G)	Gain of function	otx	Whole ectoderm early, inhibited in the dorsal most ectoderm (late MB -> Pr), inhibited later in central ventral ectoderm (presumptive stomodeum, LG and Pr stages)	Inhibition	Nodal -> otx	Unknown	at LG stage, Nodal overexpression (high concentration) inhibits Otx expression in a large median ectoderm band that corresponds to the radialized stomodeal territory of these embryos	
Nodal	Ventral ectoderm (64C->G)	Gain of function	pax2/5/8	Ciliary band (vegetal part) (G)	Inhibition	Nodal -> pax2/5/8	Unknown		Röttinger et al, 2008
Nodal	Ventral ectoderm (64C->G)	Gain of function	smad6	Dorsal ectoderm (MB->G)	Inhibition	Nodal -> smad6	Unknown		
Nodal	Ventral ectoderm (64C->G)	Gain of function	tbx2/3	Dorsal ectoderm (LB->G)	Inhibition	Nodal -> tbx2/3	Unknown		Duboc et al, 2004
Nodal	Ventral ectoderm (64C->G)	Gain of function	tubulin	ciliary band (LG and Pr). A weaker expression is present at the dorsal ectoderm apex	Inhibition	Nodal -> tubulin	Unknown		
Nodal	Ventral ectoderm (64C->G)	Gain of function	unc4	Dorsal apex (LG, Pr)	Inhibition	Nodal -> unc4	Unknown		
Nodal	Ventral ectoderm (64C->G)	Gain of function	univin	Whole ectoderm early, then inhibited dorsally (MB) before being restricted to the lateral part of the ciliary band (G, Pr). At G stage, a weak expression persists in the ventral ectoderm.	Activation + Inhibition	Nodal -> univin	Unknown	univin is faintly expressed in the ectoderm in embryos overexpressing nodal at LG stage. This level of expression is compatible with univin weak expression in the ventral ectoderm of control embryos at the same stage. Strong levels of expression found in the ciliary band of control embryos are abolished by nodal overexpression.	
Nodal	Ventral ectoderm (64C->G)	Gain of function	wnt5	Lower dorsal ectoderm (MB, G)	Inhibition	Nodal -> wnt5	Unknown		
Nodal	Ventral ectoderm (64C->G)	Gain of function	wnt8	ventral ectoderm + lateral ectoderm (MB), then restricted to the ciliary band (G, Pr)	Inhibition	Nodal -> wnt8	Unknown		
Lefty	ventral ectoderm (from early blastula to G)	Loss of function	tubulin	ciliary band (LG and Pr). A weaker expression is present at the dorsal ectoderm apex	Inhibition	Lefty -> tubulin	Unknown	Lefty is a Nodal antagonist : in Lefty morphants, Nodal is ectopically expressed in the whole ectoderm	Duboc et al, 2008
Bmp2/4	ventral ectoderm (EB->G)	Gain of function	29D	dorsal ectoderm (Pr)	Activation	Bmp2/4 -> 29D	Unknown		Duboc et al, 2004
Bmp2/4	ventral ectoderm (EB->G)	Gain of function	admp2	Lower dorsal ectoderm (MB, G)	Activation	Bmp2/4 -> admp2	Unknown		
Bmp2/4	ventral ectoderm (EB->G)	Gain of function	atbf1	Lower ventral + dorsal ectoderm (G)	Activation	Bmp2/4 -> atbf1	Unknown		
Bmp2/4	ventral ectoderm (EB->G)	Gain of function	bmp1	Ventral ectoderm + Ciliary band (G, Pr)	Inhibition	Bmp2/4 -> bmp1	Unknown		
Bmp2/4	ventral ectoderm (EB->G)	Gain of function	bmp2/4	ventral ectoderm (EB->G)	Inhibition	Bmp2/4 -> bmp2/4	Unknown		
Bmp2/4	ventral ectoderm (EB->G)	Gain of function	brachyury	ventral ectoderm, then stomodeum	Inhibition	Bmp2/4 -> brachyury	Unknown		Duboc et al, 2004
Bmp2/4	ventral ectoderm (EB->G)	Gain of function	chordin	ventral ectoderm (SB->G)	Inhibition	Bmp2/4 -> chordin	Unknown		
Bmp2/4	ventral ectoderm (EB->G)	Gain of function	deadringer	Ventral ectoderm + Ciliary band (G, Pr)	Inhibition	Bmp2/4 -> deadringer	Unknown		
Bmp2/4	ventral ectoderm (EB->G)	Gain of function	dlx	dorsal ectoderm (MB, G)	Activation	Bmp2/4 -> dlx	Unknown		
Bmp2/4	ventral ectoderm (EB->G)	Gain of function	egip	Ciliary band + Dorsal ectoderm (strong at the apex, weaker elsewhere) (LG, Pr)	Activation + Inhibition	Bmp2/4 -> egip	Unknown	lower dorsal expression (dorsal apex) is extended radially, while the strong ciliary band expression is inhibited if BMP2/4 is overactivated	
Bmp2/4	ventral ectoderm (EB->G)	Gain of function	fgfA	Whole ectoderm early, then inhibited dorsally before being restricted to the ciliary band (MB, G, Pr)	Inhibition	Bmp2/4 -> fgfA	Unknown		
Bmp2/4	ventral ectoderm (EB->G)	Gain of function	foxA	ventral ectoderm, then stomodeum (MB->Pr)	Inhibition	Bmp2/4 -> foxA	Unknown		

A	Perturbed gene INPUT	Spatial domain (*) INPUT	ASSAY	Analyzed gene OUTPUT	Spatial domain (*) OUTPUT	Interaction deduced between INPUT and OUTPUT genes	Summary	Direct interaction ?	Comment	Additional reference on the interaction (*P. lividus*)
	Bmp2/4	ventral ectoderm (EB->G)	Gain of function	foxG	Ventral ectoderm + border with ciliary band - stomodeum (MB, G), then restricted to the ciliary band only (LG/Pr)	Inhibition	Bmp2/4 -> foxG	Unknown		
	Bmp2/4	ventral ectoderm (EB->G)	Gain of function	foxQ2	whole ectoderm early, then restricted to apical domain (B, MB, G)	Inhibition	Bmp2/4 -> foxQ2	Unknown		
	Bmp2/4	ventral ectoderm (EB->G)	Gain of function	gfi1	Ciliary band (lateMB, G, Pr)	Inhibition	Bmp2/4 -> gfi1	Unknown		
	Bmp2/4	ventral ectoderm (EB->G)	Gain of function	goosecoid	ventral ectoderm (B, MB), then in a ring around the stomodeum (G)	Inhibition	Bmp2/4 -> goosecoid	Unknown		Duboc et al, 2004
	Bmp2/4	ventral ectoderm (EB->G)	Gain of function	hox7	dorsal ectoderm (MB, G, Pr)	Activation	Bmp2/4 -> hox7	Unknown		
	Bmp2/4	ventral ectoderm (EB->G)	Gain of function	id	dorsal ectoderm (transient, MB)	Activation	Bmp2/4 -> id	Unknown		
	Bmp2/4	ventral ectoderm (EB->G)	Gain of function	irxA	Dorsal ectoderm (MB), then upper dorsal ectoderm + stomodeum (G)	Activation	Bmp2/4 -> irxA	Unknown		
	Bmp2/4	ventral ectoderm (EB->G)	Gain of function	msx	Dorsal ectoderm (MB), then restricted to lower dorsal ectoderm (G)	Activation	Bmp2/4 -> msx	Unknown		
	Bmp2/4	ventral ectoderm (EB->G)	Gain of function	nk1	ventral ectoderm (lower half/vegetal)	Inhibition	Bmp2/4 -> nk1	Unknown		
	Bmp2/4	ventral ectoderm (EB->G)	Gain of function	nk2.2	Lower ventral + dorsal ectoderm (LB -> G)	Activation	Bmp2/4 -> nk2.2	Unknown		
	Bmp2/4	ventral ectoderm (EB->G)	Gain of function	nodal	Ventral ectoderm (64C->G)	Inhibition	Bmp2/4 -> nodal	Unknown		Duboc et al, 2004
	Bmp2/4	ventral ectoderm (EB->G)	Gain of function	oasis	dorsal ectoderm (MB, G)	Activation	Bmp2/4 -> oasis	Unknown		
	Bmp2/4	ventral ectoderm (EB->G)	Gain of function	onecut	Whole ectoderm early (blastula), then restricted to the ciliary band (late MB -> Pr)	Inhibition	Bmp2/4 -> onecut	Unknown		
	Bmp2/4	ventral ectoderm (EB->G)	Gain of function	otx	Whole ectoderm early, inhibited in the dorsal most ectoderm (late MB -> Pr), inhibited later in central ventral ectoderm (presumptive stomodeum, LG and Pr stages)	Inhibition	Bmp2/4 -> otx	Unknown		
	Bmp2/4	ventral ectoderm (EB->G)	Gain of function	pax2/5/8	Ciliary band (vegetal part) (G)	Inhibition	Bmp2/4 -> pax2/5/8	Unknown		
	Bmp2/4	ventral ectoderm (EB->G)	Gain of function	smad6	Dorsal ectoderm (MB->G)	Activation	Bmp2/4 -> smad6	Unknown		
	Bmp2/4	ventral ectoderm (EB->G)	Gain of function	tbx2/3	Dorsal ectoderm (LB->G)	Activation	Bmp2/4 -> tbx2/3	Unknown		Duboc et al, 2004
	Bmp2/4	ventral ectoderm (EB->G)	Gain of function	tubulin	ciliary band (LG and Pr). A weaker expression is present at the dorsal ectoderm apex	Activation + Inhibition	Bmp2/4 -> tubulin	Unknown	most expression is abolished except a vegetal ring that corresponds to the radialization of the dorsal-most expression of tubulin	
	Bmp2/4	ventral ectoderm (EB->G)	Gain of function	unc4	Dorsal apex (LG, Pr)	Activation	Bmp2/4 -> unc4	Unknown		
	Bmp2/4	ventral ectoderm (EB->G)	Gain of function	univin	Whole ectoderm early, then inhibited dorsally (MB) before being restricted to the lateral part of the ciliary band (G, Pr). At G stage, a weak expression persists in the ventral ectoderm.	Inhibition	Bmp2/4 -> univin	Unknown		
	Bmp2/4	ventral ectoderm (EB->G)	Gain of function	wnt5	Lower dorsal ectoderm (MB, G)	Activation	Bmp2/4 -> wnt5	Unknown		
	Bmp2/4	ventral ectoderm (EB->G)	Gain of function	wnt8	ventral ectoderm + lateral ectoderm (MB), then restricted to the ciliary band (G, Pr)	Inhibition	Bmp2/4 -> wnt8	Unknown		
	Alk3/6 (BMP type I receptor)	Ubiquitous	Gain of function	onecut	Whole ectoderm early (blastula), then restricted to the ciliary band (late MB -> Pr)	Inhibition	Alk3/6 (BMP type I receptor) -> onecut	Unknown	An activated form of the ALK3/6 receptor is used	
	Nodal	Ventral ectoderm (64C->G)	Gain of function+/- translational inhibitor	bmp2/4	ventral ectoderm (EB->G)	Activation	Nodal -> bmp2/4	Yes		
	Nodal	Ventral ectoderm (64C->G)	Gain of function+/- translational inhibitor	brachyury	ventral ectoderm, then stomodeum	Activation	Nodal -> brachyury	No		
	Nodal	Ventral ectoderm (64C->G)	Gain of function+/- translational inhibitor	chordin	ventral ectoderm (SB->G)	Activation	Nodal -> chordin	Yes		
	Nodal	Ventral ectoderm (64C->G)	Gain of function+/- translational inhibitor	deadringer	Ventral ectoderm + Ciliary band (G, Pr)	N/A	N/A	N/A	no response to short treatment with Nodal protein	

A	Perturbed gene INPUT	Spatial domain (*) INPUT	ASSAY	Analyzed gene OUTPUT	Spatial domain (*) OUTPUT	Interaction deduced between INPUT and OUTPUT genes	Summary	Direct interaction ?	Comment	Additional reference on the interaction (*P. lividus*)
	Nodal	Ventral ectoderm (64C->G)	Gain of function+/- translational inhibitor	FGFR1	apical + ventral ectoderm (SB->G)	Activation	Nodal -> FGFR1	Yes		
	Nodal	Ventral ectoderm (64C->G)	Gain of function+/- translational inhibitor	foxA	ventral ectoderm, then stomodeum (MB->Pr)	N/A	N/A	N/A	no response to short treatment with Nodal protein	
	Nodal	Ventral ectoderm (64C->G)	Gain of function+/- translational inhibitor	foxG	Ventral ectoderm + border with ciliary band - stomodeum (MB, G), then restricted to the ciliary band only (LG/Pr)	N/A	N/A	N/A	no response to short treatment with Nodal protein	
	Nodal	Ventral ectoderm (64C->G)	Gain of function+/- translational inhibitor	goosecoid	ventral ectoderm (B, MB), then in a ring around the stomodeum (G)	Activation	Nodal -> goosecoid	Yes		
	Nodal	Ventral ectoderm (64C->G)	Gain of function+/- translational inhibitor	lefty	ventral ectoderm (from early blastula to G)	Activation	Nodal -> lefty	Yes		
	Nodal	Ventral ectoderm (64C->G)	Gain of function+/- translational inhibitor	nk1	ventral ectoderm (lower half/vegetal)	N/A	N/A	N/A	no response to short treatment with Nodal protein	
	Nodal	Ventral ectoderm (64C->G)	Gain of function+/- translational inhibitor	nk2.2	Lower ventral + dorsal ectoderm (LB -> G)	Activation	Nodal -> nk2.2	Yes		
	Nodal	Ventral ectoderm (64C->G)	Gain of function+/- translational inhibitor	nodal	Ventral ectoderm (64C->G)	Activation	Nodal -> nodal	Yes	supported by promoter analysis	
	Nodal	Ventral ectoderm (64C->G)	Gain of function+/- translational inhibitor	tbx2/3	Dorsal ectoderm (LB->G)	N/A	N/A	N/A	no response to short treatment with Nodal protein	
	Bmp2/4	ventral ectoderm (EB->G)	Gain of function+/- translational inhibitor	bmp2/4	ventral ectoderm (EB->G)	N/A	N/A	N/A	no response to short treatment with BMP4 protein	
	Bmp2/4	ventral ectoderm (EB->G)	Gain of function+/- translational inhibitor	dlx	dorsal ectoderm (MB, G)	N/A	N/A	N/A	no response to short treatment with BMP4 protein	
	Bmp2/4	ventral ectoderm (EB->G)	Gain of function+/- translational inhibitor	goosecoid	ventral ectoderm (B, MB), then in a ring around the stomodeum (G)	N/A	N/A	N/A	no response to short treatment with BMP4 protein	
	Bmp2/4	ventral ectoderm (EB->G)	Gain of function+/- translational inhibitor	hox7	dorsal ectoderm (MB, G, Pr)	N/A	N/A	N/A	no response to short treatment with BMP4 protein	
	Bmp2/4	ventral ectoderm (EB->G)	Gain of function+/- translational inhibitor	irxA	Dorsal ectoderm (MB), then upper dorsal ectoderm + stomodeum (G)	N/A	N/A	N/A	no response to short treatment with BMP4 protein	
	Bmp2/4	ventral ectoderm (EB->G)	Gain of function+/- translational inhibitor	msx	Dorsal ectoderm (MB), then restricted to lower dorsal ectoderm (G)	N/A	N/A	N/A	no response to short treatment with BMP4 protein	
	Bmp2/4	ventral ectoderm (EB->G)	Gain of function+/- translational inhibitor	nk2.2	Lower ventral + dorsal ectoderm (LB -> G)	Activation	Bmp2/4 -> nk2.2	Yes		
	Bmp2/4	ventral ectoderm (EB->G)	Gain of function+/- translational inhibitor	onecut	Whole ectoderm early (blastula), then restricted to the ciliary band (late MB -> Pr)	N/A	N/A	N/A	no response to short treatment with BMP4 protein	
	Bmp2/4	ventral ectoderm (EB->G)	Gain of function+/- translational inhibitor	pax2/5/8	Ciliary band (vegetal part) (G)	N/A	N/A	N/A	no response to short treatment with BMP4 protein	
	Bmp2/4	ventral ectoderm (EB->G)	Gain of function+/- translational inhibitor	smad6	Dorsal ectoderm (MB->G)	Activation	Bmp2/4 -> smad6	Yes		
	Bmp2/4	ventral ectoderm (EB->G)	Gain of function+/- translational inhibitor	tbx2/3	Dorsal ectoderm (LB->G)	Activation	Bmp2/4 -> tbx2/3	Yes		
	Bmp2/4	ventral ectoderm (EB->G)	Gain of function+/- translational inhibitor	wnt5	Lower dorsal ectoderm (MB, G)	N/A	N/A	N/A	no response to short treatment with BMP4 protein	

A	Perturbed gene INPUT	Spatial domain (*) INPUT	ASSAY	Analyzed gene OUTPUT	Spatial domain (*) OUTPUT	Interaction deduced between INPUT and OUTPUT genes	Summary	Direct interaction ?	Comment	Additional reference on the interaction (*P. lividus*)
	Nodal	Ventral ectoderm (64C->G)	Loss of function	29D	dorsal ectoderm (Pr)	Activation	Nodal -> 29D	No	see Bmp2/4 loss of function + previous data indicating that Bmp2/4 is strictly required for dorsal ectoderm patterning (Lapraz et al, 2009)	Duboc et al, 2004
	Nodal	Ventral ectoderm (64C->G)	Loss of function	atbf1	Lower ventral + dorsal ectoderm (G)	Activation	Nodal -> atbf1	No	see Bmp2/4 loss of function + previous data indicating that Bmp2/4 is strictly required for dorsal ectoderm patterning (Lapraz et al, 2009)	
	Nodal	Ventral ectoderm (64C->G)	Loss of function	bmp1	Ventral ectoderm + Ciliary band (G, Pr)	Inhibition	Nodal -> bmp1	Unknown		
	Nodal	Ventral ectoderm (64C->G)	Loss of function	bmp2/4	ventral ectoderm (EB->G)	Activation	Nodal -> bmp2/4	Yes	see inductive assay in presence of translational inhibitor	Duboc et al, 2004
	Nodal	Ventral ectoderm (64C->G)	Loss of function	brachyury	ventral ectoderm, then stomodeum	Activation	Nodal -> brachyury	No	see inductive assay in presence of translational inhibitor	Duboc et al, 2004
	Nodal	Ventral ectoderm (64C->G)	Loss of function	chordin	ventral ectoderm (SB->G)	Activation	Nodal -> chordin	Yes	see inductive assay in presence of translational inhibitor	Lapraz et al, 2009
	Nodal	Ventral ectoderm (64C->G)	Loss of function	deadringer	Ventral ectoderm + Ciliary band (G, Pr)	Inhibition	Nodal -> deadringer	Unknown		
	Nodal	Ventral ectoderm (64C->G)	Loss of function	delta	Neuron precursor (Pr)	Inhibition	Nodal -> delta	Unknown		
	Nodal	Ventral ectoderm (64C->G)	Loss of function	dlx	dorsal ectoderm (MB, G)	Activation	Nodal -> dlx	No	see Bmp2/4 loss of function + previous data indicating that Bmp2/4 is strictly required for dorsal ectoderm patterning (Lapraz et al, 2009)	
	Nodal	Ventral ectoderm (64C->G)	Loss of function	fgfA	Whole ectoderm early, then inhibited dorsally before being restricted to the ciliary band (MB, G, Pr)	Inhibition	Nodal -> fgfA	Unknown		Röttinger et al, 2008
	Nodal	Ventral ectoderm (64C->G)	Loss of function	fgfr1	apical + ventral ectoderm (SB->G)	Activation	Nodal -> fgfr1	Unknown		
	Nodal	Ventral ectoderm (64C->G)	Loss of function	foxA	ventral ectoderm, then stomodeum (MB->Pr)	Activation	Nodal -> foxA	Unknown		
	Nodal	Ventral ectoderm (64C->G)	Loss of function	foxG	Ventral ectoderm + border with ciliary band - stomodeum (MB, G), then restricted to the ciliary band only (LG/Pr)	Activation	Nodal -> foxG	Unknown	complete loss of expression before LG/Pr stage, when foxG is mainly in the ventral ectoderm	
	Nodal	Ventral ectoderm (64C->G)	Loss of function	gfi1	Ciliary band (lateMB, G, Pr)	Inhibition	Nodal -> gfi1	Unknown		
	Nodal	Ventral ectoderm (64C->G)	Loss of function	glypican5	Dorsal ectoderm (MB, G, Pr)	Activation	Nodal -> glypican5	No	see Bmp2/4 loss of function + previous data indicating that Bmp2/4 is strictly required for dorsal ectoderm patterning (Lapraz et al, 2009)	
	Nodal	Ventral ectoderm (64C->G)	Loss of function	goosecoid	ventral ectoderm (B, MB), then in a ring around the stomodeum (G)	Activation	Nodal -> goosecoid	Yes	see inductive assay in presence of translational inhibitor	Duboc et al, 2004
	Nodal	Ventral ectoderm (64C->G)	Loss of function	hox7	dorsal ectoderm (MB, G, Pr)	Activation	Nodal -> hox7	No	see Bmp2/4 loss of function + previous data indicating that Bmp2/4 is strictly required for dorsal ectoderm patterning (Lapraz et al, 2009)	
	Nodal	Ventral ectoderm (64C->G)	Loss of function	id	dorsal ectoderm (transient, MB)	Activation	Nodal -> id	No	see Bmp2/4 loss of function + previous data indicating that Bmp2/4 is strictly required for dorsal ectoderm patterning (Lapraz et al, 2009)	
	Nodal	Ventral ectoderm (64C->G)	Loss of function	irxA	Dorsal ectoderm (MB), then upper dorsal ectoderm + stomodeum (G)	Activation	Nodal -> irxA	No	see Bmp2/4 loss of function + previous data indicating that Bmp2/4 is strictly required for dorsal ectoderm patterning (Lapraz et al, 2009)	
	Nodal	Ventral ectoderm (64C->G)	Loss of function	lefty	ventral ectoderm (from early blastula to G)	Activation	Nodal -> lefty	Yes	see inductive assay in presence of translational inhibitor	Duboc et al, 2004
	Nodal	Ventral ectoderm (64C->G)	Loss of function	nk2.2	Lower ventral + dorsal ectoderm (LB -> G)	Activation	Nodal -> nk2.2	Yes	for the ventral part of nk2.2 expression<br>see inductive assay in presence of translational inhibitor	
	Nodal	Ventral ectoderm (64C->G)	Loss of function	oasis	dorsal ectoderm (MB, G)	Activation	Nodal -> oasis	No	see Bmp2/4 loss of function + previous data indicating that Bmp2/4 is strictly required for dorsal ectoderm patterning (Lapraz et al, 2009)	
	Nodal	Ventral ectoderm (64C->G)	Loss of function	onecut	Whole ectoderm early (blastula), then restricted to the ciliary band (late MB -> Pr)	Inhibition	Nodal -> onecut	Unknown		
	Nodal	Ventral ectoderm (64C->G)	Loss of function	otx	Whole ectoderm early,<br>inhibited in the dorsal most ectoderm (late MB -> Pr), inhibited later in central ventral ectoderm (presumptive stomodeum, LG and Pr stages)	Inhibition	Nodal -> otx	No	the inhibition occurs in the dorsal ectoderm at the G stage.<br>see Bmp2/4 loss of function + previous data indicating that Bmp2/4 is strictly required for dorsal ectoderm patterning	
	Nodal	Ventral ectoderm (64C->G)	Loss of function	pax2/5/8	Ciliary band (vegetal part) (G)	Inhibition	Nodal -> pax2/5/8	No	pax2/5/8 is a target of FGFA signaling	Röttinger et al, 2008

A Perturbed gene INPUT	Spatial domain (*) INPUT	ASSAY	Analyzed gene OUTPUT	Spatial domain (*) OUTPUT	Interaction deduced between INPUT and OUTPUT genes	Summary	Direct interaction ?	Comment	Additional reference on the interaction (*P. lividus*)
Nodal	Ventral ectoderm (64C->G)	Loss of function	smad6	Dorsal ectoderm (MB->G)	Activation	Nodal -> smad6	No	see Bmp2/4 loss of function + previous data indicating that Bmp2/4 is strictly required for dorsal ectoderm patterning (Lapraz et al, 2009)	
Nodal	Ventral ectoderm (64C->G)	Loss of function	tbx2/3	Dorsal ectoderm (LB->G)	Activation	Nodal -> tbx2/3	No	see Bmp2/4 loss of function + previous data indicating that Bmp2/4 is strictly required for dorsal ectoderm patterning (Lapraz et al, 2009)	Duboc et al, 2004
Nodal	Ventral ectoderm (64C->G)	Loss of function	tubulin	ciliary band (LG and Pr). A weaker expression is present at the dorsal ectoderm apex	Inhibition	Nodal -> tubulin	Unknown		Duboc et al, 2004
Nodal	Ventral ectoderm (64C->G)	Loss of function	unc4	Dorsal apex (LG, Pr)	Activation	Nodal -> unc4	No	see Bmp2/4 loss of function + previous data indicating that Bmp2/4 is strictly required for dorsal ectoderm patterning (Lapraz et al, 2009)	
Nodal	Ventral ectoderm (64C->G)	Loss of function	univin	Whole ectoderm early, then inhibited dorsally (MB) before being restricted to the lateral part of the ciliary band (G, Pr). At G stage, a weak expression persists in the ventral ectoderm.	Inhibition	Nodal -> univin	Unknown		
Nodal	Ventral ectoderm (64C->G)	Loss of function	wnt5	Lower dorsal ectoderm (MB, G)	Activation	Nodal -> wnt5	No	see Bmp2/4 loss of function + previous data indicating that Bmp2/4 is strictly required for dorsal ectoderm patterning (Lapraz et al, 2009)	
Nodal	Ventral ectoderm (64C->G)	Loss of function	wnt8	ventral ectoderm + lateral ectoderm (MB), then restricted to the ciliary band (G, Pr)	Inhibition	Nodal -> wnt8	Unknown		
Alk4/5/7 (Nodal type I receptor)	Ubiquitous	Loss of function	admp2	Lower dorsal ectoderm (MB, G)	Activation	Alk4/5/7 (Nodal type I receptor) -> admp2	No	see Bmp2/4 loss of function + previous data indicating that Bmp2/4 is strictly required for dorsal ectoderm patterning (Lapraz et al, 2009)	
Alk4/5/7 (Nodal type I receptor)	Ubiquitous	Loss of function	atbf1	Lower ventral + dorsal ectoderm (G)	Activation	Alk4/5/7 (Nodal type I receptor) -> atbf1	No	see Bmp2/4 loss of function + previous data indicating that Bmp2/4 is strictly required for dorsal ectoderm patterning (Lapraz et al, 2009)	
Alk4/5/7 (Nodal type I receptor)	Ubiquitous	Loss of function	bmp1	Ventral ectoderm + Ciliary band (G, Pr)	Inhibition	Alk4/5/7 (Nodal type I receptor) -> bmp1	Unknown		
Alk4/5/7 (Nodal type I receptor)	Ubiquitous	Loss of function	bmp2/4	ventral ectoderm (EB->G)	Activation	Alk4/5/7 (Nodal type I receptor) -> bmp2/4	Yes	see inductive assay in presence of translational inhibitor	
Alk4/5/7 (Nodal type I receptor)	Ubiquitous	Loss of function	brachyury	ventral ectoderm, then stomodeum	Activation	Alk4/5/7 (Nodal type I receptor) -> brachyury	No	see inductive assay in presence of translational inhibitor	
Alk4/5/7 (Nodal type I receptor)	Ubiquitous	Loss of function	chordin	ventral ectoderm (SB->G)	Activation	Alk4/5/7 (Nodal type I receptor) -> chordin	Yes	see inductive assay in presence of translational inhibitor	Lapraz et al, 2009
Alk4/5/7 (Nodal type I receptor)	Ubiquitous	Loss of function	deadringer	Ventral ectoderm + Ciliary band (G, Pr)	Inhibition	Alk4/5/7 (Nodal type I receptor) -> deadringer	Unknown		
Alk4/5/7 (Nodal type I receptor)	Ubiquitous	Loss of function	delta	Neuron precursor (Pr)	Inhibition	Alk4/5/7 (Nodal type I receptor) -> delta	Unknown		
Alk4/5/7 (Nodal type I receptor)	Ubiquitous	Loss of function	fgfA	Whole ectoderm early, then inhibited dorsally before being restricted to the ciliary band (MB, G, Pr)	Inhibition	Alk4/5/7 (Nodal type I receptor) -> fgfA	Unknown		
Alk4/5/7 (Nodal type I receptor)	Ubiquitous	Loss of function	foxA	ventral ectoderm, then stomodeum (MB->Pr)	activation	Alk4/5/7 (Nodal type I receptor) -> foxA	Unknown		
Alk4/5/7 (Nodal type I receptor)	Ubiquitous	Loss of function	foxG	Ventral ectoderm + border with ciliary band - stomodeum (MB, G), then restricted to the ciliary band only (LG/Pr)	activation	Alk4/5/7 (Nodal type I receptor) -> foxG	Unknown		
Alk4/5/7 (Nodal type I receptor)	Ubiquitous	Loss of function	foxQ2	whole ectoderm early, then restricted to apical domain (B, MB, G)	Independent	N/A	N/A		
Alk4/5/7 (Nodal type I receptor)	Ubiquitous	Loss of function	gfi1	Ciliary band (lateMB, G, Pr)	Inhibition	Alk4/5/7 (Nodal type I receptor) -> gfi1	Unknown		

A	Perturbed gene INPUT	Spatial domain (*) INPUT	ASSAY	Analyzed gene OUTPUT	Spatial domain (*) OUTPUT	Interaction deduced between INPUT and OUTPUT genes	Summary	Direct interaction ?	Comment	Additional reference on the interaction (*P. lividus*)
	Alk4/5/7 (Nodal type I receptor)	Ubiquitous	Loss of function	goosecoid	ventral ectoderm (B, MB), then in a ring around the stomodeum (G)	activation	Alk4/5/7 (Nodal type I receptor) -> goosecoid	Unknown		
	Alk4/5/7 (Nodal type I receptor)	Ubiquitous	Loss of function	hox7	dorsal ectoderm (MB, G, Pr)	activation	Alk4/5/7 (Nodal type I receptor) -> hox7	No	see Bmp2/4 loss of function + previous data indicating that Bmp2/4 is strictly required for dorsal ectoderm patterning (Lapraz et al, 2009)	
	Alk4/5/7 (Nodal type I receptor)	Ubiquitous	Loss of function	irxA	Dorsal ectoderm (MB), then upper dorsal ectoderm + stomodeum (G)	activation	Alk4/5/7 (Nodal type I receptor) -> irxA	Unknown		
	Alk4/5/7 (Nodal type I receptor)	Ubiquitous	Loss of function	lefty	ventral ectoderm (from early blastula to G)	activation	Alk4/5/7 (Nodal type I receptor) -> lefty	Yes	see inductive assay in presence of translational inhibitor	
	Alk4/5/7 (Nodal type I receptor)	Ubiquitous	Loss of function	msx	Dorsal ectoderm (MB), then restricted to lower dorsal ectoderm (G)	activation	Alk4/5/7 (Nodal type I receptor) -> msx	No	see Bmp2/4 loss of function + previous data indicating that Bmp2/4 is strictly required for dorsal ectoderm patterning (Lapraz et al, 2009)	
	Alk4/5/7 (Nodal type I receptor)	Ubiquitous	Loss of function	nk1	ventral ectoderm (lower half/vegetal)	activation	Alk4/5/7 (Nodal type I receptor) -> nk1	Unknown		
	Alk4/5/7 (Nodal type I receptor)	Ubiquitous	Loss of function	nk2.2	Lower ventral + dorsal ectoderm (LB -> G)	activation	Alk4/5/7 (Nodal type I receptor) -> nk2.2	Yes	for the ventral part of nk2.2 expression see inductive assay in presence of translational inhibitor	
	Alk4/5/7 (Nodal type I receptor)	Ubiquitous	Loss of function	onecut	Whole ectoderm early (blastula), then restricted to the ciliary band (late MB -> Pr)	Inhibition	Alk4/5/7 (Nodal type I receptor) -> onecut	Unknown		
	Alk4/5/7 (Nodal type I receptor)	Ubiquitous	Loss of function	otx	Whole ectoderm early, inhibited in the dorsal most ectoderm (late MB -> Pr), inhibited later in central ventral ectoderm (presumptive stomodeum, LG and Pr stages)	Inhibition	Alk4/5/7 (Nodal type I receptor) -> otx	Unknown		
	Alk4/5/7 (Nodal type I receptor)	Ubiquitous	Loss of function	pax2/5/8	Ciliary band (vegetal part) (G)	Inhibition	Alk4/5/7 (Nodal type I receptor) -> pax2/5/8	Unknown		
	Alk4/5/7 (Nodal type I receptor)	Ubiquitous	Loss of function	smad6	Dorsal ectoderm (MB->G)	activation	Alk4/5/7 (Nodal type I receptor) -> smad6	Unknown		
	Alk4/5/7 (Nodal type I receptor)	Ubiquitous	Loss of function	tbx2/3	Dorsal ectoderm (LB->G)	activation	Alk4/5/7 (Nodal type I receptor) -> tbx2/3	Unknown		
	Alk4/5/7 (Nodal type I receptor)	Ubiquitous	Loss of function	unc4	Dorsal apex (LG, Pr)	activation	Alk4/5/7 (Nodal type I receptor) -> unc4	Unknown		
	Alk4/5/7 (Nodal type I receptor)	Ubiquitous	Loss of function	univin	Whole ectoderm early, then inhibited dorsally (MB) before being restricted to the lateral part of the ciliary band (G, Pr). At G stage, a weak expression persists in the ventral ectoderm.	Inhibition	Alk4/5/7 (Nodal type I receptor) -> univin	Unknown		
	Alk4/5/7 (Nodal type I receptor)	Ubiquitous	Loss of function	wnt5	Lower dorsal ectoderm (MB, G)	activation	Alk4/5/7 (Nodal type I receptor) -> wnt5	Unknown		
	Alk4/5/7 (Nodal type I receptor)	Ubiquitous	Loss of function	wnt8	ventral ectoderm + lateral ectoderm (MB), then restricted to the ciliary band (G, Pr)	Inhibition	Alk4/5/7 (Nodal type I receptor) -> wnt8	Unknown		
	Bmp2/4	ventral ectoderm (EB->G)	Loss of function	29D	dorsal ectoderm (Pr)	Activation	Bmp2/4 -> 29D	Unknown		Duboc et al, 2004
	Bmp2/4	ventral ectoderm (EB->G)	Loss of function	atbf1	Lower ventral + dorsal ectoderm (G)	Activation	Bmp2/4 -> atbf1	Unknown		
	Bmp2/4	ventral ectoderm (EB->G)	Loss of function	deadringer	Ventral ectoderm + Ciliary band (G, Pr)	Inhibition	Bmp2/4 -> deadringer	Unknown		
	Bmp2/4	ventral ectoderm (EB->G)	Loss of function	fgfA	Whole ectoderm early, then inhibited dorsally before being restricted to the ciliary band (MB, G, Pr)	Inhibition	Bmp2/4 -> fgfA	Unknown		
	Bmp2/4	ventral ectoderm (EB->G)	Loss of function	foxG	Ventral ectoderm + border with ciliary band - stomodeum (MB, G), then restricted to the ciliary band only (LG/Pr)	Inhibition	Bmp2/4 -> foxG	Unknown		
	Bmp2/4	ventral ectoderm (EB->G)	Loss of function	gfi1	Ciliary band (lateMB, G, Pr)	Inhibition	Bmp2/4 -> gfi1	Unknown		

A Perturbed gene INPUT	Spatial domain (*) INPUT	ASSAY	Analyzed gene OUTPUT	Spatial domain (*) OUTPUT	Interaction deduced between INPUT and OUTPUT genes	Summary	Direct interaction ?	Comment	Additional reference on the interaction (*P. lividus*)
Bmp2/4	ventral ectoderm (EB->G)	Loss of function	hox7	dorsal ectoderm (MB, G, Pr)	Activation	Bmp2/4 -> hox7	Unknown		
Bmp2/4	ventral ectoderm (EB->G)	Loss of function	irxA	Dorsal ectoderm (MB), then upper dorsal ectoderm + stomodeum (G)	Activation	Bmp2/4 -> irxA	Unknown		
Bmp2/4	ventral ectoderm (EB->G)	Loss of function	nk2.2	Lower ventral + dorsal ectoderm (LB -> G)	Activation	Bmp2/4 -> nk2.2	Yes	see inductive assay in presence of translational inhibitor	
Bmp2/4	ventral ectoderm (EB->G)	Loss of function	otx	Whole ectoderm early, inhibited in the dorsal most ectoderm (late MB -> Pr), inhibited later in central ventral ectoderm (presumptive stomodeum, LG and Pr stages)	Inhibition	Bmp2/4 -> otx	Unknown		
Bmp2/4	ventral ectoderm (EB->G)	Loss of function	pax2/5/8	Ciliary band (vegetal part) (G)	Inhibition	Bmp2/4 -> pax2/5/8	Unknown		
Bmp2/4	ventral ectoderm (EB->G)	Loss of function	smad6	Dorsal ectoderm (MB->G)	Activation	Bmp2/4 -> smad6	Yes	see inductive assay in presence of translational inhibitor	
Bmp2/4	ventral ectoderm (EB->G)	Loss of function	tbx2/3	Dorsal ectoderm (LB->G)	Activation	Bmp2/4 -> tbx2/3	Yes	see inductive assay in presence of translational inhibitor	Duboc et al, 2004<br>Lapraz et al, 2009
Bmp2/4	ventral ectoderm (EB->G)	Loss of function	unc4	Dorsal apex (LG, Pr)	Activation	Bmp2/4 -> unc4	Unknown		
Bmp2/4	ventral ectoderm (EB->G)	Loss of function	univin	Whole ectoderm early, then inhibited dorsally (MB) before being restricted to the lateral part of the ciliary band (G, Pr). At G stage, a weak expression persists in the ventral ectoderm.	Inhibition	Bmp2/4 -> univin	Unknown		
Bmp2/4	ventral ectoderm (EB->G)	Loss of function	wnt5	Lower dorsal ectoderm (MB, G)	Activation	Bmp2/4 -> wnt5	Unknown		
Bmp2/4	ventral ectoderm (EB->G)	Loss of function	wnt8	ventral ectoderm + lateral ectoderm (MB), then restricted to the ciliary band (G, Pr)	Inhibition	Bmp2/4 -> wnt8	Unknown		
Alk3/6 (BMP type I receptor)	Ubiquitous	Loss of function	29D	dorsal ectoderm (Pr)	Activation	Alk3/6 (BMP type I receptor) -> 29D	Unknown		
Alk3/6 (BMP type I receptor)	Ubiquitous	Loss of function	bmp1	Ventral ectoderm + Ciliary band (G, Pr)	Inhibition	Alk3/6 (BMP type I receptor) -> bmp1	Unknown		
Alk3/6 (BMP type I receptor)	Ubiquitous	Loss of function	bmp2/4	ventral ectoderm (EB->G)	Independent	N/A	N/A		
Alk3/6 (BMP type I receptor)	Ubiquitous	Loss of function	brachyury	ventral ectoderm, then stomodeum	Independent	N/A	N/A		
Alk3/6 (BMP type I receptor)	Ubiquitous	Loss of function	chordin	ventral ectoderm (SB->G)	Independent	N/A	N/A		
Alk3/6 (BMP type I receptor)	Ubiquitous	Loss of function	dlx	dorsal ectoderm (MB, G)	Activation	Alk3/6 (BMP type I receptor) -> dlx	Unknown		
Alk3/6 (BMP type I receptor)	Ubiquitous	Loss of function	fgfA	Whole ectoderm early, then inhibited dorsally before being restricted to the ciliary band (MB, G, Pr)	Inhibition	Alk3/6 (BMP type I receptor) -> fgfA	Unknown		
Alk3/6 (BMP type I receptor)	Ubiquitous	Loss of function	fgfr1	apical + ventral ectoderm (SB->G)	Independent	N/A	N/A	not shown	
Alk3/6 (BMP type I receptor)	Ubiquitous	Loss of function	foxA	ventral ectoderm, then stomodeum (MB->Pr)	Independent	N/A	N/A		
Alk3/6 (BMP type I receptor)	Ubiquitous	Loss of function	foxG	Ventral ectoderm + border with ciliary band - stomodeum (MB, G), then restricted to the ciliary band only (LG/Pr)	Inhibition	Alk3/6 (BMP type I receptor) -> foxG	Unknown		
Alk3/6 (BMP type I receptor)	Ubiquitous	Loss of function	gfi1	Ciliary band (lateMB, G, Pr)	Inhibition	Alk3/6 (BMP type I receptor) -> gfi1	Unknown		

A

Perturbed gene INPUT	Spatial domain (*) INPUT	ASSAY	Analyzed gene OUTPUT	Spatial domain (*) OUTPUT	Interaction deduced between INPUT and OUTPUT genes	Summary	Direct interaction ?	Comment	Additional reference on the interaction (*P. lividus*)
Alk3/6 (BMP type I receptor)	Ubiquitous	Loss of function	glypican5	Dorsal ectoderm (MB, G, Pr)	Activation	Alk3/6 (BMP type I receptor) -> glypican5	Unknown	lapraz et al, 2009	
Alk3/6 (BMP type I receptor)	Ubiquitous	Loss of function	id	dorsal ectoderm (transient, MB)	Activation	Alk3/6 (BMP type I receptor) -> id	Unknown		
Alk3/6 (BMP type I receptor)	Ubiquitous	Loss of function	irxA	Dorsal ectoderm (MB), then upper dorsal ectoderm + stomodeum (G)	Activation	Alk3/6 (BMP type I receptor) -> irxA	Unknown		
Alk3/6 (BMP type I receptor)	Ubiquitous	Loss of function	msx	Dorsal ectoderm (MB), then restricted to lower dorsal ectoderm (G)	Activation	Alk3/6 (BMP type I receptor) -> msx	Unknown		
Alk3/6 (BMP type I receptor)	Ubiquitous	Loss of function	nk2.2	Lower ventral + dorsal ectoderm (LB -> G)	Activation	Alk3/6 (BMP type I receptor) -> nk2.2	Yes	see inductive assay in presence of translational inhibitor	
Alk3/6 (BMP type I receptor)	Ubiquitous	Loss of function	nodal	Ventral ectoderm (64C->G)	Independent	N/A	N/A		
Alk3/6 (BMP type I receptor)	Ubiquitous	Loss of function	onecut	Whole ectoderm early (blastula), then restricted to the ciliary band (late MB -> Pr)	Inhibition	Alk3/6 (BMP type I receptor) -> onecut	Unknown		Lapraz et al, 2009
Alk3/6 (BMP type I receptor)	Ubiquitous	Loss of function	otx	Whole ectoderm early,<br>inhibited in the dorsal most ectoderm (late MB -> Pr), inhibited later in central ventral ectoderm (presumptive stomodeum, LG and Pr stages)	Inhibition	Alk3/6 (BMP type I receptor) -> otx	Unknown		
Alk3/6 (BMP type I receptor)	Ubiquitous	Loss of function	pax2/5/8	Ciliary band (vegetal part) (G)	Inhibition	Alk3/6 (BMP type I receptor) -> pax2/5/8	Unknown		
Alk3/6 (BMP type I receptor)	Ubiquitous	Loss of function	smad6	Dorsal ectoderm (MB->G)	Activation	Alk3/6 (BMP type I receptor) -> smad6	Yes	see inductive assay in presence of translational inhibitor	
Alk3/6 (BMP type I receptor)	Ubiquitous	Loss of function	tbx2/3	Dorsal ectoderm (LB->G)	Activation	Alk3/6 (BMP type I receptor) -> tbx2/3	Yes	see inductive assay in presence of translational inhibitor	Lapraz et al, 2009
Alk3/6 (BMP type I receptor)	Ubiquitous	Loss of function	tubulin	ciliary band (LG and Pr). A weaker expression is present at the dorsal ectoderm apex	Activation	Alk3/6 (BMP type I receptor) -> tubulin	Unknown		
Alk3/6 (BMP type I receptor)	Ubiquitous	Loss of function	univin	Whole ectoderm early, then inhibited dorsally (MB) before being restricted to the lateral part of the ciliary band (G, Pr). At G stage, a weak expression persists in the ventral ectoderm.	Inhibition	Alk3/6 (BMP type I receptor) -> univin	Unknown		
Alk3/6 (BMP type I receptor)	Ubiquitous	Loss of function	wnt5	Lower dorsal ectoderm (MB, G)	Activation	Alk3/6 (BMP type I receptor) -> wnt5	Unknown		
Alk3/6 (BMP type I receptor)	Ubiquitous	Loss of function	wnt8	ventral ectoderm + lateral ectoderm (MB), then restricted to the ciliary band (G, Pr)	Inhibition	Alk3/6 (BMP type I receptor) -> wnt8	Unknown		
Goosecoid	ventral ectoderm (B, MB), then in a ring around the stomodeum (G)	Loss of function	bmp1	Ventral ectoderm + Ciliary band (G, Pr)	Independent	N/A	N/A	not shown	
Goosecoid	ventral ectoderm (B, MB), then in a ring around the stomodeum (G)	Loss of function	bmp2/4	ventral ectoderm (EB->G)	Independent	N/A	N/A		
Goosecoid	ventral ectoderm (B, MB), then in a ring around the stomodeum (G)	Loss of function	brachyury	ventral ectoderm, then stomodeum	Activation	Goosecoid -> brachyury	Unknown		
Goosecoid	ventral ectoderm (B, MB), then in a ring around the stomodeum (G)	Loss of function	chordin	ventral ectoderm (SB->G)	Independent	N/A	N/A		
Goosecoid	ventral ectoderm (B, MB), then in a ring around the stomodeum (G)	Loss of function	foxA	ventral ectoderm, then stomodeum (MB->Pr)	Activation	Goosecoid -> foxA	Unknown		

A	Perturbed gene INPUT	Spatial domain (*) INPUT	ASSAY	Analyzed gene OUTPUT	Spatial domain (*) OUTPUT	Interaction deduced between INPUT and OUTPUT genes	Summary	Direct interaction ?	Comment	Additional reference on the interaction (*P. lividus*)
	Goosecoid	ventral ectoderm (B, MB), then in a ring around the stomodeum (G)	Loss of function	foxG	Ventral ectoderm + border with ciliary band - stomodeum (MB, G), then restricted to the ciliary band only (LG/Pr)	Inhibition	Goosecoid -> foxG	Unknown		
	Goosecoid	ventral ectoderm (B, MB), then in a ring around the stomodeum (G)	Loss of function	gfi1	Ciliary band (lateMB, G, Pr)	Independent	N/A	N/A	not shown	
	Goosecoid	ventral ectoderm (B, MB), then in a ring around the stomodeum (G)	Loss of function	hox7	dorsal ectoderm (MB, G, Pr)	Independent	N/A	N/A	not shown	
	Goosecoid	ventral ectoderm (B, MB), then in a ring around the stomodeum (G)	Loss of function	msx	Dorsal ectoderm (MB), then restricted to lower dorsal ectoderm (G)	Independent	N/A	N/A	not shown	
	Goosecoid	ventral ectoderm (B, MB), then in a ring around the stomodeum (G)	Loss of function	otx	Whole ectoderm early,<br>inhibited in the dorsal most ectoderm (late MB -> Pr), inhibited later in central ventral ectoderm (presumptive stomodeum, LG and Pr stages)	Independent	N/A	N/A	not shown	
	Goosecoid	ventral ectoderm (B, MB), then in a ring around the stomodeum (G)	Loss of function	tbx2/3	Dorsal ectoderm (LB->G)	Independent	N/A	N/A	not shown	
	Goosecoid	ventral ectoderm (B, MB), then in a ring around the stomodeum (G)	Loss of function	univin	Whole ectoderm early, then inhibited dorsally (MB) before being restricted to the lateral part of the ciliary band (G, Pr). At G stage, a weak expression persists in the ventral ectoderm.	Inhibition	Goosecoid -> univin	Unknown		
	Goosecoid	ventral ectoderm (B, MB), then in a ring around the stomodeum (G)	Loss of function	wnt8	ventral ectoderm + lateral ectoderm (MB), then restricted to the ciliary band (G, Pr)	Inhibition	Goosecoid -> wnt8	Unknown		
	Goosecoid	ventral ectoderm (B, MB), then in a ring around the stomodeum (G)	Gain of function	brachyury	ventral ectoderm, then stomodeum	Activation	Goosecoid -> brachyury	Unknown		
	Goosecoid	ventral ectoderm (B, MB), then in a ring around the stomodeum (G)	Gain of function	deadringer	Ventral ectoderm + Ciliary band (G, Pr)	Activation	Goosecoid -> deadringer	Unknown		
	Goosecoid	ventral ectoderm (B, MB), then in a ring around the stomodeum (G)	Gain of function	egip	Ciliary band + Dorsal ectoderm (strong at the apex, weaker elsewhere) (LG, Pr)	Inhibition	Goosecoid -> egip	Unknown		
	Goosecoid	ventral ectoderm (B, MB), then in a ring around the stomodeum (G)	Gain of function	foxA	ventral ectoderm, then stomodeum (MB->Pr)	Activation	Goosecoid -> foxA	Unknown		
	Goosecoid	ventral ectoderm (B, MB), then in a ring around the stomodeum (G)	Gain of function	foxG	Ventral ectoderm + border with ciliary band - stomodeum (MB, G), then restricted to the ciliary band only (LG/Pr)	Activation	Goosecoid -> foxG	Unknown		
	Goosecoid	ventral ectoderm (B, MB), then in a ring around the stomodeum (G)	Gain of function	gfi1	Ciliary band (lateMB, G, Pr)	Inhibition	Goosecoid -> gfi1	Unknown		
	Goosecoid	ventral ectoderm (B, MB), then in a ring around the stomodeum (G)	Gain of function	hox7	dorsal ectoderm (MB, G, Pr)	Inhibition	Goosecoid -> hox7	Unknown		
	Goosecoid	ventral ectoderm (B, MB), then in a ring around the stomodeum (G)	Gain of function	msx	Dorsal ectoderm (MB), then restricted to lower dorsal ectoderm (G)	Inhibition	Goosecoid -> msx	Unknown		
	Goosecoid	ventral ectoderm (B, MB), then in a ring around the stomodeum (G)	Gain of function	onecut	Whole ectoderm early (blastula), then restricted to the ciliary band (late MB -> Pr)	Inhibition	Goosecoid -> onecut	Unknown		
	Goosecoid	ventral ectoderm (B, MB), then in a ring around the stomodeum (G)	Gain of function	tbx2/3	Dorsal ectoderm (LB->G)	Inhibition	Goosecoid -> tbx2/3	Unknown	not shown	
	Goosecoid	ventral ectoderm (B, MB), then in a ring around the stomodeum (G)	Gain of function	univin	Whole ectoderm early, then inhibited dorsally (MB) before being restricted to the lateral part of the ciliary band (G, Pr). At G stage, a weak expression persists in the ventral ectoderm.	Inhibition	Goosecoid -> univin	Unknown		
	Goosecoid	ventral ectoderm (B, MB), then in a ring around the stomodeum (G)	Gain of function	wnt8	ventral ectoderm + lateral ectoderm (MB), then restricted to the ciliary band (G, Pr)	Inhibition	Goosecoid -> wnt8	Unknown		
	FoxA	ventral ectoderm, then stomodeum (MB->Pr)	Loss of function	bmp2/4	ventral ectoderm (EB->G)	Independent	N/A	N/A	not shown	

A

Perturbed gene INPUT	Spatial domain (*) INPUT	ASSAY	Analyzed gene OUTPUT	Spatial domain (*) OUTPUT	Interaction deduced between INPUT and OUTPUT genes	Summary	Direct interaction ?	Comment	Additional reference on the interaction (*P. lividus*)
FoxA	ventral ectoderm, then stomodeum (MB->Pr)	Loss of function	brachyury	ventral ectoderm, then stomodeum	Activation	FoxA -> brachyury	Unknown		
FoxA	ventral ectoderm, then stomodeum (MB->Pr)	Loss of function	chordin	ventral ectoderm (SB->G)	Independent	N/A	N/A		
FoxA	ventral ectoderm, then stomodeum (MB->Pr)	Loss of function	foxA	ventral ectoderm, then stomodeum (MB->Pr)	Activation	FoxA -> foxA	Unknown		
FoxA	ventral ectoderm, then stomodeum (MB->Pr)	Loss of function	goosecoid	ventral ectoderm (B, MB), then in a ring around the stomodeum (G)	Independent	N/A	N/A	not shown	
Brachyury	ventral ectoderm, then stomodeum	Loss of function	foxA	ventral ectoderm, then stomodeum (MB->Pr)	Activation	Brachyury -> foxA	Unknown		
tbx2/3	Dorsal ectoderm (LB->G)	Loss of function	atbf1	Lower ventral + dorsal ectoderm (G)	Activation	tbx2/3 -> atbf1	Unknown		
tbx2/3	Dorsal ectoderm (LB->G)	Loss of function	brachyury	ventral ectoderm, then stomodeum	Independent	N/A	N/A		
tbx2/3	Dorsal ectoderm (LB->G)	Loss of function	chordin	ventral ectoderm (SB->G)	Independent	N/A	N/A		
tbx2/3	Dorsal ectoderm (LB->G)	Loss of function	dlx	dorsal ectoderm (MB, G)	Activation	tbx2/3 -> dlx	Unknown		
tbx2/3	Dorsal ectoderm (LB->G)	Loss of function	fgfA	Whole ectoderm early, then inhibited dorsally before being restricted to the ciliary band (MB, G, Pr)	Independent	N/A	N/A		
tbx2/3	Dorsal ectoderm (LB->G)	Loss of function	foxA	ventral ectoderm, then stomodeum (MB->Pr)	Independent	N/A	N/A		
tbx2/3	Dorsal ectoderm (LB->G)	Loss of function	glypican5	Dorsal ectoderm (MB, G, Pr)	Independent	N/A	N/A	not shown	
tbx2/3	Dorsal ectoderm (LB->G)	Loss of function	irxA	Dorsal ectoderm (MB), then upper dorsal ectoderm + stomodeum (G)	Activation	tbx2/3 -> irxA	Unknown		
tbx2/3	Dorsal ectoderm (LB->G)	Loss of function	msx	Dorsal ectoderm (MB), then restricted to lower dorsal ectoderm (G)	Activation	tbx2/3 -> msx	Unknown		
tbx2/3	Dorsal ectoderm (LB->G)	Loss of function	onecut	Whole ectoderm early (blastula), then restricted to the ciliary band (late MB -> Pr)	Independent	N/A	N/A		
tbx2/3	Dorsal ectoderm (LB->G)	Loss of function	pax2/5/8	Ciliary band (vegetal part) (G)	Independent	N/A	N/A		
tbx2/3	Dorsal ectoderm (LB->G)	Loss of function	smad6	Dorsal ectoderm (MB->G)	Activation	tbx2/3 -> smad6	Unknown	smad6 is partially lost in tbx2/3 morphants, because it is also a direct target of BMP2/4 signaling, and therefore part of its expression rely on an input upstream of tbx2/3	
tbx2/3	Dorsal ectoderm (LB->G)	Loss of function	wnt5	Lower dorsal ectoderm (MB, G)	Independent	N/A	N/A	not shown	
IrxA	Dorsal ectoderm (MB), then upper dorsal ectoderr	Loss of function	bmp1	Ventral ectoderm + Ciliary band (G, Pr)	Independent	N/A	N/A	not shown	
IrxA	Dorsal ectoderm (MB), then upper dorsal ectoderr	Loss of function	chordin	ventral ectoderm (SB->G)	Independent	N/A	N/A	not shown	
IrxA	Dorsal ectoderm (MB), then upper dorsal ectoderr	Loss of function	deadringer	Ventral ectoderm + Ciliary band (G, Pr)	Independent	N/A	N/A	not shown	
IrxA	Dorsal ectoderm (MB), then upper dorsal ectoderr	Loss of function	foxA	ventral ectoderm, then stomodeum (MB->Pr)	Independent	N/A	N/A	not shown	
IrxA	Dorsal ectoderm (MB), then upper dorsal ectoderr	Loss of function	gfi1	Ciliary band (lateMB, G, Pr)	Independent	N/A	N/A	not shown	
IrxA	Dorsal ectoderm (MB), then upper dorsal ectoderr	Loss of function	msx	Dorsal ectoderm (MB), then restricted to lower dor	Independent	N/A	N/A	not shown	
IrxA	Dorsal ectoderm (MB), then upper dorsal ectoderm + stomodeum (G)	Loss of function	onecut	Whole ectoderm early (blastula), then restricted to the ciliary band (late MB -> Pr)	Inhibition	IrxA -> onecut	Unknown	Irxa cannot account for the whole repression of onecut deduced from BMP2/4 morphants, because it occurs only in the upper dorsal ectoderm : other repressors are needed and predicted to be active at least in the lower dorsal ectoderm	
IrxA	Dorsal ectoderm (MB), then upper dorsal ectoderr	Loss of function	univin	Whole ectoderm early, then inhibited dorsally (MB	Independent	N/A	N/A	not shown	

A

Perturbed gene INPUT	Spatial domain (*) INPUT	ASSAY	Analyzed gene OUTPUT	Spatial domain (*) OUTPUT	Interaction deduced between INPUT and OUTPUT genes	Summary	Direct interaction ?	Comment	Additional reference on the interaction (*P. lividus*)
IrxA	Dorsal ectoderm (MB), then upper dorsal ectoderr	Loss of function	wnt5	Lower dorsal ectoderm (MB, G)	Independent	N/A	N/A	not shown	
Onecut	Whole ectoderm early (blastula), then restricted to the ciliary band (late MB -> Pr)	Loss of function	deadringer	Ventral ectoderm + Ciliary band (G, Pr)	Activation	Onecut -> deadringer	Unknown		
Onecut	Whole ectoderm early (blastula), then restricted to the ciliary band (late MB -> Pr)	Loss of function	foxG	Ventral ectoderm + border with ciliary band - stomodeum (MB, G), then restricted to the ciliary band only (LG/Pr)	Activation	Onecut -> foxG	Unknown		
Onecut	Whole ectoderm early (blastula), then restricted to the ciliary band (late MB -> Pr)	Loss of function	gfi1	Ciliary band (lateMB, G, Pr)	Activation	Onecut -> gfi1	Unknown		
Onecut	Whole ectoderm early (blastula), then restricted to the ciliary band (late MB -> Pr)	Loss of function	pax2/5/8	Ciliary band (vegetal part) (G)	Activation	Onecut -> pax2/5/8	Unknown		
Onecut	Whole ectoderm early (blastula), then restricted to the ciliary band (late MB -> Pr)	Loss of function	wnt5	Lower dorsal ectoderm (MB, G)	Independent	N/A	N/A	not shown	
Wnt8	ventral ectoderm + lateral ectoderm (MB), then restricted to the ciliary band (G, Pr)	Loss of function	nodal	Ventral ectoderm (64C->G)	Activation	Wnt8 -> nodal	Unknown	required for nodal expression maintenance (MB)	
chordin	ventral ectoderm (SB->G)	Loss of function	bmp2/4	ventral ectoderm (EB->G)	Activation		No	chordin inhibition leads to transient activation of BMP2/4 signaling in the ventral territory, resulting in Nodal inhibition, finally ending with the inhibition of Nodal downstream targets (Bmp2/4 included)	
chordin	ventral ectoderm (SB->G)	Loss of function	chordin	ventral ectoderm (SB->G)	Activation		No	chordin inhibition leads to transient activation of BMP2/4 signaling in the ventral territory, resulting in Nodal inhibition, finally ending with the inhibition of Nodal downstream targets (Bmp2/4 included)	
chordin	ventral ectoderm (SB->G)	Loss of function	goosecoid	ventral ectoderm (B, MB), then in a ring around the stomodeum (G)	Activation		No	chordin inhibition leads to transient activation of BMP2/4 signaling in the ventral territory, resulting in Nodal inhibition, finally ending with the inhibition of Nodal downstream targets (Bmp2/4 included)	
chordin	ventral ectoderm (SB->G)	Loss of function	msx	Dorsal ectoderm (MB), then restricted to lower dorsal ectoderm (G)	Activation		No	chordin inhibition leads to transient activation of BMP2/4 signaling in the ventral territory, resulting in Nodal inhibition, finally ending with the inhibition of Nodal downstream targets (Bmp2/4 included)	
chordin	ventral ectoderm (SB->G)	Loss of function	nodal	Ventral ectoderm (64C->G)	Activation		No	chordin inhibition leads to transient activation of BMP2/4 signaling in the ventral territory, resulting in Nodal inhibition, finally ending with the inhibition of Nodal downstream targets (Bmp2/4 included)	
chordin	ventral ectoderm (SB->G)	Loss of function	onecut	Whole ectoderm early (blastula), then restricted to the ciliary band (late MB -> Pr)	inhibition		No	chordin inhibition leads to transient activation of BMP2/4 signaling in the ventral territory, resulting in Nodal inhibition, finally ending with the inhibition of Nodal downstream targets (Bmp2/4 included)	
chordin	ventral ectoderm (SB->G)	Loss of function	tbx2/3	Dorsal ectoderm (LB->G)	Activation		No	chordin inhibition leads to transient activation of BMP2/4 signaling in the ventral territory, resulting in Nodal inhibition, finally ending with the inhibition of Nodal downstream targets (Bmp2/4 included) Tbx2/3 would be transiently extended when bBMP2/4 signaling expands.	Lapraz et al, 2009
chordin	ventral ectoderm (SB->G)	Loss of function	wnt5	Lower dorsal ectoderm (MB, G)	Activation		No	chordin inhibition leads to transient activation of BMP2/4 signaling in the ventral territory, resulting in Nodal inhibition, finally ending with the inhibition of Nodal downstream targets (Bmp2/4 included)	

A	Perturbed gene INPUT	Spatial domain (*) INPUT	ASSAY	Analyzed gene OUTPUT	Spatial domain (*) OUTPUT	Interaction deduced between INPUT and OUTPUT genes	Summary	Direct interaction ?	Comment	Additional reference on the interaction (*P. lividus*)
	smad6	Dorsal ectoderm (MB->G)	Gain of function	chordin	ventral ectoderm (SB->G)	Independent		Unknown	Smad6 seems to be able to antagonize BMP2/4 signaling but not Nodal signaling at the lower concentration, but a higher concentration results seemingly in Nodal loss of function	
	smad6	Dorsal ectoderm (MB->G)	Gain of function	msx	Dorsal ectoderm (MB), then restricted to lower dorsal ectoderm (G)	Inhibition		Unknown	Smad6 seems to be able to antagonize BMP2/4 signaling but not Nodal signaling at the lower concentration, but a higher concentration results seemingly in Nodal loss of function	
	smad6	Dorsal ectoderm (MB->G)	Gain of function	onecut	Whole ectoderm early (blastula), then restricted to the ciliary band (late MB -> Pr)	activation		Unknown	Smad6 seems to be able to antagonize BMP2/4 signaling but not Nodal signaling at the lower concentration, but a higher concentration results seemingly in Nodal loss of function	
	smad6	Dorsal ectoderm (MB->G)	Gain of function	foxA	ventral ectoderm, then stomodeum (MB->Pr)	Independent		Unknown	Smad6 seems to be able to antagonize BMP2/4 signaling but not Nodal signaling at the lower concentration, but a higher concentration results seemingly in Nodal loss of function	

B Perturbed gene INPUT	Spatial domain (*) INPUT	ASSAY	Analyzed gene OUTPUT	Spatial domain (*) OUTPUT	Interaction deduced from previous work	Summary	Direct interaction ?	Comment	Reference (*P. lividus*)
lefty	ventral ectoderm (from early blastula to G)	Gain of function	brachyury	ventral ectoderm, then stomodeum	inhibition	lefty -> brachyury	Unknown	Lefty is Nodal antagonist : Lefty over expression leads to Nodal signaling inhibition	Duboc et al, 2008
lefty	ventral ectoderm (from early blastula to G)	Gain of function	tubulin	ciliary band (LG and Pr). A weaker expression is present at the dorsal ectoderm apex	inhibition	lefty -> tubulin	Unknown	Lefty is Nodal antagonist : Lefty overexpression leads to Nodal signaling inhibition	Duboc et al, 2008
lefty	ventral ectoderm (from early blastula to G)	Gain of function	tbx2/3	Dorsal ectoderm (LB->G)	inhibition	lefty -> tbx2/3	Unknown	Lefty is Nodal antagonist : Lefty overexpression leads to Nodal signaling inhibition	Duboc et al, 2008
lefty	ventral ectoderm (from early blastula to G)	Gain of function	29D	dorsal ectoderm (Pr)	inhibition	lefty -> 29D	Unknown	Lefty is Nodal antagonist : Lefty overexpression leads to Nodal signaling inhibition	Duboc et al, 2008
lefty	ventral ectoderm (from early blastula to G)	Loss of function	brachyury	ventral ectoderm, then stomodeum	inhibition	lefty -> brachyury	Unknown	Lefty is Nodal antagonist : in Lefty morphants, Nodal is ectopically expressed in the whole ectoderm	Duboc et al, 2008
lefty	ventral ectoderm (from early blastula to G)	Loss of function	tbx2/3	Dorsal ectoderm (LB->G)	inhibition	lefty -> tbx2/3	Unknown	Lefty is Nodal antagonist : in Lefty morphants, Nodal is ectopically expressed in the whole ectoderm	Duboc et al, 2008
lefty	ventral ectoderm (from early blastula to G)	Loss of function	nodal	Ventral ectoderm (64C->G)	inhibition	lefty -> nodal	Unknown	Lefty is Nodal antagonist : in Lefty morphants, Nodal is ectopically expressed in the whole ectoderm	Duboc et al, 2008
lefty	ventral ectoderm (from early blastula to G)	Loss of function	lefty	ventral ectoderm (from early blastula to G)	inhibition	lefty -> lefty	Unknown	Lefty is Nodal antagonist : in Lefty morphants, Nodal is ectopically expressed in the whole ectoderm	Duboc et al, 2008
lefty	ventral ectoderm (from early blastula to G)	Loss of function	29D	dorsal ectoderm (Pr)	inhibition	lefty -> 29D	Unknown	Lefty is Nodal antagonist : in Lefty morphants, Nodal is ectopically expressed in the whole ectoderm	Duboc et al, 2008
Bmp2/4	ventral ectoderm (EB->G)	Loss of function	goosecoid	ventral ectoderm (B, MB), then in a ring around the stomodeum (G)	Independent	N/A	N/A		Lapraz et al, 2009
Bmp2/4	ventral ectoderm (EB->G)	Loss of function	glypican5	Dorsal ectoderm (MB, G, Pr)	Activation	Bmp2/4 -> glypican5	Unknown		Lapraz et al, 2009
Bmp2/4	ventral ectoderm (EB->G)	Loss of function	onecut	Whole ectoderm early (blastula), then restricted to the ciliary band (late MB -> Pr)	inhibition	Bmp2/4 -> onecut	Unknown		Lapraz et al, 2009
Bmp2/4	ventral ectoderm (EB->G)	Loss of function	delta	Neuron precursor (Pr)	Inhibition	Bmp2/4 -> delta	Unknown		Lapraz et al, 2009
Alk3/6 (BMP type I receptor)	Ubiquitous	Loss of function	goosecoid	ventral ectoderm (B, MB), then in a ring around the stomodeum (G)	Independent	N/A	N/A		Lapraz et al, 2009
Alk3/6 (BMP type I receptor)	Ubiquitous	Loss of function	delta	Neuron precursor (Pr)	Inhibition	Alk3/6 (BMP type I receptor) -> wnt8	Unknown		Lapraz et al, 2009
Bmp2/4	ventral ectoderm (EB->G)	Gain of function	glypican5	Dorsal ectoderm (MB, G, Pr)	Activation	Bmp2/4 -> glypican5	Unknown		Lapraz et al, 2009
Alk4/5/7 (Nodal type I receptor)	Ubiquitous	Gain of function	chordin	ventral ectoderm (SB->G)	Activation	Alk4/5/7 (Nodal type I receptor) -> chordin	Yes	see inductive assay in presence of translational inhibitor	Lapraz et al, 2009
Nodal	Ventral ectoderm (64C->G)	Loss of function	nodal	Ventral ectoderm (64C->G)	Activation	Nodal -> nodal	Yes	supported by promoter analysis and inductive assay with translational inhibitor	Range and Lapraz et al, 2007
Alk4/5/7 (Nodal type I receptor)	Ubiquitous	Loss of function	nodal	Ventral ectoderm (64C->G)	Activation	Alk4/5/7 (Nodal type I receptor) -> nodal	Yes	supported by promoter analysis and inductive assay with translational inhibitor	Range and Lapraz et al, 2007
Alk4/5/7 (Nodal type I receptor)	Ubiquitous	Loss of function	univin	Whole ectoderm early, then inhibited dorsally (MB) before being restricted to the lateral part of the ciliary band (G, Pr). At G stage, a weak expression persists in the ventral ectoderm.	Independent	N/A	N/A		Range and Lapraz et al, 2007
Univin	Whole ectoderm early, then inhibited dorsally (MB) before being restricted to the lateral part of the ciliary band (G, Pr). At G stage, a weak expression persists in the ventral ectoderm.	Gain of function	nodal	Ventral ectoderm (64C->G)	Activation	Univin -> nodal	Unknown		Range and Lapraz et al, 2007

B	Perturbed gene INPUT	Spatial domain (*) INPUT	ASSAY	Analyzed gene OUTPUT	Spatial domain (*) OUTPUT	Interaction deduced from previous work	Summary	Direct interaction ?	Comment	Reference (*P. lividus*)
	SoxB1	Whole ectoderm	Loss of function	nodal	Ventral ectoderm (64C->G)	Activation	SoxB1 -> nodal	Unknown		Range and Lapraz et al, 2007
	FGFA	Whole ectoderm early, then inhibited dorsally before being restricted to the ciliary band (MB, G, Pr)	Loss of function	pax2/5/8	Ciliary band (vegetal part) (G)	Activation	FGFA -> pax2/5/8	Unknown		Röttinger et al, 2008
	FGFA	Whole ectoderm early, then inhibited dorsally before being restricted to the ciliary band (MB, G, Pr)	Loss of function	sprouty	Ciliary band (lateral part)	Activation	FGFA -> sprouty	Unknown		Röttinger et al, 2008

(*) with respect to the ectoderm territories only, some genes are expressed in different germ layers and this will not be listed here
