# Supplementary material for: Ancestral Regulatory Circuits Governing Ectoderm Patterning Downstream of Nodal and BMP2/4 Revealed by Gene Regulatory Network Analysis in an Echinoderm
Source: PLoS Genet. 2010 Dec 23;6(12):e1001259. doi: 10.1371/journal.pgen.1001259 (PMC3009687; doi:10.1371/journal.pgen.1001259)
Supplement: Table S2 — Gene knockdown without detectable effect as examined by in situ hybridization. (0.06 MB PDF) [file pgen.1001259.s007.pdf]

Gene knockdown without detectable effect as examined by in situ hybridization

perturbation	marker gene	stage	interaction
Mo1 dri	brachuyry	EG and G	none
	chordin	EG	none
	egip	G	none
	fgfA	G	none
	foxA	EG	none
	foxG	G	none
	gfi1	G	none
	goosecoid	EG and G	none
	irxA	G	none
	nk1	EG	none
	nk2.2	EG	none
	onecut	G	none
	otx	G	none
	pax2/5/8	G	none
	tbx2/3	EG	none
	tubulin	G	none
	univin	G	none
	wnt8	G	none
Mo1 FoxG	brachyury	MB	none
	chordin	MB	none
	fgfA	G	none
	foxA	MB	none
	foxA	G	none
	goosecoid	MB	none
	nk2.2	MB	none
	nodal	MB	none
	onecut	G	none
	pax2/5/8	G	none
	tbx2/3	MB	none
	univin	G	none
	wnt8	G	none
Mo1 Hox7	atbf1	G	none
	chordin	G	none
	fgfA	G	none
	irxA	G	none
	onecut	G	none
Mo1 Msx	atbf1	G	none
	brachyury	MB and G	none
	chordin	MB	none
	dlx	G	none
	foxA	MB and G	none
Mo1 Msx	glypican5	G	none
	irxA	G	none
	msx	G	none
	nk2.2	MB	none
	nodal	MB	none
	onecut	G	none
	pax2/5/8	G	none
	tbx23	MB	none
	wnt5	G	none
	chordin	G	none
	dri	G	none
	foxA	G	none
	goosecoid	G	none
	nk1	G	none

perturbation	marker gene	stage	interaction
Mo2 Msx	atbf1	G	none
	bmp24	MB	none
	brachuyry	MB	none
	chordin	MB	none
	dlx	G	none
	foxA	MB	none
	irxA	G	none
	msx	G	none
	onecut	G	none
	pax2/5/8	G	none
	smad6	MB	none
	smad6	G	none
	tbx2/3	MB	none
	tbx23	G	none
Mo2 NK2.2	brachyury	EG	none
	chordin	EG	none
	foxA	EG	none
	msx	EG	none
	onecut	EG	none
	smad6	EG	none
	tbx23	EG	none
Mo1 Oasis	atbf1	G	none
	chordin	MB	none
	dlx	G	none
	irxA	G	none
	msx	G	none
	nk2.2	G	none
	onecut	G	none
	smad6	MB	none
	tbx2/3	MB	none
	wnt5	G	none
	wnt8	G	none
Mo1 Wnt5	atbf1	G	none
	bmp2/4	MB	none
	brachyury	G	none
	chordin	MB	none
	dlx	G	none
	dri	MB	none
	foxA	G	none
	goosecoid	MB	none
	irxA	G	none
	msx	G	none
	nk2.2	MB	none
	nodal	MB	none
	onecut	G	none
	pax2/5/8	G	none
	Smad6	MB	none
	tbx2/3	MB	none
	wnt5	G	none
